# Exploring the anticancer and antibacterial potential of naphthoquinone derivatives: a comprehensive computational investigation

**DOI:** 10.3389/fchem.2024.1351669

**Published:** 2024-02-21

**Authors:** Mehnaz Hossain Meem, Sumaiya Binte Yusuf, Sanaa S. Al Abbad, Shofiur Rahman, Mahmoud Al-Gawati, Hamad Albrithen, Abdullah N. Alodhayb, Kabir M. Uddin

**Affiliations:** ^1^ Department of Biochemistry and Microbiology, North South University, Dhaka, Bangladesh; ^2^ Department of Chemistry, Imam Abdulrahman Bin Faisal University, Dammam, Saudi Arabia; ^3^ Biological and Environmental Sensing Research Unit, King Abdullah Institute for Nanotechnology, King Saud University, Riyadh, Saudi Arabia; ^4^ Research Chair for Tribology, Surface, and Interface Sciences, Department of Physics and Astronomy, College of Science, King Saud University, Riyadh, Saudi Arabia

**Keywords:** naphthoquinone, DFT, ADMET, molecular docking, MD simulation, PCA

## Abstract

This study investigates the potential of 2-(4-butylbenzyl)-3-hydroxynaphthalene-1,4-dione (**11**) and its 12 derivatives as anticancer and biofilm formation inhibitors for methicillin-resistant *staphylococcus aureus* using *in silico* methods. The study employed various computational methods, including molecular dynamics simulation molecular docking, density functional theory, and global chemical descriptors, to evaluate the interactions between the compounds and the target proteins. The docking results revealed that compounds **9**, **11**, **13**, and ofloxacin exhibited binding affinities of −7.6, −7.9, −7.5, and −7.8 kcal mol^−1^, respectively, against peptide methionine sulfoxide reductase msrA/msrB (PDB: 3E0M). Ligand (**11**) showed better inhibition for methicillin-resistant *staphylococcus aureus* msrA/msrB enzyme. The complex of the 3E0M-ligand **11** remained highly stable across all tested temperatures (300, 305, 310, and 320 K). Principal Component Analysis (PCA) was employed to evaluate the behavior of the complex at various temperatures (300, 305, 310, and 320 K), demonstrating a total variance of 85%. Convergence was confirmed by the eigenvector’s cosine content value of 0.43, consistently displaying low RMSD values, with the minimum observed at 310 K. Furthermore, ligand **11** emerges as the most promising candidate among the compounds examined, showcasing notable potential when considering a combination of *in vitro*, *in vivo*, and now *in silico* data. While the naphthoquinone derivative (**11**) remains the primary candidate based on comprehensive *in silico* studies, further analysis using Frontier molecular orbital (FMO) suggests while the Egap value of compound **11** (2.980 eV) and compound **13** (2.975 eV) is lower than ofloxacin (4.369 eV), indicating their potential, so it can be a statement that compound **13** can also be investigated in further research.

## Graphical Abstract

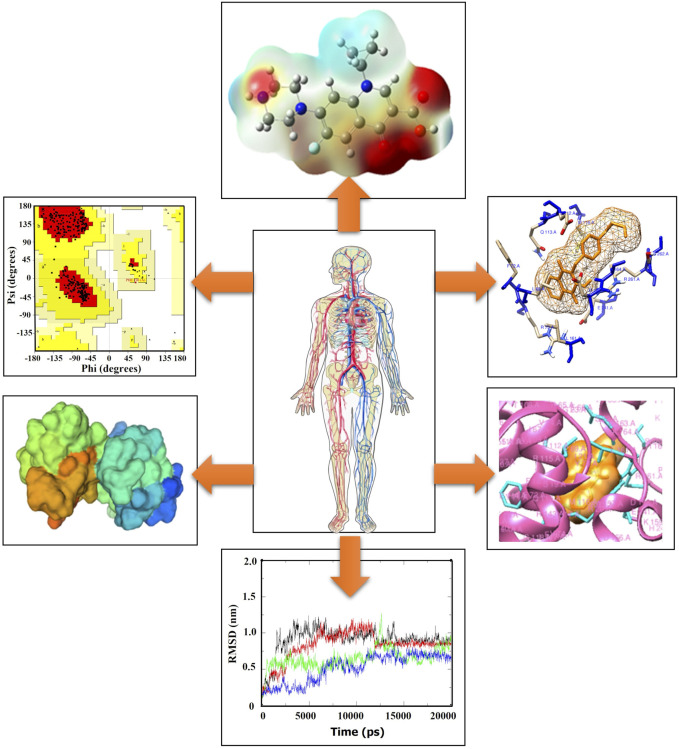

## 1 Introduction

Naphthoquinones have emerged as a pivotal compound class in drug development, celebrated for their versatility in synthesizing bioactive compounds. These compounds demonstrate a range of biological activities, including antimicrobial, antitumor, antioxidant, antimalarial, and neuroprotective effects, primarily attributed to their distinctive redox properties (Fiorito et al., 2016; Malik et al., 2021). Their electron-accepting capabilities form highly reactive radicals that interact with crucial biological molecules like DNA, enzymes, and proteins, a central aspect contributing to their efficacy in various therapeutic applications. The escalating global health crisis posed by multidrug-resistant bacteria, particularly Methicillin-resistant *staphylococcus aureus* (MRSA), has spurred research into alternative therapeutic agents. Notably, naphthoquinones, especially 1,4-naphthoquinone and its derivatives, exhibit promise in this context. Their antibacterial potential against resistant strains underscores their significance in current medical research, providing an innovative approach to address this urgent health concern (Stefani et al., 2012; Coenen et al., 2013).

**FIGURE 1:**
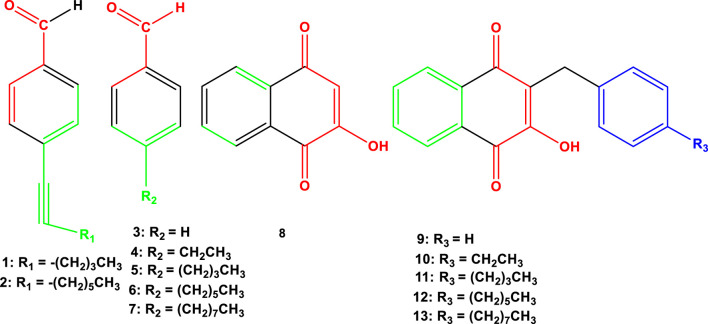
Chemical structures of novel naphthoquinone derivatives (**1–13**).

Our research concentrates explicitly on the synthesis and assessment of derivatives of lawsone, a naturally occurring hydroxynaphthoquinone within the naphthoquinone family. These derivatives manifest a spectrum of biological activities, encompassing antifungal, antitumor, and antiviral properties (Deurenberg and Stobberingh, 2008; Stefani and Goglio, 2010). Their mechanisms of action as antibacterial agents are diverse, involving the disruption of protein and nucleic acid synthesis, modulation of redox processes, and induction of apoptosis in bacterial cells (Loomba et al., 2010; Rossi et al., 2014; Wang et al., 2022). The wide range of activities underscores the potential of lawsone derivatives as versatile therapeutic agents. In the field of cancer treatment, naphthoquinones have exhibited significant potential. They have been identified to target multiple molecular pathways in cancerous cells, leading to apoptosis and inhibition of cell growth. This mode of action, combined with their capacity to inhibit crucial cellular processes selectively, positions them as promising candidates in oncology (Harada et al., 2014; Brixius-Anderko and Schott, 2019). The versatility of naphthoquinones is further emphasized by their capability to interfere with the electron transport chain in both eukaryotic and prokaryotic cells, highlighting their potential to address a broad range of diseases. Figure 1 illustrates the chemical structures of the 13 naphthoquinone derivatives which has been utilized, obtained from Song R et al. (2020).

Alongside lab experiments, computer-based studies have been vital in exploring the potential of naphthoquinone derivatives. These virtual investigations give us insights into how naphthoquinones interact with different biological targets, helping us identify promising leads for further experiments (Goldberg et al., 2015; Jahanbad and Esfahlan, 2017; Khosla et al., 2022). Our study uses these computer techniques to evaluate new naphthoquinone derivatives. We analyze their physical and chemical properties, how they move through the body, and any potentially harmful effects, aiming to find candidates with the best antibacterial and anticancer properties (Sahoo et al., 2022; Rahman et al., 2022). Uddin et al.’s research highlights certain chemical derivatives’ potential cancer-fighting abilities (Esha et al., 2023; Uddin et al., 2023). The first study (Uddin et al., 2023) looked into benzylidene malononitrile and ethyl 2-Cyano-3-phenylacrylate derivatives, while the next one (Esha et al., 2023) focused on fluoro flavonoid derivatives. Both studies provide valuable insights into the anticancer properties of these compounds. Using computer simulations, the second study (Esha et al., 2023) gives a computational perspective that can guide more experiments and potentially have applications in medicine and biology.

**FIGURE 2:**
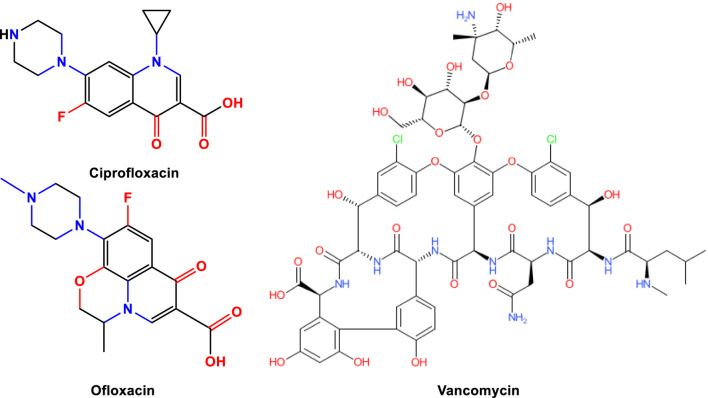
Chemical structures of ciprofloxacin, ofloxacin, and vancomycin.

In this study, we performed *in silico* investigations to assess the potential of recently developed naphthoquinone derivatives (**1**–**13**) in comparison to established pharmaceutical compounds—specifically, ciprofloxacin (D1), ofloxacin (D2), and vancomycin (D3) (Figures 1, 2) as potential agents with antibacterial and anticancer properties. Various computational techniques, including molecular docking, PASS predictions, molecular dynamics simulations, and DFT calculations, were utilized to scrutinize their physicochemical properties, pharmacokinetic and pharmacodynamics profiles, and potential toxicological effects. Additionally, molecular dynamics simulation analyses were conducted on the active site of protein-ligand interactions to evaluate the stability of the protein-ligand complexes. The outcomes of these studies suggest that these derivatives represent a promising novel class of anticancer and antibacterial agents, emphasizing the need for further exploration in drug development. In summary, naphthoquinones constitute a significant and versatile class of compounds in the pharmaceutical landscape, showing promise in combating multidrug-resistant bacteria and various types of cancer. Our research contributes to this expanding field by synthesizing and evaluating new derivatives, aiming to develop effective therapeutic agents against these challenging medical conditions.

## 2 Methodology

### 2.1 Computational analysis

We conducted extensive computational analyses to gain insights into the characteristics of the synthesized naphthoquinone derivatives utilizing Gaussian16 software (Frisch et al., 2019). Molecular geometry optimization, executed at the B3LYP/6-31G (d,p) level of theory, yielded optimized structural parameters, as elaborated in Supplementary Tables S1–S16. In a prior investigation, Uddin et al. (2023) comprehensively explored the structural parameters, including bond lengths and angles, for seven compounds. The calculations were conducted at the B3LYP level of theory, utilizing various basis sets, namely, 6-31G(d,p), 6-311G(d,p), and 6-311++G(d,p). Their findings indicated that B3LYP/6-31G(d,p) exhibited good agreement with experimental results. Furthermore, Frontier Molecular Orbital (FMO) analysis was undertaken to elucidate the energy levels and spatial distributions of the HOMO (highest occupied molecular orbital) and LUMO (lowest unoccupied molecular orbital). These Frontier orbitals, positioned at the outermost boundary of the molecule, play a pivotal role in determining its electronic behavior. The HOMO energy reflects the molecule’s electron-donating ability, while the LUMO energy characterizes its electron-accepting potential. An ideal scenario is characterized by a high HOMO value and a low LUMO value, indicating favorable electron transfer properties. Gaussview 6 software (Dennington et al., 2016) was employed to visualize the electrostatic potential distribution across the molecules. Molecular electrostatic potential (MEP), represented through a spectrum of colors, reveals the net electrostatic potential arising from the total charge distribution at nucleophilic and electrophilic sites within a compound. Electrophilic regions, indicating the strongest attraction, are depicted in blue, while nucleophilic areas, signifying the highest repulsion, are colored red. Green zones represent a neutral electrostatic potential. Additionally, the dipole moments of the compounds were calculated using Gaussview 6. The FMO energy gap, representing molecular stability, is determined by the difference between HOMO and LUMO energies. The ligands’ overall chemical reactivity descriptors, including ionization potential (IP), chemical potential (µ), electron binding energy (H), maximal charge acceptance (∆Nmax), global chemical hardness (η), global chemical softness (σ), energy change (∆E), electrophilicity (ω), electronegativity (χ), and electron affinity (EA) (Alberty, 1960; Chamizo et al., 1993; Parr et al., 1999; Elkaeed et al., 2022), were computed using these HOMO and LUMO values. The formulas for these descriptors are as follows:

$$E_{\text{Gap}}\;\left(\text{eV}\right)=\left(E_{\text{LUMO}}\;-\;E_{\text{HOMO}}\right);\text{ IP }\left(\text{eV}\right)=-E_{\text{HOMO}};\text{ EA }\left(\text{eV}\right)=-E_{\text{LUMO}};\;\mu \;\left(\text{eV}\right)=\left(\text{IP}+\text{EA}\right)/2$$

$$\chi =-\;\mu ;\;\eta =\left(\text{IP }-\text{ EA}\right)/2;\text{ and }\sigma =1/\eta ;\;\omega =\mu^{2}/2\eta$$

### 2.2 Analyzing of physicochemical and pharmacokinetic properties

The swissADME server tool (www.swissadme.ch) (Daina et al., 2017) was employed to assess the effectiveness of napthoquinone derivatives (**1–13**) and determine their physicochemical properties. Data were gathered from PubChem, the research database https://pubchem.ncbi.nlm.nih.gov/search/search.cgi (Kim et al., 2021), and a previous research paper. This tool provides accurate predictive results for identifying physicochemical properties, medicinal chemistry, and drug-likeness (Daina et al., 2017). Additionally, the AdmetSAR tool (http://lmmd.ecust.edu.cn/admetsar2/) (Yang et al., 2019) and ADMET predictor software were utilized to analyze ADMET properties. Using each compound’s canonical simplified molecular input line entry system (SMILES), various values, including multiple toxicity values, CYP inhibitors, and hERG pIC50 values, were determined. AdmetSAR offers both unrestricted and restricted values for each ADMET property. Furthermore, the Molinspiration online open-access server (https://www.molinspiration.com/cgi-bin/properties) (Molinspiration cheminformatics software, Choice reviews online. 2006) was employed to evaluate the correlation between the physicochemical properties and molecular activity of the compounds. The drug-likeness of the tested compounds was assessed as G-protein coupled receptor (GPCR) ligands, ion channel modulators (ICM), kinase inhibitors (KI), nuclear receptor ligands (NRL), protease inhibitors (PI), and enzyme inhibitors (EI).

### 2.3 Molecular docking

#### 2.3.1 Preparation of ligands

The reference pharmaceutical compounds, specifically ciprofloxacin, ofloxacin, and vancomycin, were obtained from the PubChem database in SDF (structure-data file) format (Kim et al., 2016). The naphthoquinone derivatives’ three-dimensional (3D) molecular structures were also acquired in SDF format from PubChem. Subsequently, the structural optimization of all naphthoquinone derivatives was conducted using the B3LYP/6-31G (d,p) computational method with the Gaussian 16 software package. The visualization and representation of these naphthoquinone structures were achieved using GaussView 6 software. To ensure the geometric stability and conformational accuracy of the ligands chosen for molecular docking, energy minimization (EM) procedures were applied. Following this, the conversion of these optimized structures into the PDBQT (Protein Data Bank, Partial Charge, and Atom Type) format was carried out using the OpenBabel plugin integrated within PyRx 0.8 software, available at https://pyrx.sourceforge.io/(Dallakyan and Olson, 2015). This involved retrieving reference pharmaceutical compounds and naphthoquinone derivatives in specific file formats, optimizing their structures, and converting them for subsequent molecular docking studies.

#### 2.3.2 Preparation of target proteins

We employed molecular docking software to identify a protein suitable for potential ligand binding, utilizing proteins obtained from the RCSB Protein Data Bank (https://www.rcsb.org/) (Berman et al., 2000; Zardecki et al., 2016). This study selected four proteins as target proteins: Peptide methionine sulfoxide reductase msrA/msrB (PDB: 3E0M), the Middle East respiratory syndrome coronavirus (MERS-CoV) papain-like protease (PLpro) (PDB: 4RNA), Epidermal Growth Factor Receptor (4UV7), Mevalonate diphosphate decarboxylase (PDB: 4DPT). The quality of these protein structures was assessed using Ramachandran plots through the SAVESv6.0 server available at https://saves.mbi.ucla.edu/(Laskowski et al., 2013). Additionally, ProSA was utilized to calculate the Z-scores for these proteins (Rathod et al., 2022). To ensure optimal performance in molecular docking studies, structural optimization of the selected proteins was carried out using Chimera version 1.16 (Goddard et al., 2007). The Chimera 1.16 Dock Prep tool was used to prepare the proteins for docking, involving the addition of a +2 charge to the heme group, assignment of charges to standard residues using AMBER ff14SB, utilization of Gasteiger charges for other residues, and addition of hydrogen atoms. The preparation of peptide methionine sulfoxide reductase msrA/msrB (PDB: 3E0M) followed a similar process, except not removing any chains.

#### 2.3.3 Protein-ligand docking

In our study, we employed AutoDock Vina software for protein-ligand docking. We focused our docking investigations on the target protein and various ligands. To encompass the entire protein, we established a grid box centered on it. We must note that we ensured the stability of both proteins and ligands throughout the entire docking process. Additionally, we validated the protein-ligand complexes by conducting re-docking experiments. To identify the amino acids interacting with the ligands, we employed UCSF Chimera version 1.16 (Goddard et al., 2007). Three-dimensional structures for molecular docking images were generated using Pymol version 2.5 and Chimera 1.16. Finally, the BIOVIA Discovery Studio (Baroroh et al., 2023) visualizer facilitated the visualization of binding modes in protein-ligand interactions, enabled the examination of 2D protein interactions with the ligands, and assisted in determining hydrogen density around residues engaged in interactions with the protein.

### 2.4 Molecular dynamics simulation

Molecular dynamics simulations were carried out using Galaxy Europe to investigate the interactions between ligands **9**, **11**, **13**, and ofloxacin as reference drugs and the chain of 3E0M. These simulations were conducted with GROMACS version 2021.6 (Van Der Spoel et al., 2005) and the AMBER99SB force field (Hansson et al., 2002; Showalter and Brüschweiler, 2007), offering a detailed account of atomic interactions. Molecular dynamics simulations are a robust computational technique, surpassing less computationally intensive docking methods. They provide high precision and offer insights into complex system behavior, often inaccessible through experimental means (Hansson et al., 2002; Showalter and Brüschweiler, 2007). In this study, we performed additional MD simulations for compounds **9**, **11**, **13**, and ofloxacin with the A chain of 3E0M to validate previous findings. The protein topology parameters were generated using the Galaxy European Server (Bray et al., 2020), a widely used molecular modeling and simulation platform. The simulation parameters included the TIP3P water model, and hydrogen atoms were initially excluded from the GROMACS setup. Hydrogens were added to the ligand at pH 7.4, and during MD topology generation, the molecule’s charge was maintained at 0 with a multiplicity of 1. The gaff force field was applied for parameterization. The ligand and protein files were combined, and a structural configuration was established within a 1 nm triclinic box. The system was solved with SPC water molecules in a triclinic box, and sodium and chloride ions were introduced to attain standard salt concentrations and neutralize the system (Presti et al., 2016).

An equilibration process was conducted to ensure system stability, utilizing position-restrained dynamics (NVT) at 300 K for 3,000 ps, implemented with the leapfrog algorithm (Cuendet and van Gunsteren, 2007; Esha et al., 2023; Uddin et al., 2023). Following this equilibration phase, the system underwent a production run for an additional 3,000 ps under constant temperature and pressure conditions. Subsequently, the system was further simulated for 20 ns at the same temperature and pressure. Various GROMACS utility tools, including gmx rmsd, gmx gyrate, gmx rmsf, and ‘gmx hbond’, were employed to generate graphs for RMSD, radius of gyration, RMSF, and hydrogen bonds of the ligands at 300 K over the 20 ns period. Furthermore, principal component analysis (PCA) was utilized to assess the stability of protein complexes, proving highly advantageous (Biswal et al., 2023; Panda et al., 2023). PCA was carried out for the ligand-protein complexes (**9**, **11**,**13**, and ofloxacin) at 300 K, as well as for compound **11** at temperature settings of 300 K, 305 K, 310 K, and 320 K, using the Bio3D package of GALAXY Europe server (Grant et al., 2006; Afgan et al., 2018; Kumar et al., 2022). The cosine content of the ligands was also evaluated. These MD simulations have provided valuable insights into the behavior of the protein-ligand complexes, aiding in a better comprehension of the underlying physical principles governing the structure and function of biological macromolecules.

## 3 Results and discussion

### 3.1 Analysis of frontier molecular orbitals (FMO)

**FIGURE 3:**
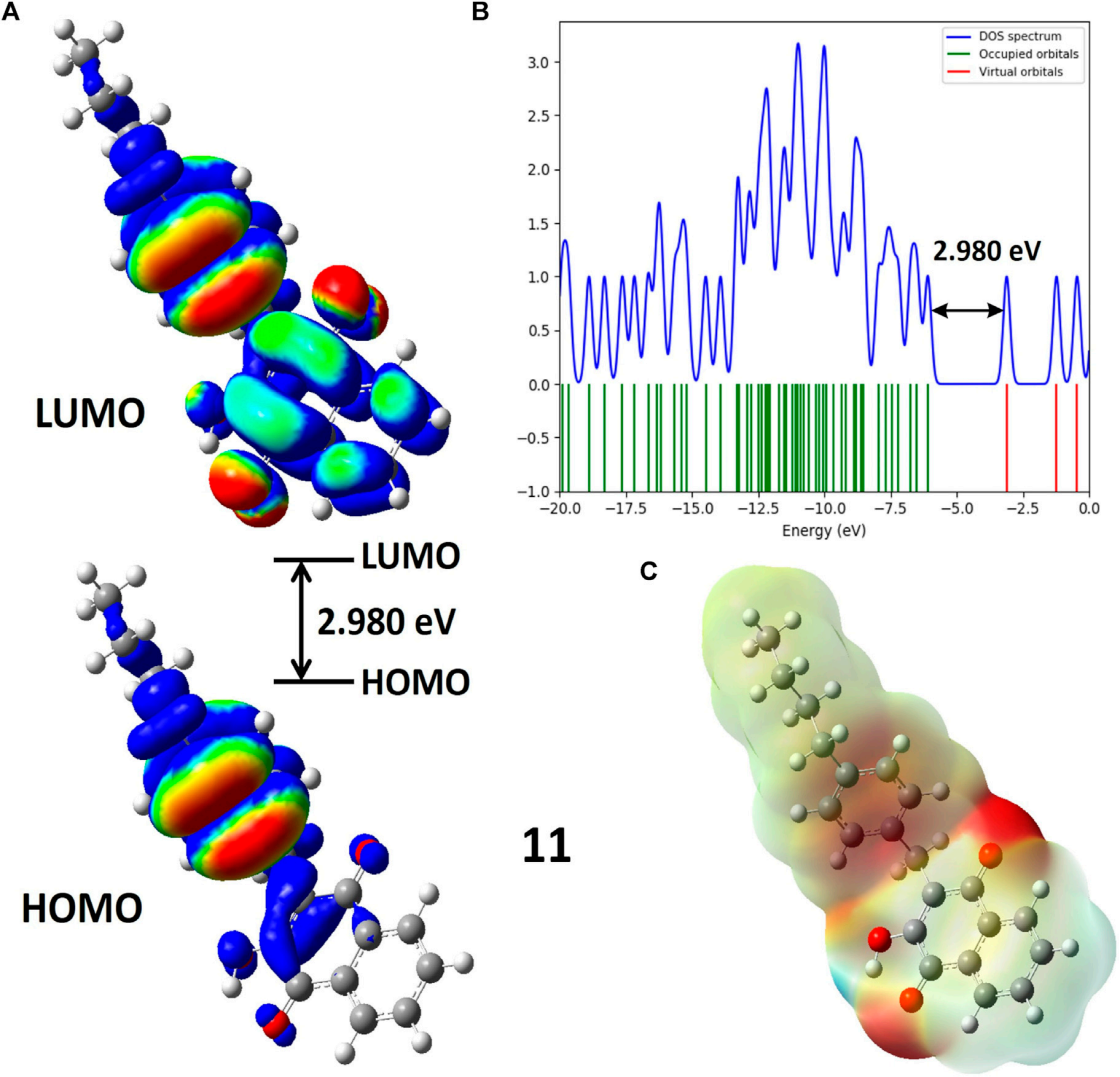
**(A)** Molecular orbitals of isodensity surfaces (0.02 electrons Bohr^−3^ surface) (red = electron-rich, blue = electron-deficient) of HOMO and LUMO; **(B)** DOS plot and HOMO-LUMO energy gap; **(C)** Maps of electrostatic potential (0.02 electrons Bohr^−3^ surface) (red = electron-rich, blue = electron-deficient) for the compound **11.**

Evaluating naphthoquinone and its derivatives (**1**–**13**) through fragment molecular analysis (FMO) has provided valuable insights into their chemical reactivity and stability. A broader energy gap (Egap) indicates heightened stability and reduced reactivity, whereas a narrower gap signifies softness, corresponding to increased reactivity and diminished stability. In addition to Egap, the energy levels of the Highest Occupied Molecular Orbital (HOMO) and Lowest Unoccupied Molecular Orbital (LUMO) play a significant role in understanding the electron-donating and -accepting characteristics of these compounds. To elaborate further, compounds with a narrow energy gap are classified as “soft” (σ), indicating high chemical reactivity and low stability, while those with a wider gap are labeled as “hard” (η). In this investigation, various molecular properties, such as ionization potential (IP), chemical potential (µ), electron binding energy (H), global chemical hardness (η), global chemical softness (σ), energy change (∆E), electrophilicity (ω), electronegativity (χ), electron affinity (EA), and dipole moment, were computed using the B3LYP/6-31G (d,p) method. The results of these calculations are presented in Table 1 and illustrated in Figure 3 and Supplementary Figures S1–S15.

**TABLE 1:** Molecular orbital (MO) data: energy gap (Egap), ionization potential (IP), electron affinity (EA), binding energy (H), electronegativity (χ), chemical potential (μ), global hardness (η), softness (σ), electrophilicity (ω), and dipole moment (Debye) for compounds (**1–13**) at 298.15 K.^a^

Ligand	E_LUMO_ (eV)	E_HOMO_ (eV)	E_gap_ (eV)	IP (eV)	EA (eV)	Μ (eV)	χ (eV)	μ (eV)	η (eV)	σ (eV)	ω (eV)
**1**	−6.362	−1.904	4.458	6.362	1.904	4.133	−4.133	4.133	2.229	0.448	3.832
**2**	−6.354	−2.000	4.448	6.354	2	4.13	−4.13	4.13	2.224	0.449	3.834
**3**	−6.944	−1.726	5.218	6.944	1.726	4.335	−4.335	4.335	2.609	0.383	3.601
**4**	−6.839	−1.614	5.225	6.839	1.614	4.226	−4.226	4.226	2.612	0.382	3.418
**5**	−6.825	−1.598	5.227	6.825	1.598	4.211	−4.211	4.211	2.613	0.382	3.393
**6**	−6.821	−1.594	5.227	6.821	1.594	4.207	−4.207	4.207	2.613	0.382	3.386
**7**	−6.820	−1.593	5.227	6.82	1.593	4.206	−4.206	4.206	2.613	0.382	3.385
**8**	−7.174	−3.176	3.998	7.174	3.176	5.175	−5.175	5.175	1.999	0.500	6.698
**9**	−6.340	−3.138	3.202	6.34	3.138	4.739	−4.739	4.739	1.601	0.624	7.013
**10**	−6.113	−3.115	2.998	6.113	3.115	4.614	−4.614	4.614	1.499	0.667	7.101
**11**	−6.092	−3.112	2.980	6.092	3.112	4.602	−4.602	4.602	1.49	0.671	7.106
**12**	−6.088	−3.111	2.977	6.088	3.111	4.599	−4.599	4.599	1.488	0.672	7.107
**13**	−6.086	−3.111	2.975	6.086	3.111	4.598	−4.598	4.598	1.487	0.672	7.108
**D1**	−5.703	−1.187	4.516	5.703	1.187	3.445	−3.445	3.445	2.258	0.442	2.627
**D2**	−5.625	−1.256	4.369	5.625	1.256	3.44	−3.44	3.44	2.184	0.457	2.709
**D3**	−5.734	−5.232	0.502	5.734	5.232	5.483	−5.483	5.483	0.251	3.984	59.88

^a^ Calculated by: HOMO, energy (E_LUMO_), LUMO, energy (E_LUMO_), Energy gap (E_gap_) = E_LUMO_ − E_HOMO_.

Ionization potential (IP) = −E_HOMO_, Electron affinity (EA) = −E_LUMO_, Electronegativity (χ) = (IP + EA)/2, Chemical potential (μ) = −χ, Hardness (η) = (IP − EA)/2, Softness (σ) = 1/η, Electrophilicity (ω) = μ2/2η.

Table 1 indicates that compounds **9**, **10**, **11**, **12**, **13**, and D2 (ofloxacin) exhibited energy gaps (Egap) of 3.202 eV, 2.998 eV, 2.980 eV, 2.977 eV, 2.975 eV, and 4.369 eV, respectively. Among these, compounds **9** to **13** displayed low energy gaps, suggesting good reactivity and stability, requiring less energy for electron promotion from HOMO to LUMO orbital. Compared to D2, their energy gaps were lower, indicating greater stability and a willingness to participate in reactions. Compound **9** had the highest Egap of 3.202 eV, signifying lower reactivity but increased hardness and stability. Furthermore, compound **13** showed the lowest HOMO-LUMO gap (2.975 eV) with a dipole moment of 2.504 D, implying efficient biological activities. The energy gap values followed this order: compound **9** (3.202 eV) > compound **10** (2.988 eV) > compound **11** (2.98 eV) > compound **12** (2.977 eV) > compound **13** (2.975 eV). Compounds **9**, **11**, and **13** were chosen for further evaluation through additional computational analysis and molecular dynamics.

Figure 3 illustrates Molecular Electrostatic Potential (MEP) maps for compound **11**. Electrophilic sites (depicted in blue) function as electron acceptors, while nucleophilic sites (shown in red) serve as electron donors. Partially nucleophilic regions are represented in yellow. The color-coded electrostatic potential highlights negative potential (in red, orange, and yellow shades) around electronegative atoms such as oxygen, indicating electrophilic reactivity. Conversely, positive potential (in blue) is observed over hydrogen atoms, signaling nucleophilic reactivity. Green areas denote the neutral potential. MEP maps provide insights into molecular stability by indicating consistent potential and are also helpful in studying molecular recognition and understanding interactions based on electrostatic complementarity.

### 3.2 Molecular docking calculation

**FIGURE 4:**
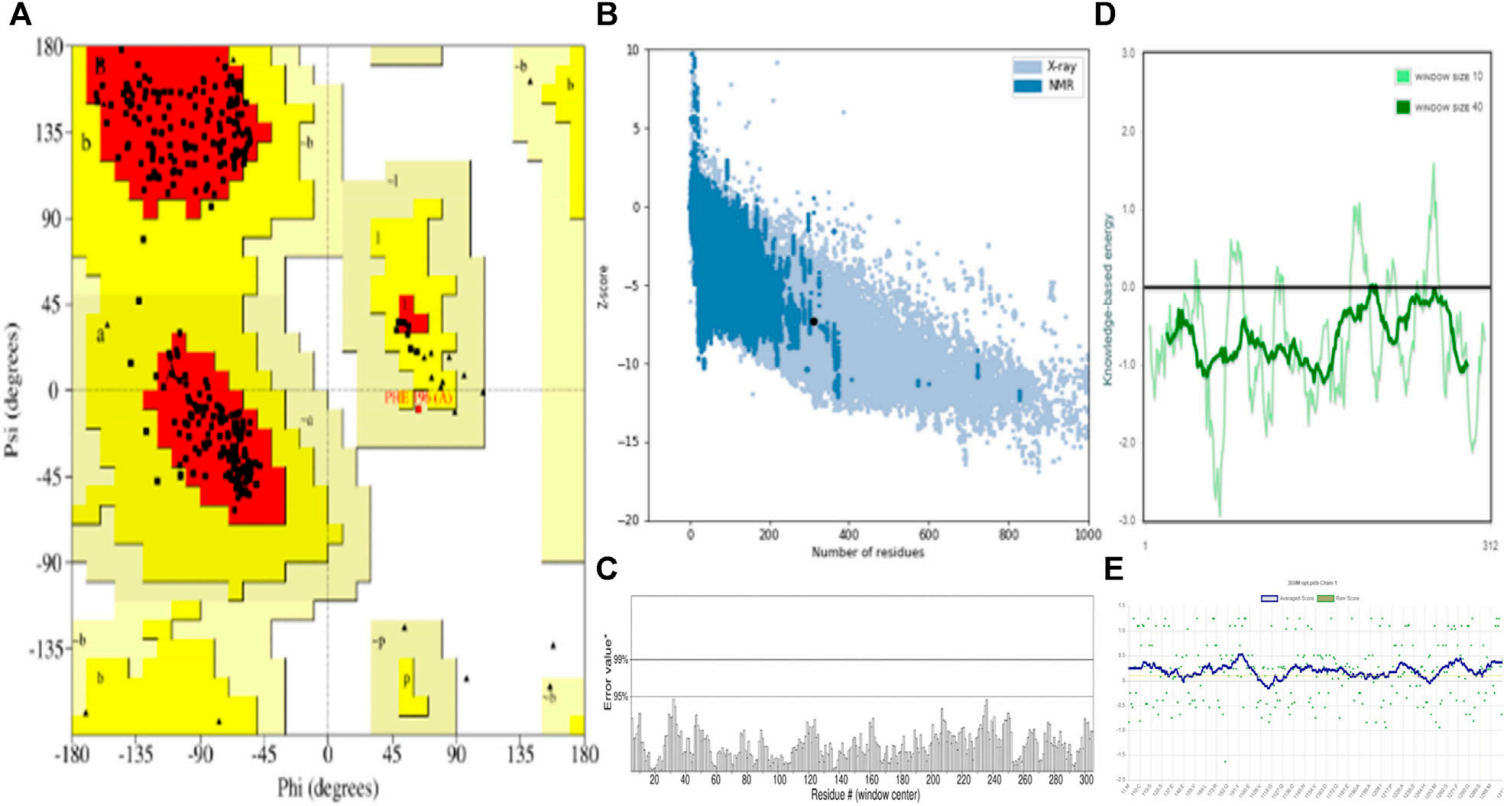
Validation and quality evaluation of protein (PDB: 3E0M) by using **(A)** Rama-chandran plot analysis using the Procheck tool, **(B)** Z-Score prediction from the ProSA-web Server, **(C)** ERRAT, **(D)** Local model quality and **(E)** Varify3D plot showing amino acids in favored regions.

Molecular docking is a vital computational approach for evaluating the binding affinity between a ligand and a protein or receptor. This method offers valuable insights into how compounds interact with a protein’s active site residues and influence cellular processes. In our study, we performed molecular docking experiments involving naphthoquinone derivatives (**1–13**) and three reference drugs—ciprofloxacin, ofloxacin, and vancomycin—against four specific proteins: MSR (3E0M), MERS-CoV (4RNA), EFGR (4UV7_L), and MDD (4DPT), as detailed in the table. To ensure the quality of the protein structure (PDB: 3E0M), we conducted various assessments, including PROCHECK, ERRAT, Verify3D, ProSA-web server, and ProSA-web energy server, as illustrated in Figure 4. These evaluations provided valuable insights into the protein’s structure. The structure met high-quality standards, as evidenced by the Ramachandran plot, overall quality factor, Verify3D score, and ProSA-web energy plot. In summary, the results confirm the validation and excellent quality of the protein structure, providing a solid foundation for our molecular docking Studies.

Table 2 illustrates the docking results, indicating that compounds **1–13** demonstrate notably higher binding affinities with the 3E0M protein compared to the other proteins in the study. A recent study reported that compound **11** exhibited a lower minimum inhibitory concentration *in vitro*. Our findings further highlight that compound **11** displays the highest binding affinity among all tested proteins, mainly showing strong affinities for Peptide methionine sulfoxide reductase msrA/msrB (PDB: 3E0M) −7.9 kcal mol^−1^, aligning with our desired outcome. Compound **11** also exhibits binding affinity, Mevalonate diphosphate decarboxylase (PDB: 4DPT) −7 kcal mol^−1^, The middle east respiratory syndrome coronavirus (MERS-CoV) papain like protease (PLpro) (PDB: 4RNA) −6.2 kcal mol^−1^, and Epidermal Growth Factor Receptor (PDB: 4UV7) −6.5 kcal mol^−1^. Table 2 in the SI shows that in the evaluation of MSR (PDB: 3E0M), all reference drugs demonstrated lower docking values ranging from −7.3 to −8 kcal mol^−1^, in contrast to compounds **9** (−7.6 kcal mol^−1^), **13** (−7.5 kcal mol^−1^), and **11** (−7.9 kcal mol^−1^). Additionally, the results indicate that most of the examined docking values for our compounds surpass those of the reference drugs used in antibacterial and cancer treatment. To summarize, compounds **11** and ofloxacin (−7.8 kcal mol^−1^) exhibit strong binding affinities for all targeted proteins, while some compounds, such as **7** (−5.5 kcal mol^−1^) and **8** (−5.8 kcal mol^−1^), **12** (−6.2 kcal mol^−1^), exhibit lower binding affinities, possibly attributed to differences in their chemical structures.

**TABLE 2:** Molecular docking simulation results for naphthoquinone derivatives (**1–13**) against four target proteins.

Ligand	3E0M	4DPT	4UV7_L	4RNA
**1**	−5.4	−5.5	−5.6	−5.2
**2**	−5.3	−5.7	−4.9	−5.0
**3**	−4.4	−5.2	−4.4	−4.5
**4**	−4.3	−5.8	−5.0	−4.8
**5**	−5.4	−5.6	−5.3	−5.2
**6**	−5.2	−6.0	−5.6	−4.9
**7**	−5.5	−5.7	−5.5	−5.1
**8**	−5.8	−6.4	−5.4	−5.6
**9**	−7.6	−6.9	−6.4	−6.1
**10**	−6.2	−8.4	−6.7	−5.9
**11**	−7.9	−7.2	−6.5	−6.2
**12**	−6.2	−7.4	−5.0	−6.2
**13**	−7.5	−7.4	−7.3	−6.7
Ciprofloxacin	−7.3	−5.8	−6.1	−5.5
Ofloxacin	−7.8	−7.1	−6.3	−5.9
Vancomycin	−8.0	−6.3	−5.9	−6.1

When treated with naphthoquinone derivatives, the functions of key enzymes like MERS-CoV papain-like protease (PLpro), Epidermal Growth Factor Receptor (EGFR), and Mevalonate Diphosphate Decarboxylase are potentially altered, impacting their roles in disease processes. Naphthoquinone derivatives, known for their broad biological activities, could inhibit MERS-CoV PLpro, disrupting the virus’s replication and ability to evade the immune system. This inhibition could be crucial in developing new antiviral strategies against MERS-CoV. In the case of EGFR, which plays a significant role in cancer cell proliferation, naphthoquinone derivatives might act as inhibitors, blocking EGFR’s signaling pathways that lead to tumor growth and metastasis. This suggests a promising avenue for cancer therapy, particularly in EGFR-driven cancers. Lastly, targeting Mevalonate Diphosphate Decarboxylase, a key enzyme in cholesterol biosynthesis, with these derivatives could reduce cholesterol synthesis. This action holds potential in treating disorders related to cholesterol metabolism, such as cardiovascular diseases. Thus, applying naphthoquinone derivatives to these enzymes offers a multifaceted therapeutic approach, spanning antiviral, anticancer, and cholesterol-lowering effects, demonstrating the versatility of these compounds in disease treatment and management.

**FIGURE 5:**
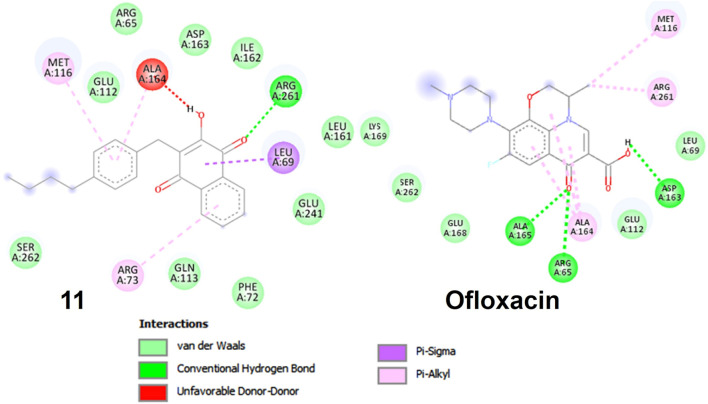
Molecular docking poses: Ligand-protein interaction for 2D diagram of compounds **11** and ofloxacin in 3E0M.

**FIGURE 6:**
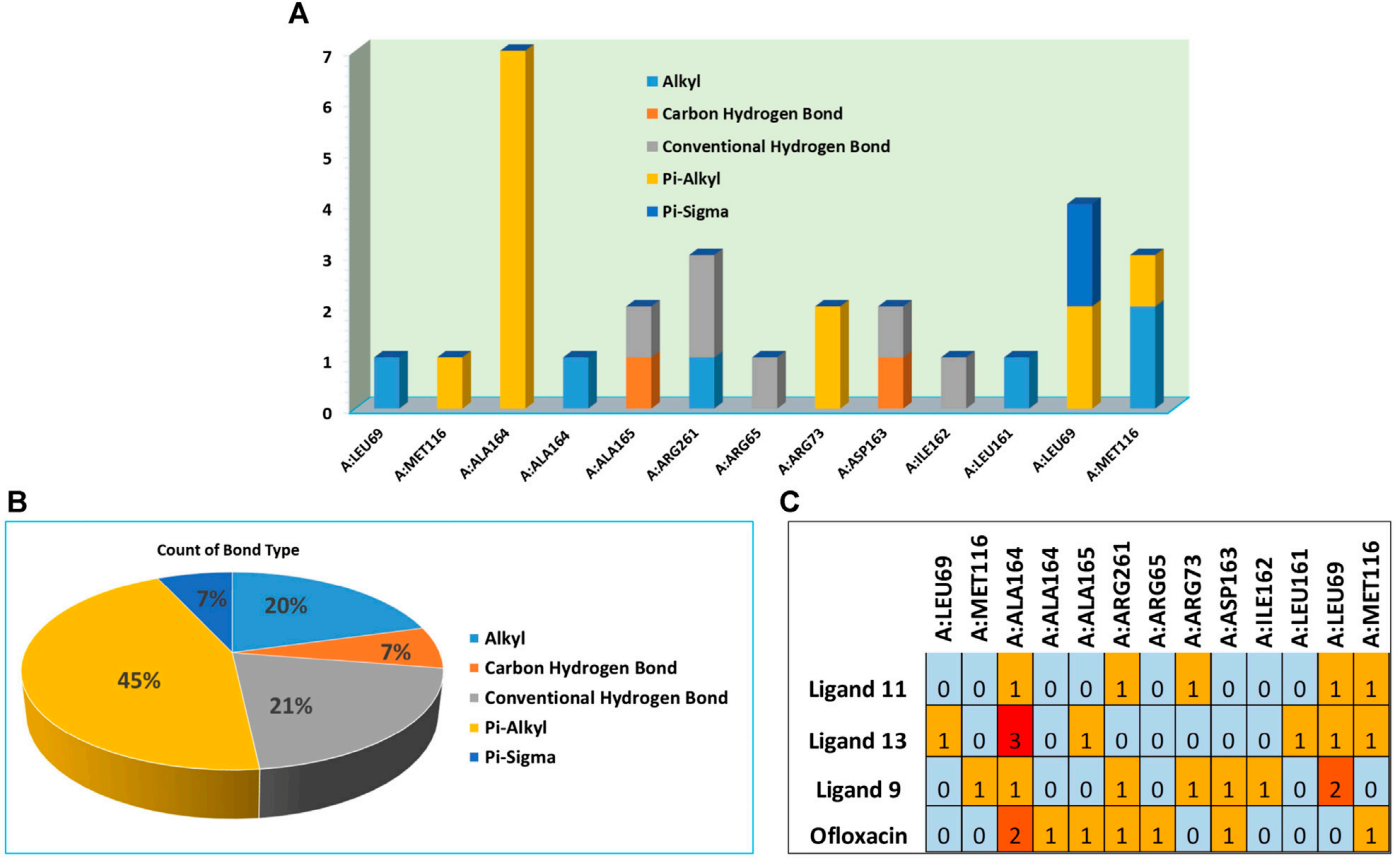
**(A)** Residues in interaction within 3E0M, **(B)** the distribution of non-covalent interactions, and **(C)** a map illustrating the interaction between residues in 3E0M-naphthoquinone derivatives **9**, **11**, **13**, and the reference drug of ofloxacin complex.

In compound **11** and its interaction with the 3E0M protein, four distinct interactions have been identified, as outlined in Table 3 and Figures 5, 6 (Supplementary Figures S16–S18). These interactions include four hydrophobic interactions, specifically Pi-Alkyl interactions with MET A:116 and ARG A:73 on the residue and Pi-sigma interactions with LEU A:69. Additionally, one interaction is classified as a conventional hydrogen bond at the ALA A:165 residue. Compound 13’s protein-ligand interaction analysis with 3E0M revealed one conventional hydrogen interaction at the VAL A:465 residue, one halogen interaction with GLY A:367, and four Pi-Alkyl interactions involving residues ALA A:556, ARG A:415, ALA A:366, and VAL A:606. Compound **9** exhibited a binding affinity for 3E0M (−7.4 kcal/mol) with seven interactions in total, as shown in Figure 5 and Supplementary Figure S16. These interactions included two conventional hydrogen bonds (ARG A:261, ILE A:162), a Carbon Hydrogen Bond with ASP A:163, and Pi-Alkyl interactions involving MET A:116, ALA A:164, and ARG A:261.

**TABLE 3:** Ligand-protein interaction for protein (PDB: 3E0M) with compounds **9**, **11**, **13**, and ofloxacin (D2).

Drug	Amino acid residue	bond category	distance ( Å )	type of interaction
**9**	LEU A:69	Hydrophobic	2.67	Pi-Sigma
	MET A:116	Hydrophobic	3.93	Pi-Alkyl
	ALA A:164	Hydrophobic	4.62	Pi-Alkyl
	ARG A:73	Hydrophobic	5.29	Pi-Alkyl
	ARG A:261	HB	2.15	Hydrogen Bond (HB)
	ILE A:162	HB	2.38	Hydrogen Bond (HB)
	ASP A:163	HB	2.44	Conventional HB
**11**	LEU A:69	Hydrophobic	2.72	Pi-Sigma
	MET A:116	Hydrophobic	4.73	Pi-Alkyl
	ARG A:73	Hydrophobic	5.08	Pi-Alkyl
	ARG A:261	HB	2.15	Conventional HB
**13**	ALA A:164	Hydrophobic	4.15	Pi-Alkyl
	LEU A:161	Hydrophobic	4.35	Pi-Alkyl
	LEU A:69	Hydrophobic	4.48	Pi-Alkyl
	MET A:116	Hydrophobic	5.08	Pi-Alkyl
	ALA A:165	HB	2.54	Conventional HB
D2	MET A:116	Hydrophobic	5.12	Pi-Alkyl
	ARG A:261	Hydrophobic	4.29	Pi-Alkyl
	ALA A:164	Hydrophobic	4.76	Pi-Alkyl
	ALA A:165	HB	2.69	Conventional HB
	ARG A:65	HB	2.24	Conventional HB
	ASP A:163	HB		

**FIGURE 7:**
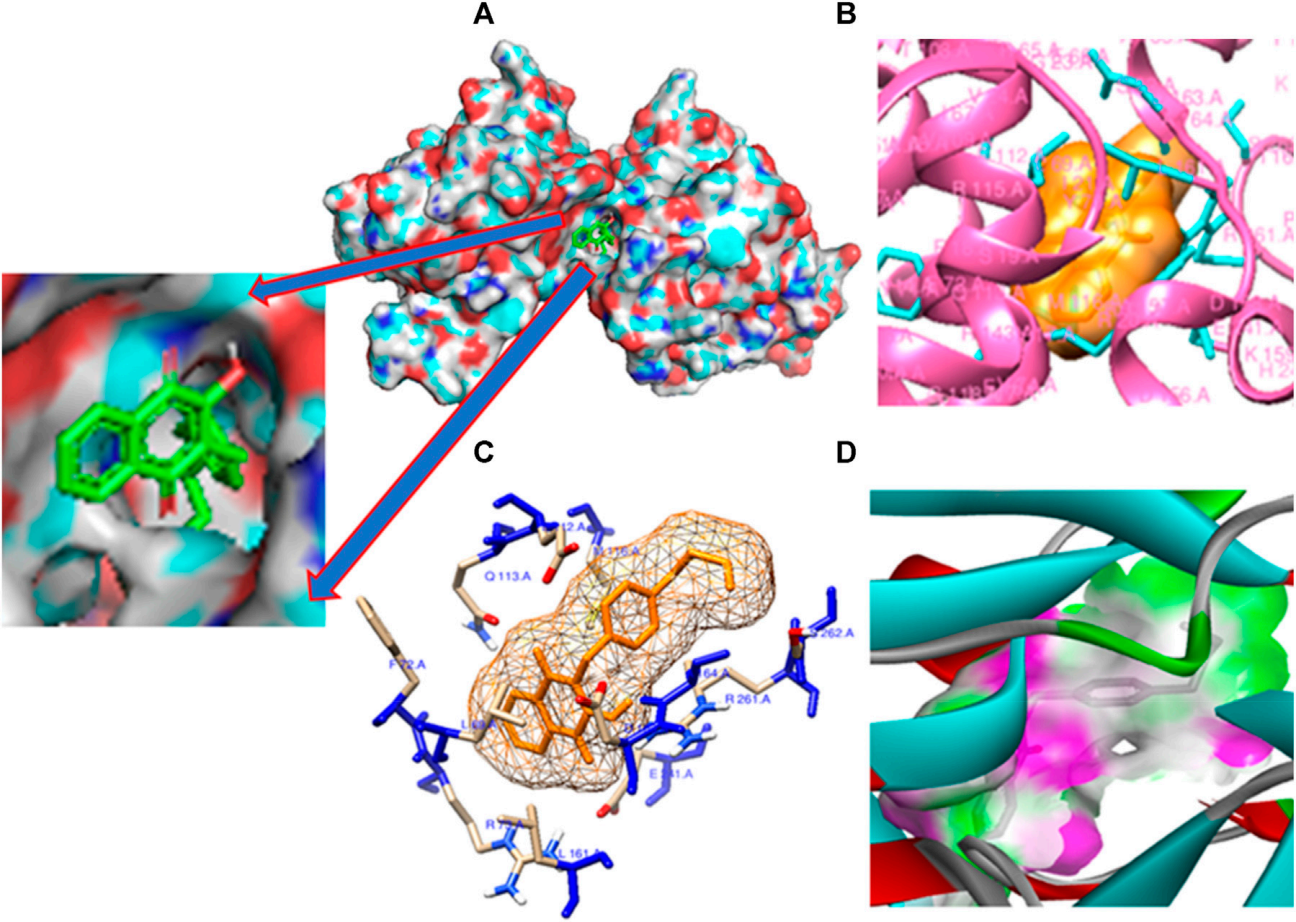
Molecular docking poses: **(A)** Protein ligand binding cavity; **(B)** Ligand placement in protein pocket; **(C)** Active site positioning; and **(D)** Visualization of hydrogen bonding in solid for the modeled 3E0M protein with ligand **11**.

The pivotal finding of this study highlights compound **11** as an exceptionally promising candidate for drug development due to its profound interaction with the 3E0M protein (Figure 7). Compound **11** demonstrates superior binding affinity to the 3E0M protein compared to other compounds and reference drugs like ofloxacin and ciprofloxacin, engaging four distinct amino acid residues. This comprehensive and specific binding profile underscores the remarkable affinity of compound **11** for the target protein. Based on the molecular docking and interaction analyses, compound **11** exhibits heightened binding affinity and forms a stronger interaction with the target protein compared to other compounds. This indicates its potential as a more effective inhibitor of proteins 3E0M and 4DPT, which are relevant in anti-cancer treatment and antibacterial activities. Consequently, this *in silico* study suggests that compound **11** holds substantial promise as an inhibitor of bacterial cell membrane proteins.

### 3.3 Analysis of physicochemical and pharmacokinetic properties

Two critical criteria, Lipinski and Veber, play a pivotal role in evaluating the physicochemical attributes of compounds. The investigation of these properties is essential to determine whether Naphthoquinone derivatives (**1–13**) conform to Lipinski and Veber guidelines. Lipinski’s criteria, crucial for preparing a compound for oral use, include five key conditions: a. Molecular weight (MW) of 500 g/mol or less b. Partition coefficient (log P) of 5 or less c. No more than 5 hydrogen bond donors (HBD) d. A maximum of 10 hydrogen bond acceptors (HBA) e. Topological polar surface area (TPSA) within 140 Å^2^ (Veber et al., 2002; Lipinski, 2004). Veber’s guidelines add two more requirements for effective bioavailability: a. Fewer than 10 rotatable bonds (nrotb) b. TPSA not exceeding 140 Å^2^, aligning with Lipinski’s rules. We utilized SwissADME to evaluate the compliance of compounds **1–13** with these criteria, particularly concerning their potential biological activity. Our analysis revealed that all compounds (**1–13)** adhered to Lipinski and Veber’s specific requirements. Furthermore, with a drug-like (bioavailability) score of 1, all compounds (**1–13**) were confirmed to meet the criteria for developing new medications, supported by strong theoretical evidence, with high agreement (less than 6) for drug compounds having a molecular weight below 500 g/mol, except vancomycin (MW = 1,449.2 g/mol), MLOGP of 4.15, and Log S (ESOL) values within the specified guidelines. Medicinal chemistry analysis, including the assessment of PAINS #alerts and Brenk#alerts, was also conducted using SwissADME for the studied structures. Among the **13** synthesized naphthoquinone derivatives (**1–13**), compound **11** exhibited the highest negative binding affinity, surpassing −7.9 kcal mol^−1^, as shown in Table 2. Notably, compound **11** displayed favorable drug-likeness characteristics, with no PAINS #alerts and 0 Brenk #alert among the derivatives. Additionally, *in silico* ADMET profiling of compound **11**, which demonstrated the highest negative binding affinity in molecular docking, met the requirements, indicating good drug-like properties (refer to Table 4). Consequently, based on their promising physicochemical properties, these compounds hold potential for further development as novel medications.

**TABLE 4:** Lipinski’s rule of five and Veber’s rule prediction for drug-likeness of compounds (**1–13**).

Ligand	MW	mLogP	nHBD	nHBA	Lipinski’s violations	Veber’s violations	TPSA	nRot	PAINS #alerts	Brenk #alerts
**Lipinski^a^**	≤500	≤5	≤5	≤10	–	–	–	–	–	–
**Veber^b^**	–	–	–	–	–	–	≤140	≤10	–	–
**1**	172.22	2.53	0	1	0	0	17.07	2	0	0
**2**	200.28	2.97	0	1	0	0	17.07	4	0	0
**3**	106.12	1.36	0	1	0	0	17.07	1	0	0
**4**	134.18	1.91	0	1	0	0	17.07	2	0	0
**5**	162.23	2.32	0	1	0	0	17.07	4	0	0
**6**	190.28	2.75	0	1	0	0	17.07	6	0	0
**7**	218.33	3.21	0	1	0	0	17.07	8	0	0
**8**	158.15	1.44	0	2	0	0	34.14	0	0	0
**9**	264.28	2.25	1	3	0	0	54.37	2	0	1
**10**	292.33	2.71	1	3	0	0	54.37	3	0	0
**11**	320.38	3.11	1	3	0	0	54.37	5	0	0
**12**	348.43	3.58	1	3	0	0	54.37	7	0	0
**13**	376.49	4.1	1	3	0	0	54.37	9	0	0
Ciprofloxacin	331.34	2.24	2	7	0	0	72.9	3	0	0
Ofloxacin	361.37	2.49	1	8	0	0	73.3	2	0	0
Vancomycin	1,449.2	0.11	19	25	0	0	531	19	0	0

Note.

^a^ Lipinski reference values.

^b^ Veber reference values; MW, molecular weight; LogP, lipophilicity (O/W); HBD, number of hydrogen bond donors; HBA, number of hydrogen bond acceptors; nroth, Number of rotatable bonds; TPSA, topological polar surface area ( ${Å}^{2}$ ).

In our study, we utilized Molinspiration Chemoinformatics to assess the drug-like properties of naphthoquinone derivatives (**1–13**), targeting diverse biological receptors such as G protein-coupled receptors (GPCRs), ion channel modulators (ICMs), kinase inhibitors (KIs), nuclear receptor ligands (NRLs), protease inhibitors (PIs), and enzyme inhibitors (EIs), as detailed in Table 5. Notably, compounds **9** and **11** exhibited GPCR values of −0.19 and −0.16, respectively, while olioxacin demonstrated a GPCR value of 0.23. The lower GPCR value indicates better drug-like properties. Efficient development of novel drugs requires exploration of pharmacokinetic properties, encompassing absorption, distribution, metabolism, excretion, and toxicity (ADMET). In our investigation, we employed free tools such as SwissADME (http://www.swissadme.ch/index.php) (Daina et al., 2017) and AdmetSAR (http://lmmd.ecust.edu.cn/admetsar2/) (Yang et al., 2019) to evaluate the ADMET properties of all 13 active compounds (**1–13**). We assessed seven essential ADMET characteristics, including cytochrome P450 enzymes (CYP3A4 and CYP2C19) inhibition, hERG inhibition, blood-brain barrier (BBB) penetration, human intestinal absorption (HIA), and synthetic accessibility (SA) score, presented in Table 6. It is worth noting that BBB penetration is particularly crucial for drugs targeting the central nervous system (CNS) but may not be as critical for drugs with minimal CNS impact. BBB penetration is categorized into three ranges: high (>2), medium (2–0.1), and low (<0.1) adsorption (Veber et al., 2002). The results indicate that all compounds (**1–13**) meet ADMET standards for drug-likeness (bioavailability) and hold the potential for developing novel drugs.

**TABLE 5:** Drug-likeness assessment of naphthoquinone derivatives (**1–13**) by molinspiration tool.

Ligand	GPCR	ICM	KI	NRL	PI	EI
**1**	−0.14	0.44	0.31	0.04	−0.49	0.24
**2**	0.03	0.47	−0.13	0.20	−0.27	0.33
**3**	−3.43	−2.75	−3.38	−3.13	−3.58	−2.99
**4**	−1.29	−0.50	−1.45	−1.06	−1.61	−0.75
**5**	−0.79	−0.26	−1.07	−0.65	−1.09	−0.40
**6**	−0.53	−0.13	0.78	−0.39	−0.79	−0.22
**7**	−0.34	−0.05	−0.54	−0.20	−0.56	−0.10
**8**	−0.94	−0.46	−0.77	−1.00	−1.10	−0.34
**9**	−0.19	−0.15	−0.03	0.22	−0.13	0.23
**10**	−0.04	−0.13	0.02	0.29	−0.04	0.22
**11**	−0.16	−0.09	0.04	0.36	0.09	0.26
**12**	0.08	−0.08	0.04	0.34	0.11	0.25
**13**	0.07	−0.08	0.04	0.31	0.20	0.23
Ciprofloxacin	0.12	−0.04	−0.07	−0.19	0.20	0.28
Ofloxacin	0.23	−0.14	−0.06	−0.13	−0.26	0.35
Vancomycin	−3.08	0.20	−0.09	0.20	−0.3	0.44

**TABLE 6:** *In silico* prediction of selected ADMET parameters for all compounds (**1–13**).

Ligand	^a^HIA	^a^BBB	^a^PPB	^a^CYP3A4 inhibition	^a^CYP2C19 inhibition	^a^hERG_pIC50	SA score
**1**	+(0.9957)	+(1.000)	1.078	-(0.6912)	-(0.8591)	-(0.5888)	2.44
**2**	+(0.9967)	+(1.000)	1.138	-(0.6453)	-(0.8494)	-(0.4095)	2.74
**3**	+(0.9957)	+(0.975)	0.725	-(0.8575)	-(0.9492)	-(0.8919)	1.00
**4**	+(0.9973)	+(0.975)	0.659	-(0.7795)	-(0.9297)	-(0.7418)	1.00
**5**	+(1.0000)	+(1.000)	0.964	-(0.9791)	-(0.8689)	-(0.6226)	1.02
**6**	+(1.0000)	+(1.000)	0.479	-(0.8967)	-(0.9025)	-(0.4056)	2.30
**7**	+(1.0000)	+(1.000)	0.675	-(0.9778)	-(0.9082)	+(0.7380)	2.24
**8**	+(1.0000)	-(0.550)	0.564	-(0.8310)	+(0.7633)	-(0.8417)	2.82
**9**	+(0.9969)	-(0.675)	0.998	-(0.9104)	-(0.9025)	-(0.8161)	3.00
**10**	+(1.0000)	-(0.600)	0.877	-(0.9286)	-(0.7171)	-(0.4056)	3.21
**11**	+(1.0000)	+(0.625)	1.083	-(0.8885)	-(0.7108)	-(0.6441)	3.97
**12**	+(1.0000)	-(0.625)	1.094	-(0.8502)	-(0.7252)	-(0.4211)	3.68
**13**	+(1.0000)	-(0.625)	1.089	-(0.8502)	-(0.7252)	-(0.7320)	2.51
Ciprofloxacin	+(0.9841)	+(0.714)	0.488	-(0.8309)	-(0.9025)	-(0.8225)	3.63
Ofloxacin	+(0.9777)	+(1.000)	0.376	-(0.8309)	-(0.9025)	-(0.8179)	2.44
Vancomycin	+(0.698)	-(0.914)	0.539	-(0.8309)	-(0.9025)	+(0.7217)	2.74

Note: ^a^HIA, Human Intestinal Absorption (%); BBB, Blood-Brain Barrier penetration; PPB, plasma protein binding; CYP3A4, Cytochrome P4503A4; CYP2C19, Cytochrome P4502C19; hERG, human ether-a-go-go-related gene, hERG, inhibition potential (pIC50), the potential risk for inhibitors ranges 5.5–6. The values are using admetSAR.bThe values are using swissADME. D1:Ciproflaxacin and D2:Ofloxacin and D3:vancomycin.

Our findings reveal that, with the exceptions of compounds **8** and **10**, most compounds exhibited moderate blood-brain barrier (BBB) penetration values, falling within the range of 0.625–1. Ciprofloxacin and ofloxacin displayed medium absorption values of 0.714 and 1, respectively, while vancomycin exhibited low absorption at −0.914. Moreover, the majority of naphthoquinone derivatives demonstrated favorable synthetic accessibility, ranging from 1 to 3.68, comparable to standard drugs. Compound **11**, with a synthetic accessibility score of 3.97, and ofloxacin, with a score of 2.44, exemplified these characteristics. Our predictions regarding the potential risk of hERG activity inhibitors, with values ranging from −0.4 to −0.82 (Shadrack and Ndesendo, 2017), indicated that compound **11** achieved the desired hERG pIC50 value of −0.6441. In contrast, all other compounds remained within the reference range. These results affirm that all compounds (**1–13**) adhere to ADMET standards, signifying drug-likeness (bioavailability), and underscore their potential for novel drug development. Specifically, compound **11** exhibits favorable properties across all ADMET parameters, including moderate BBB penetration, high intestinal absorption, and a low risk of hERG inhibition. These characteristics align with the desired profile for a potential therapeutic agent.

### 3.4 Molecular dynamics simulation

We conducted molecular dynamics (MD) simulations spanning 20 ns for compounds **9**, **11**, **13**, and ofloxacin in conjunction with the MSR protein (PDB: 3E0M). Previous molecular docking investigations revealed robust binding affinities of −7.6, −7.9, −7.5, and −7.8 kcal/mol for compounds **9**, **11**, **13**, and ofloxacin, respectively, with the 3E0M protein, indicating their potential as promising drug candidates. The MD simulations aimed to assess the stability and interactions within the protein-ligand complex throughout the 20 ns trajectory. Various parameters, including RMSD (root-mean-square deviation), RMSF (root-mean-square fluctuation), Rg (radius of gyration) values, potential energies, temperature, hydrogen bonding, and principal component analysis (PCA), were employed for the simulation analysis. Additionally, the complex underwent simulations at four distinct temperatures (300, 305, 310, and 320 K) to explore its structural alterations under varying thermal conditions. This approach provided insights into the stability and adaptability of the protein-ligand complex under different physiological conditions.

**FIGURE 8:**
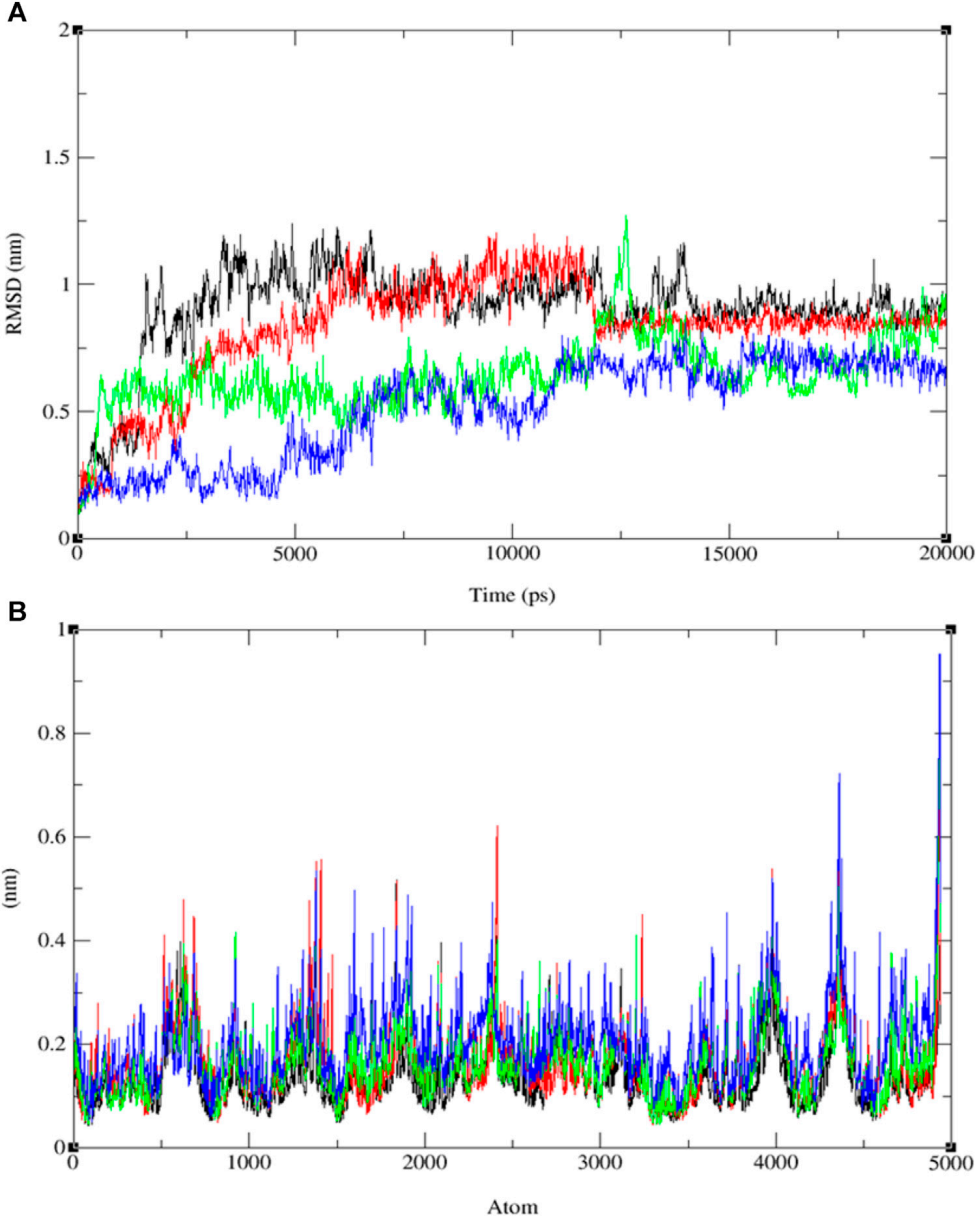
The progression of the root mean square deviation (RMSD) **(A)** and the root mean square fluctuation (RMSF) **(B)** for the combined docked complex involving the protein (PDB: 3E0M) and ligands **9** (black line), **11** (blue line), **13** (red line), and ofloxacin (green line) during the 20 ns MD simulation.

The analysis of RMSD values obtained from the MD simulations provides insights into the stability and conformational dynamics of ligand-protein complexes (refer to Figure 8A and Supplementary Figures S19–S21). Notably, the complex involving compound **11** and protein 3E0M displayed the highest conformational stability, with RMSD values ranging from 0.2 to 0.55 nm (blue line), indicating minimal deviation from the initial structure. The complex of compound **13** and 3E0M exhibited the second-highest stability, showing RMSD values from 0.3 to 0.6 nm, although it demonstrated larger fluctuations, reaching up to 0.7 nm. In contrast, the complexes involving ofloxacin and 3E0M, as well as compound **9** with 3E0M, displayed lower stability, evident from increased scattering in their RMSD plots, with values spanning from 0.2 to 0.7 nm and 0.1 to 0.8 nm, respectively.

In addition to RMSD, root-mean-square fluctuation (RMSF) assessment was utilized to gauge the flexibility of individual amino acid residues within the protein-ligand complexes throughout the MD simulations. RMSF values offer insights into the dynamic behavior of specific amino acids, indicating their level of flexibility or rigidity. Analyzing the RMSF values for each compound interacting with protein 3E0M revealed variations in residual flexibility among the complexes. The observed fluctuations ranged approximately from 0.04 to 0.13 nm for ligand **11**, 0.03–0.9 nm for the target protein 3E0M, and 0.05–0.9 nm for the protein-ligand complex (see Figure 8B and Supplementary Figures S22–S24). These values suggest that a majority of amino acid residues in the complex exhibited fluctuations. Notably, regions directly interacting with ligand **11** exhibited low fluctuations, while other regions containing amino acids without direct interactions displayed more significant fluctuations. The complex involving protein 3E0M and ligand **11** demonstrated the highest RMSF values, indicating greater flexibility compared to other complexes (refer to Figure 8B and Supplementary Figures S22–S24). This implies that ligand **11** may induce a more dynamic conformation in the protein compared to the other ligands. The complex of protein 3E0M with ligand **13** also displayed moderate flexibility, while the complexes of protein 3E0M with ligand **9** and ofloxacin-3E0M exhibited relatively higher rigidity.

**FIGURE 9:**
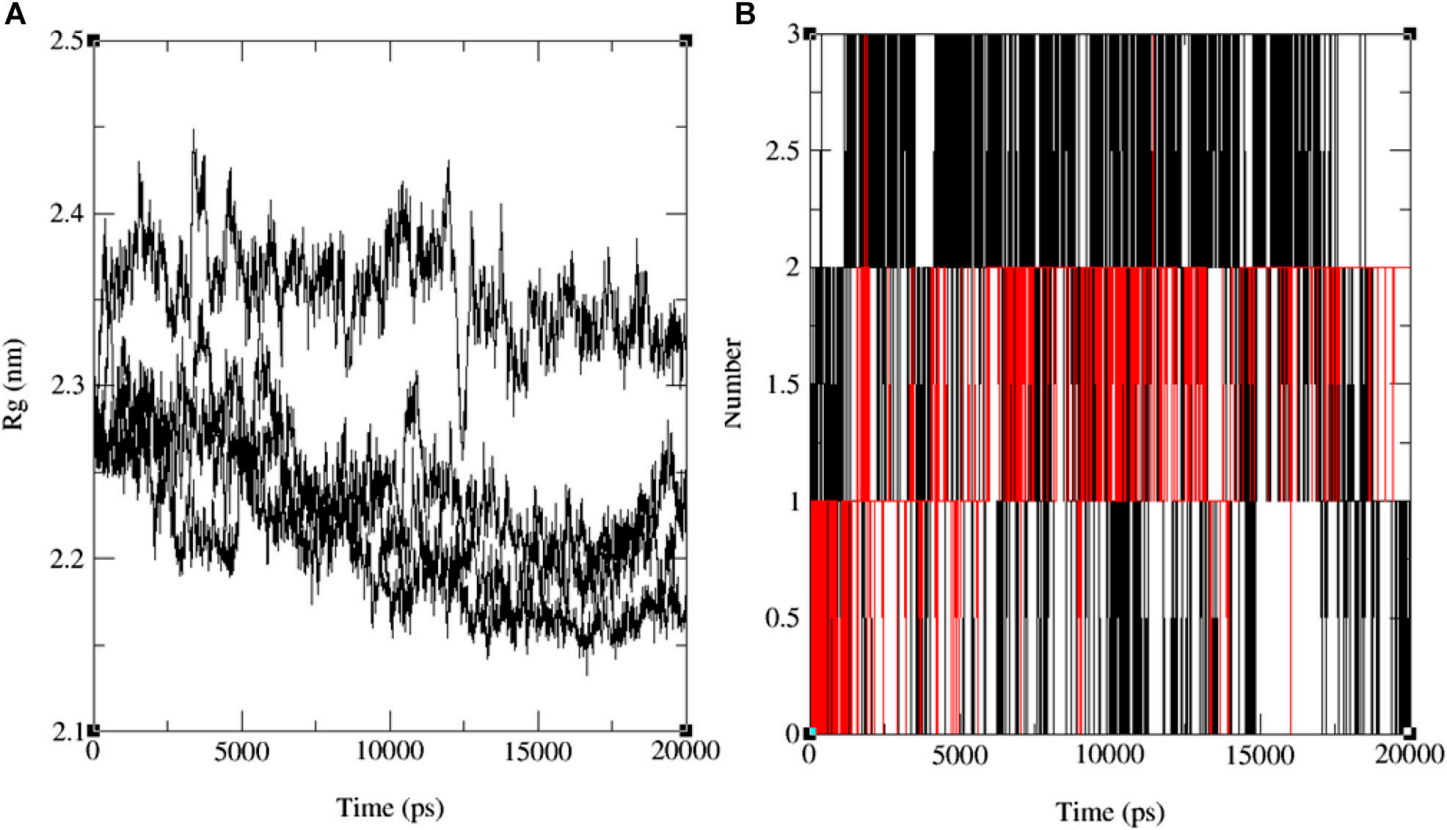
The progression of the radius of gyration (Rg) **(A)** and the hydrogen bond count (HBs) **(B)** for the combined docked complex involving the protein (PDB: 3E0M) and ligand **11** during the 20 ns MD simulation.

The variations in RMSF values suggest diverse flexibility within the protein-ligand complexes, potentially influencing their biological functions. The analysis of RMSF provides valuable insights into the structural dynamics of protein-ligand interactions, complementing the information derived from RMSD analysis. These findings indicate the stability of both the ligand and the protein throughout the simulation, with minor deviations in atomic positions. Notably, the observed fluctuations in specific ligand atoms may carry significance, potentially related to particular functional or interactional aspects of the protein. However, further investigation is necessary to ascertain these fluctuations’ precise implications and potential impacts on the biological activities associated with 3E0M. In this computational study, it has been assessed that the radius of gyration (Rg) is a parameter used to characterize the size and shape of macromolecules like protein-ligand complexes. The Rg value determined for the rotein-ligand complex ranged from 2.12 to 2.45 nm, as depicted in Figure 9A (see Supplementary Figures S25–S28).

Notably, all findings indicate that including the ligand did not induce substantial alterations in the overall size or shape of the protein. This outcome aligns with the notion that the binding of the ligand to the protein occurred in a manner that preserved the protein’s tertiary structure without causing significant disruptions. Furthermore, the comparison in Rg values between the protein and the protein-ligand complex suggests that the binding event did not bring about notable structural modifications within the protein. Nevertheless, it is crucial to acknowledge that while Rg values provide insights into a protein’s general size and shape, they may not unveil the precise molecular-level structural alterations that have transpired. Further experiments, such as X-ray crystallography or NMR spectroscopy, may be indispensable for a more comprehensive understanding of the molecular interactions between the protein and the ligand. The results of the MD simulations emphasize the significance of the number of hydrogen bonds (HB) formed between the protein and the ligand—a critical factor in sustaining the stability of the protein-ligand system. These findings are depicted in the time-dependent assessment of intermolecular hydrogen bonds during the 20 ns simulation, as shown in Figure 9B and Supplementary Figure S29. The simulation conducted at the standard body temperature exhibited the most consistent HB profile, with the number of hydrogen bonds fluctuating between zero and four throughout the entire 20 ns simulation period. Notably, between the 5 ns and 15 ns timeframe (Figure 9B), ligand **11** demonstrated the highest occurrence of hydrogen bond formations with the residues of the target protein (PDB: 3E0M). Initially, the analysis of interactions within the docked complex did not clearly indicate the presence of hydrogen bonds, as seen in Figure 8B. However, the subsequent 20 ns MD simulation revealed the emergence of hydrogen bond interactions for ligands **9**, **11**, **13**, and the reference ligand (ofloxacin).

**FIGURE 10:**
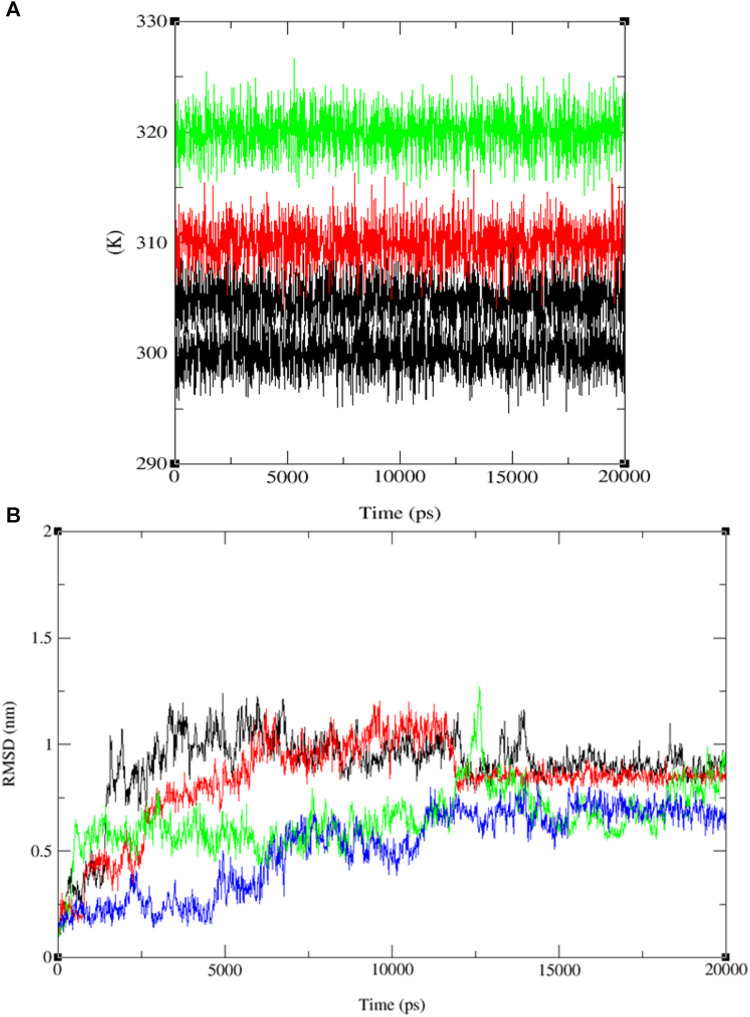
Temperature (K) *versus* Time (ps) plots for **(A)** the combined system at four different temperature conditions (300, 305, 310, and 320 K), and **(B)** RMSD for the combined docked complex involving the protein (PDB: 3E0M) and ligand **11** (300 K: black line, 305 K: red line, 310 K: blue line, 320 K: green line) throughout the 20 ns MD simulation.

Upon comparing the outcomes of simulations involving compounds **9**, **11**, **13**, and ofloxacin with the MSR protein (PDB: 3E0M), it is evident that compound **11** establishes a more robust interaction with 3E0M. This suggests its potential as an inhibitor of the MSR protein, which holds significance due to its crucial role in cancer and bacterial inflammation. Previous *in vitro* and *in vivo* experiments have underscored the promise of compound **11** as a candidate for an anti-cancer and antibacterial drug. This is attributed to its potent anti-mycobacterial activity, low cytotoxicity, and unique lipophilic vehicle feature. The complex comprising compound **9** and MSR showed HOMO-LUMO combined results slightly lower than ofloxacin, which exhibited the most pronounced negative binding affinity and met all criteria for consideration as a drug candidate, as substantiated by the molecular simulation results at 310 K. To further corroborate our identification of compound **11** as a promising anticancer and antibacterial drug candidate, additional molecular dynamics simulations were performed at three different temperatures: 300, 305, 310, and 320 K for the complex of ligand **11** with 3E0M, as depicted in Figure 10A.

The RMSD analysis of the ligand **11**–MSR protein complex revealed its optimum stability at 310 K, with minimal deviation and a peak value of 0.8 nm. At 305 K, the complex exhibited the second-highest stability, with RMSD values fluctuating between 0.1 and 0.4 nm and peaking at 0.5 nm. In contrast, the complex demonstrated reduced stability at 320 K and 300 K, manifesting more pronounced deviations. At these temperatures, the RMSD values ranged from 0.2 to 0.4 nm and 0.1 to 0.7 nm, respectively (see Figure 10B and Supplementary Figures S30–S44).

**FIGURE 11:**
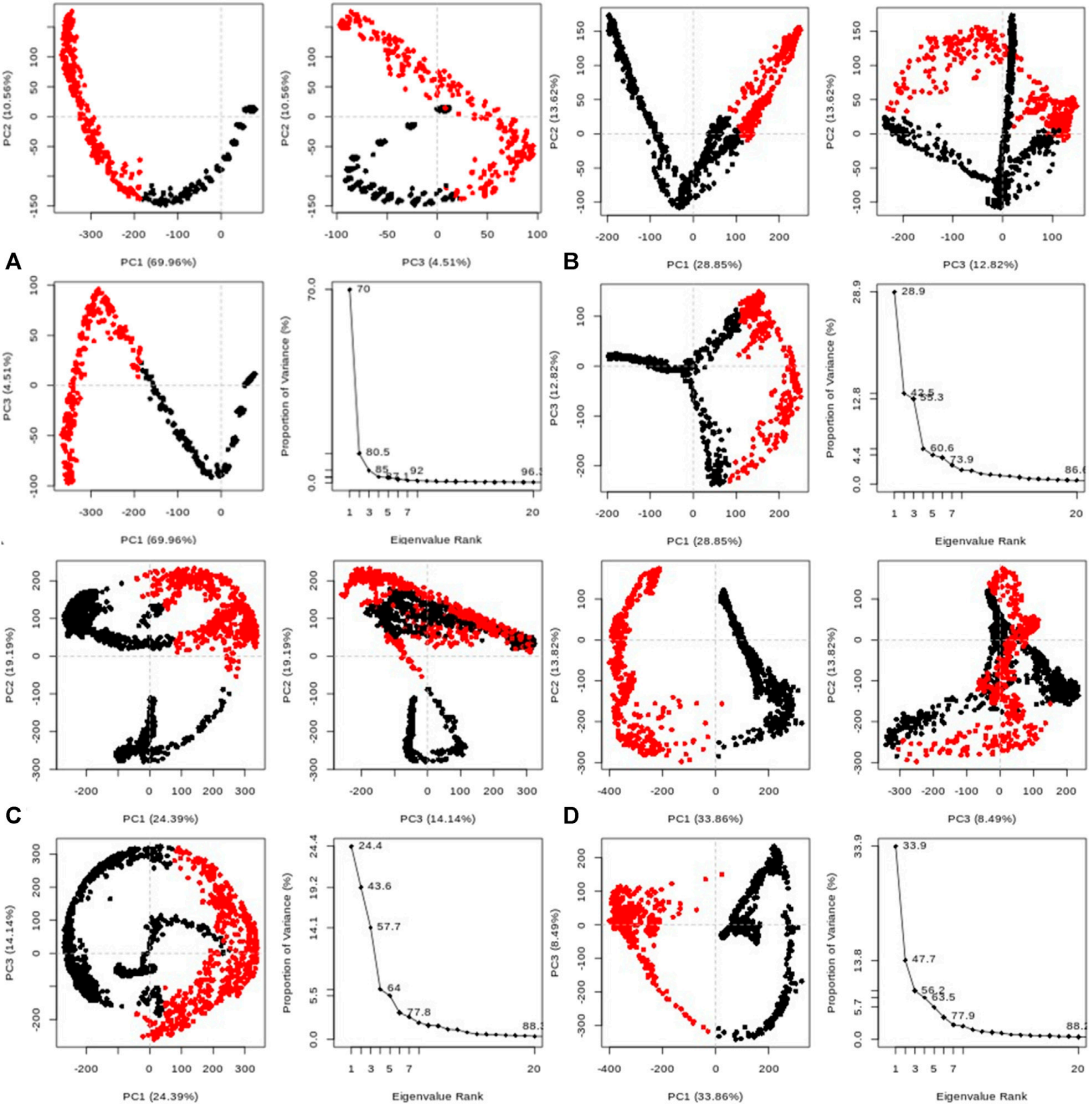
Principal component analysis (PCA) of MD trajectories for the target protein (PDB: 3E0M) and ligand **11** complex at **(A)** 300 K, **(B)** 305 K, **(C)** 310 K, and **(D)** 320 K (Intermediate states are marked by white dots, energetically unstable conformations are represented by blue dots with scattering, and stable conformation states are denoted by red dots).

Furthermore, the system exhibited sustained stability at different temperatures (300, 305, 310, and 320 K) over the 20 ns simulation, as depicted in Figure 10A. Additionally, conformational principal component analysis (PCA) based on Cα atoms was applied to the molecular dynamics (MD) trajectories of the protein (PDB: 3E0M) and ligand **11** at various temperatures (refer to Figure 11 and Supplementary Figures S45–S48). This study utilized PCA to evaluate the protein’s variance, collective motions, and conformational changes during the MD simulations, as shown in Figure 11 (Table 7 and Supplementary Figure S17). The PCA analysis was performed on the MD trajectories of the target protein (ID: 3E0M) and the ligand **11** complex at 300, 305, 310, and 320 K, using the Bio3D package. The resulting plots illustrated eigenvalues *versus* eigenvectors, highlighting the primary motions extracted from the trajectories, with a focus on the first three eigenvectors (PC1, PC2, and PC3) and color dots representing the variance captured by the eigenvectors.

**TABLE 7:** Variability in principal components revealed via PCA for the target protein (3E0M) and ligand **11** complex at various temperatures.

	Principal components			
Temperature (K)	PC1 (%)	PC2 (%)	PC3 (%)	cosine value
300	69.96	10.56	4.51	0.435
305	28.85	13.62	12.82	0.949
310	24.39	19.19	14.14	0.814
320	33.86	13.82	8.49	0.879

Table 7 provides a concise overview of the primary motions observed in the (ligand **11**-3E0M) complex at various temperatures, specifically focusing on a subset and comparing the first three eigenvectors (PC1, PC2, and PC3). Notably, the complex simulated at 310 K (physiological body temperature) exhibited the most significant variation in PC1 (24.39) and the least in PC2 (19.19) and PC3 (14.14) concerning internal motion within the MD trajectory. This implies a robust interaction between compound **11** and 3E0M, a finding supported by both *in vivo* and *in vitro* investigations. However, further clinical applications are necessary to validate the efficacy and safety of compound **11** as an MSR-3E0M inhibitor.

In previous *in vitro* investigations, ligand **11** demonstrated significant potency and specificity as an inhibitor of biofilm formation and various bacterial diseases. Moreover, this ligand shows promise as a potential cancer treatment. Docking results revealed that ligand **11** exhibited the highest binding affinity (−7.9 kcal mol^−1^) with MSR-3E0M, while its minimum inhibitory concentration (MIC) values against MSSA, MSRA-ATCCBAA1717, and MSRA-ATCCBAA-44 (Table 8) ranged from 1.25 to 2.5 (Song et al., 2020).

**TABLE 8:** Comparative analysis of the selectivity and potency of compounds of naphthoquinone derivatives (**9**, **11**, **13**, and ofloxacin), utilizing docking results and minimum inhibitory values.

Ligand	Docking 3E0M	Docking 4DPT	MSSA (ATCC29213)	MSRA (ATCC BAA1717)	MSRA (ATCC BAA-44)
**9**	−7.6	−6.9	128	>128	>128
**11**	−7.9	−7	1.25–1.9	1.25–2.5	1.25–2.5
**13**	−7.5	−7.4	>128	>128	>128
Ofloxacin	−7.3	−7.1	0.25	0.25	16

In comparison, ofloxacin, with a binding affinity of −7.3 kcal mol^−1^, displayed MIC values ranging from 0.25 to 16. Ofloxacin is a promising candidate for treating cancer and bacterial diseases, given its efficacy and selectivity against methicillin-resistant *staphylococcus aureus* (MRSA). It targets the enzyme responsible for biofilm formation, causes cell membrane damage, chelates intracellular iron ions, and generates intracellular reactive oxygen species. Ligand **11** also demonstrated stable binding to methicillin-resistant *staphylococcus aureus*, with consistent RMSD and RMSF results. Additionally, it maintained a stable conformation at human body temperature. Ligands **9** and **13** also exhibited potential as inhibitors of bacterial diseases, boasting lower Egap values and higher docking affinity than ofloxacin. These ligands formed interactions with amino acid residues that had positive results in previous studies. Particularly, ligand **11** showed the highest binding affinity for the A chain of MSR-3E0M.

**FIGURE 12:**
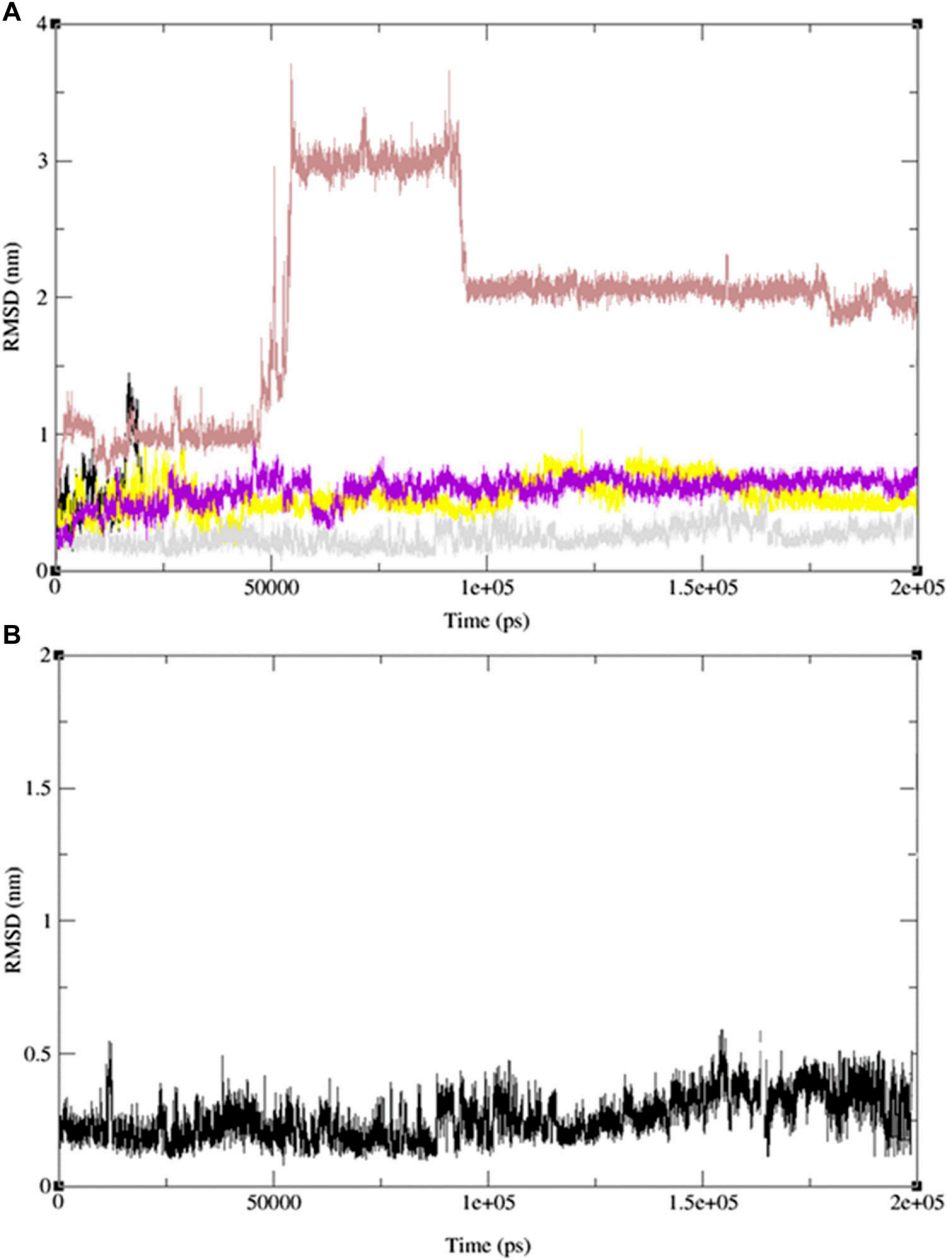
MD simulation evolution of RMSD for graphs **(A)** merged protein-ligand complex of ligands 9,11,13 and ofloxacin between PDB protein: 3E0M and modeled ligands 9 (light red line), 11 (grey line), 13 (yellow line) and reference drug, ofloxacin (purple line), **(B)** Protein-ligand complex of ligand 11 during 200 ns MD simulation.

To validate our findings and determine whether 2-(4-butylbenzyl)-3-hydroxynaphthalene-1,4-dione (**11**) is optimal, we extended our investigation to include two additional naphthoquinone derivatives (**9** and **13**) and the standard drug, ofloxacin, which exhibited the most favorable docking results. Subsequently, we conducted 200 ns MD simulation revealed molecular dynamics simulations, and the outcomes are presented in Figure 12, comprising three RMSD panels: a) Merge protein-ligand complex of ligands **9**, **11**, **13** and ofloxacin, b) protein-ligand complex **11** and c) Protein-ligand complex of ofloxacin. Figure 12 shows the root mean square deviation (RMSD) analysis for the three naphthoquinones and the standard drug. In Figure 12A) the yellow line corresponds to ligand **11**, while the grey, red, and purple lines represent ligands **13**, **9**, and the standard drug, ofloxacin, respectively. Naphthoquinone derivative **11** is the only one exhibiting an RMSD value close to 0.35 nm, whereas the others collectively yield a value over 0.60 nm except the reference drug ofloxacin (0.40 nm). This notably higher combined value indicates that these ligands possess dissimilar shape orientations and exhibit less structural similarity. Notably, the naphthoquinone derivative (**11**) displays a lower RMSD value than the other compounds, as evident in Figures 12A, B. This underscores its unique structural characteristics and suggests its potential as the most promising candidate. For more detailed information, including individual graphs for each naphthoquinone with respect to the reference molecule (PDB: 3E0M), please refer to the supplementary information (Supplementary Figure S45).

**FIGURE 13:**
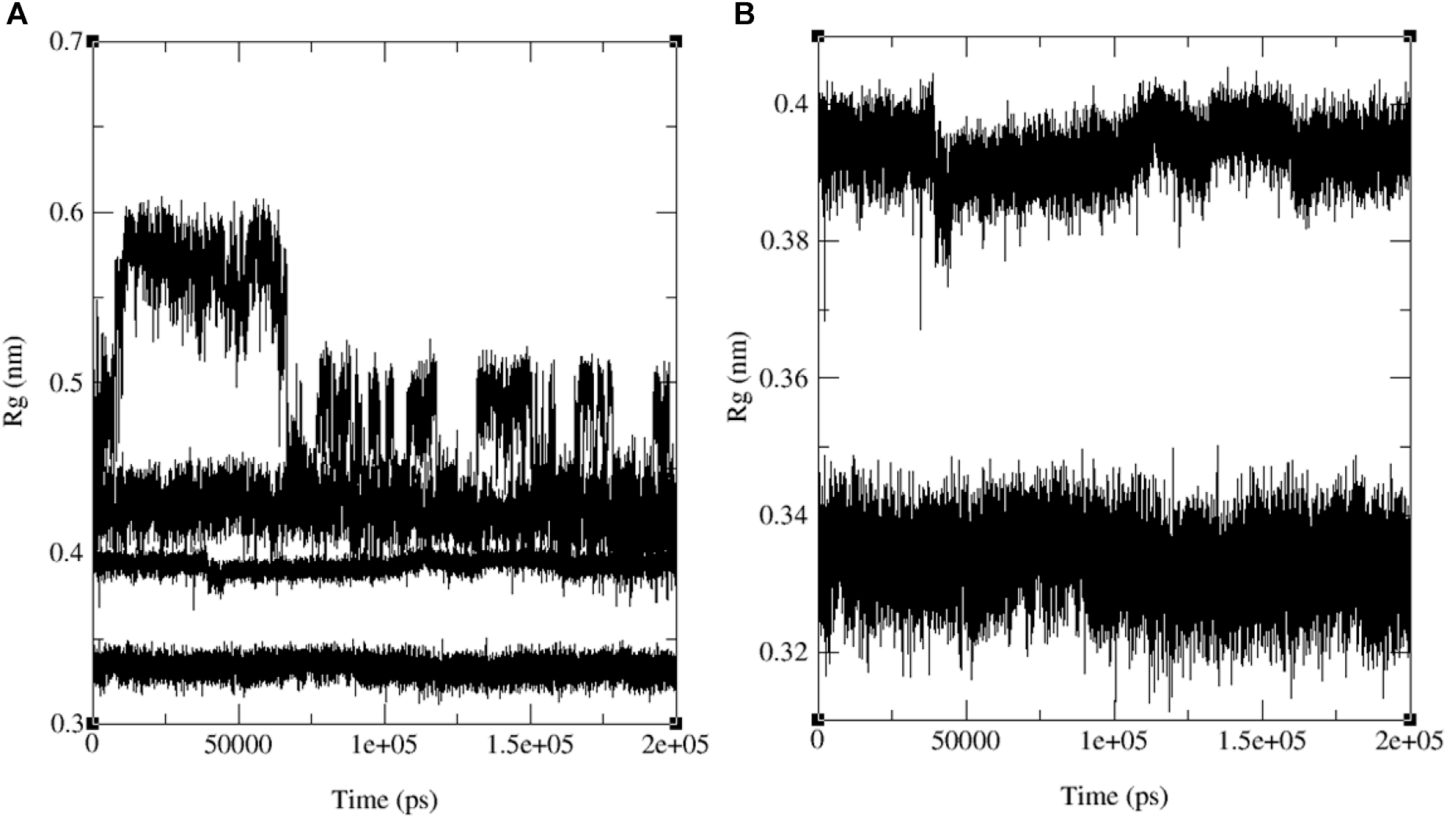
MD simulation evolution of Rg for merged graphs **(A)** ligands **9**, **11**, **13** and ofloxacin and **(B)** merged docked protein‒ligand complex between the PDB protein: 3E0M and modeled ligands **11** and reference drug (ofloxacin) during 200 ns MD simulation.

Figure 13 illustrates the merged radius of gyration for three naphthoquinones and the standard drug. In Figure 13A, we observe a radius of gyration range of 0.32–0.35 nm for naphthoquinone derivative **11**, 0.37–0.41 nm for the standard drug (ofloxacin), and 0.38–0.42 nm for ligand **9** and 0.5–0.6 nm for ligand **13**. It is important to note that higher values in the radius of gyration indicate lower structural similarity and fewer interactions among molecules. Remarkably, the ligand **11** exhibits the highest structural similarity and interactions among its molecules due to its lower radius of gyration. Detailed radius of gyration values and graphs for each naphthoquinone derivative can be found in the supplementary information (see Supplementary Figure S47). In conclusion, simulation results for ligand **11** provided valuable insights, indicating a robust interaction with 3E0M. These findings suggest favorable interactions with the protein, supported by both computational and laboratory experiments. Nevertheless, it is crucial to emphasize that further *in vivo* studies are necessary to validate the efficacy and safety of ligand **11** as an inhibitor of 3E0M. In conclusion, simulation results for ligand **11** provided valuable insights, indicating a robust interaction with 3E0M. These findings suggest favorable interactions with the protein, supported by both computational and laboratory experiments. Nevertheless, it is crucial to emphasize that further *in vivo* studies are necessary to validate the efficacy and safety of ligand **11** as an inhibitor of 3E0M.

## 4 Conclusion

Our in-depth computational analysis explored the medical applications of 13 recently synthesized naphthoquinone derivatives (**1**–**14**), focusing on their interactions with four essential target proteins. Using advanced techniques like FMO calculations, molecular docking, and dynamic MD simulations, our study revealed the exceptional binding affinity of compound **11** with protein 3E0M, surpassing *in vitro* results (Song et al., 2020). This underscores compound **11**’s considerable therapeutic potential, supported by substantial effects on 3E0M cell lines comparable to ofloxacin. All synthesized compounds met strict drug-likeness criteria, showcasing notable antibacterial and anticancer properties in computational assessments. Validating their pharmaceutical potential necessitates essential *in vivo* experiments, marking a significant stride toward innovative therapeutic solutions in the medical field.

## Data Availability

The original contributions presented in the study are included in the article/Supplementary Material, further inquiries can be directed to the corresponding authors.

## References

[B1] Afgan E. Baker D. Batut B. Van Den Beek M. Bouvier D. Čech M. (2018). The Galaxy platform for accessible, reproducible and collaborative biomedical analyses: 2018 update. Nucleic Aacids Res. 46 (W1), W537–W544. 10.1093/nar/gky379 PMC6030816 29790989

[B2] Alberty R. A. (1960). The foundations of chemical kinetics (benson, sidney W.). Chem. Educ. 37, 660. 10.1021/ed037p660.1

[B4] Baroroh U. Biotek M. Muscifa Z. S. Destiarani W. Rohmatullah F. G. Yusuf M. (2023). Molecular interaction analysis and visualization of protein-ligand docking using Biovia Discovery Studio Visualizer. Ind. J. Comput. Bioly (IJCB) 2 (1), 22–30. 10.24198/ijcb.v2i1.46322

[B5] Berman H. M. Westbrook J. Feng Z. Gilliland G. Bhat T. N. Weissig H. (2000). The protein data bank. Nucleic Acids Res. 28 (1), 235–242. 10.1093/nar/28.1.235 10592235 PMC102472

[B6] Biswal S. Gupta P. Panda S. K. Bhat H. R. Rana M. K. (2023). Insights into the binding mechanism of ascorbic acid and violaxanthin with violaxanthin de-epoxidase (VDE) and chlorophycean violaxanthin de-epoxidase (CVDE) enzymes. Photosynth. Res. 156 (3), 337–354. 10.1007/s11120-023-01006-0 36847893

[B7] Bray S. A. Lucas X. Kumar A. Grüning B. A. (2020). The ChemicalToolbox: reproducible, user-friendly cheminformatics analysis on the Galaxy platform. J. Cheminformatics 12 (1), 40–47. 10.1186/s13321-020-00442-7 PMC7268608 33431029

[B8] Brixius-Anderko S. Scott E. E. (2019). Structure of human cortisol-producing cytochrome P450 11B1 bound to the breast cancer drug fadrozole provides insights for drug design. J. Biol. Chem. 294 (2), 453–460. 10.1074/jbc.RA118.006214 30425102 PMC6333875

[B9] Chamizo J. A. Morgado J. Sosa P. (1993). Organometallic aromaticity. Organometallics 12, 125005–125007. 10.1021/om00036a047

[B10] Coenen S. Francis N. Kelly M. Hood K. Nuttall J. Little P. (2013). Are patient views about antibiotics related to clinician perceptions, management and outcome? A multi-country study in outpatients with acute cough. PLOS ONE; Public Libr. Sci. 8, e76691. 10.1371/journal.pone.0076691 PMC3806785 24194845

[B11] Cuendet M. A. van Gunsteren W. F. (2007). On the calculation of velocity-dependent properties in molecular dynamics simulations using the leapfrog integration algorithm. J. Chem. Phys. 127 (18), 184102. 10.1063/1.2779878 18020625

[B12] Daina A. Michielin O. Zoete V. (2017). SwissADME: a free web tool to evaluate pharmacokinetics, drug-likeness and medicinal chemistry friendliness of small molecules. Sci. Rep. 7, 42717. 10.1038/srep42717 28256516 PMC5335600

[B13] Dallakyan S. Olson A. J. (2015). Small-molecule library screening by docking with PyRx. Chem. Biol. Methods Protoc. 1263, 243–250. 10.1007/978-1-4939-2269-7_19 25618350

[B14] Dennington R. Keith T. A. Millam J. M. (2016). GaussView, version 6.0. 16. Semichem Inc Shawnee Mission KS, United states.

[B15] Deurenberg R. H. Stobberingh E. E. (2008). The evolution of staphylococcus aureus. Infect. Genet. Evol. 8 (6), 747–763. 10.1016/j.meegid.2008.07.007 18718557

[B16] Elkaeed E. B. Yousef R. G. Elkady H. Gobaara I. M. Alsfouk B. A. Husein D. Z. (2022). Design, synthesis, docking, DFT, MD simulation studies of a new nicotinamide-based derivative: *in vitro* anticancer and VEGFR-2 inhibitory effects. Molecules 27 (14), 4606. 10.3390/molecules27144606 35889478 PMC9317904

[B17] Esha N. J. I. Quayum S. T. Saif M. Z. Almatarneh M. H. Rahman S. Alodhayb A. (2023). Exploring the potential of fluoro-flavonoid derivatives as anti-lung cancer agents: DFT, molecular docking, and molecular dynamics techniques. Int. J. Quantum Chem. 124, e27274. 10.1002/qua.27274

[B18] Fiorito S. Genovese S. Taddeo V. A. Mathieu V. Kiss R. Epifano F. (2016). Novel juglone and plumbagin 5- O derivatives and their *in vitro* growth inhibitory activity against apoptosis-resistant cancer cells. Bioorg.Med.l Chem. Lett. 26 (2), 334–337. 10.1016/j.bmcl.2015.12.017 26706169

[B19] Frisch M. J. Trucks G. W. Schlegel H. B. Scuseria G. E. Robb M. A. Cheeseman J. R. (2019). Gaussian 16, revision C.01. Wallingford, CT, USA: Gaussian, Inc.

[B20] Goddard T. D. Huang C. C. Ferrin T. E. (2007). Visualizing density maps with UCSF Chimera. J. Struct. Biol. 157 (1), 281–287. 10.1016/j.jsb.2006.06.010 16963278

[B21] Goldberg D. (2015). Plasmepsin V shows its carnivorous side. Nat. Struct. Mol. Biol. 22, 647–648. 10.1038/nsmb.3077 26333709

[B22] Grant B. J. Rodrigues A. P. ElSawy K. M. McCammon J. A. Caves L. S. (2006). Bio3d: an R package for the comparative analysis of protein structures. Bioinformatics 22 (21), 2695–2696. 10.1093/bioinformatics/btl461 16940322

[B23] Hansson T. Oostenbrink C. van Gunsteren W. (2002). Molecular dynamics simulations. Curr. Opin. Struct. Biol. 12 (2), 190–196. 10.1016/s0959-440x(02)00308-1 11959496

[B24] Harada D. Takigawa N. Kiura K. (2014). The role of STAT3 in non-small cell lung cancer. Cancers 6 (2), 708–722. 10.3390/cancers6020708 24675568 PMC4074799

[B25] Jahanban-Esfahlan A. Davaran S. Moosavi-Movahedi A. A. Dastmalchi S. (2017). Investigating the interaction of juglone (5-hydroxy-1, 4-naphthoquinone) with serum albumins using spectroscopic and *in silico* methods. J. Iran. Chem. Soc. 14 (7), 1527–1540. 10.1007/s13738-017-1094-0

[B26] Khosla G. Shukla V. K. Sharma V. (2022). Characterization of Plumbagin by implying various *in silico* studies. Int. J. Health Sci. 6 (S1). 10.53730/ijhs.v6nS1.6558

[B27] Kim S. Chen J. Cheng T. Gindulyte A. He J. He S. (2021). PubChem in 2021: new data content and improved web interfaces. Nucleic Acids Res. 49 (D1), D1388–D1395. 10.1093/nar/gkaa971 33151290 PMC7778930

[B28] Kim S. Thiessen P. A. Bolton E. E. Chen J. Fu G. Gindulyte A. (2016). PubChem substance and compound databases. Nucleic Acids Res. 44, D1202–D1213. 10.1093/nar/gkv951 26400175 PMC4702940

[B29] Kumar N. Awasthi A. Kumari A. Sood D. Jain P. Singh T. (2022). Antitussive noscapine and antiviral drug conjugates as arsenal against COVID-19: a comprehensive chemoinformatics analysis. J. Biomol. Struc. Dyn. 40 (1), 101–116. 10.1080/07391102.2020.1808072 PMC7484584 32815796

[B30] Laskowski R. A. Furnham N. Thornton J. M. (2013). “The Ramachandran plot and protein structure validation,” in Biomolecular forms and functions: a celebration of 50 Years of the ramachandran map (Singapore: World Scientific), 62–75. 10.1142/9789814449144_0005

[B31] Lipinski C. A. (2004). Lead- and drug-like compounds: the rule-of-five revolution. Technologies 1 (4), 337–341. 10.1016/j.ddtec.2004.11.007 24981612

[B32] Loomba P. S. Taneja J. Mishra B. (2010). Methicillin and vancomycin resistant *S. aureus* in hospitalized patients. J. Glob. Infect. Dis. 2 (3), 275. 10.4103/0974-777x.68535 20927290 PMC2946685

[B33] Malik M. S. Alsantali R. I. Jassas R. S. Alsimaree A. A. Syed R. Alsharif M. A. (2021). Journey of anthraquinones as anticancer agents – a systematic review of recent literature. RSC Adv. 11 (57), 35806–35827. 10.1039/d1ra05686g 35492773 PMC9043427

[B34] Panda S. K. Gupta P. S. Rana M. K. (2023). Potential targets of severe acute respiratory syndrome coronavirus 2 of clinical drug fluvoxamine: docking and molecular dynamics studies to elucidate viral action. Cell biochem. Funct. 41 (1), 98–111. 10.1002/cbf.3766 36478589

[B35] Parr R. G. Szentpály L. V. Liu S. (1999). Electrophilicity index. J. Am. Chem. Soc. 121 (9), 1922–1924. 10.1021/ja983494x

[B36] Presti D. Pedone A. Mancini G. Duce C. Tiné M. R. Barone V. (2016). Insights into structural and dynamical features of water at halloysite interfaces probed by DFT and classical molecular dynamics simulations. Phys. Chem. Chem. Phys. 18 (3), 2164–2174. 10.1039/C5CP05920H 26690815

[B37] Rathod S. Shinde K. Porlekar J. Choudhari P. Dhavale R. Mahuli D. (2022). Computational exploration of anti-cancer potential of flavonoids against cyclin-dependent kinase 8: an *in silico* molecular docking and dynamic approach. ACS omega 8 (1), 391–409. 10.1021/acsomega.2c04837 36643495 PMC9835631

[B38] Rossi F. Diaz L. Wollam A. Panesso D. Zhou Y. Rincon S. (2014). Transferable vancomycin resistance in a community-associated MRSA lineage. N. Eng. J. Med. 370 (16), 1524–1531. 10.1056/nejmoa1303359 PMC4112484 24738669

[B39] Sahoo P. M. S. Behera S. Behura R. Acharya A. Biswal D. Suna S. K. (2022). A brief review: antibacterial activity of Quinone derivatives. Biointerface Res. Appl. Chem. 12, 3247–3258. 10.33263/BRIAC123.32473258

[B40] Shadrack D. M. Ndesendo V. M. (2017). Molecular docking and ADMET study of emodin derivatives as anticancer inhibitors of NAT2, COX2 and TOP1 enzymes. Comput. Mol. Biosci. 7 (1), 1–18. 10.4236/cmb.2017.71001

[B41] Showalter S. A. Brüschweiler R. (2007). Validation of molecular dynamics simulations of biomolecules using NMR spin relaxation as benchmarks: application to the AMBER99SB force field. J. Chem. Theor. Comput. 3 (3), 961–975. 10.1021/ct7000045 26627416

[B42] Song R. Yu B. Friedrich D. Li J. Shen H. Krautscheid H. (2020). Naphthoquinone-derivative as a synthetic compound to overcome the antibiotic resistance of methicillin-resistant *S. aureus* . Commun. Biol. 3 (1), 529. 10.1038/s42003-020-01261-0 32973345 PMC7518446

[B43] Stefani S. Chung D. R. Lindsay J. A. Friedrich A. Kearns A. Westh H. (2012). Meticillin-resistant staphylococcus aureus (MRSA): global epidemiology and harmonisation of typing methods. Int. J. Antimicrob. Agents 39, 273–282. 10.1016/j.ijantimicag.2011.09.030 22230333

[B44] Stefani S. Goglio A. (2010). Methicillin-resistant staphylococcus aureus: related infections and antibiotic resistance. Int. J. Infect. Dis. 14, S19–S22. 10.1016/j.ijid.2010.05.009 20843722

[B45] Uddin K. M. Sakib M. Siraji S. Rahman S. Alodhayb A. Alibrahim K. A. (2023). Synthesis of new derivatives of benzylidinemalononitrile and ethyl 2-Cyano-3-phenylacrylate: *in silico* anticancer evaluation. ACS omega 8 (29), 25817–25831. 10.1021/acsomega.3c01123 37521603 PMC10373203

[B46] Van Der Spoel D. Lindahl E. Hess B. Groenhof G. Mark A. E. Berendsen H. J. (2005). GROMACS: fast, flexible, and free. J. Comput. Chem. 26 (16), 1701–1718. 10.1002/jcc.20291 16211538

[B47] Veber D. F. Johnson S. R. Cheng H. Y. Smith B. R. Ward K. W. Kopple K. D. (2002). Molecular properties that influence the oral bioavailability of drug candidates. J. Med. Chem. 45 (12), 2615–2623. 10.1021/jm020017n 12036371

[B48] Wang W. Chang C. T. Zhang Q. (2022). 1,4-Naphthoquinone analogs and their application as antibacterial agents. ChemistrySelect, 7(43). 10.1002/slct.202203330

[B49] Yang H. Lou C. Sun L. Li J. Cai Y. Wang Z. (2019). AdmetSAR 2.0: web-service for prediction and optimization of chemical ADMET properties. Bioinformatics 35 (6), 1067–1069. 10.1093/bioinformatics/bty707 30165565

[B50] Zardecki C. Dutta S. Goodsell D. S. Voigt M. Burley S. K. (2016). RCSB protein Data Bank: a resource for chemical, biochemical, and structural explorations of large and small biomolecules. J. Chem. Educ. 93 (3), 569–575. 10.1021/acs.jchemed.5b00404

