# Supplementary material for: Exploring the anticancer and antibacterial potential of naphthoquinone derivatives: a comprehensive computational investigation
Source: Front Chem. 2024 Feb 21;12:1351669. doi: 10.3389/fchem.2024.1351669 (PMC10914998; doi:10.3389/fchem.2024.1351669)
Supplement: Supplementary file 1 [file Table1.docx]

**Exploring the Anticancer and Antibacterial Potential of Naphthoquinone Derivatives: A Comprehensive**

**Computational Investigation**

Mehnaz Hossain Meem^1^, Sumaiya Binte Yusuf^1^, Sanaa S. Al Abbad^2^, Shofiur

Rahman^3*^, Mahmoud Al-Gawati^3,4^, Hamad Albrithen^3,4^,

Abdullah N. Alodhayb^3,4*^ and Kabir M. Uddin^1*^

^1^ Department of Biochemistry and Microbiology, North South University, Bashundhara, Dhaka-1217, Bangladesh

^2^ Department of Chemistry, Imam Abdulrahman Bin Faisal University, Dammam 31441, Saudi Arabia.

^3^ Biological and Environmental Sensing Research Unit, King Abdullah Institute for Nanotechnology, King Saud University, P.O. Box 2455, Riyadh 11451, Saudi Arabia.

^4^ Research Chair for Tribology, Surface, and Interface Sciences, Department of Physics and Astronomy, College of Science, King Saud University, Riyadh 11451, Saudi Arabia.

***** Correspondence: [kabirmuddin@gmail.com](mailto:kabirmuddin@gmail.com) (K.M.U.); [mohammed.uddin11@northsouth.edu](mailto:mohammed.uddin11@northsouth.edu) (K.M.U.); [mrahman1@ksu.edu.sa](mailto:mrahman1@ksu.edu.sa) (S.R.); [aalodhayb@ksu.edu.sa](mailto:aalodhayb@ksu.edu.sa) (A.N.A.)



**Table S1.** Optimized structure for compound **1** and cartesian Z-matrix.

| **(1)** | | | 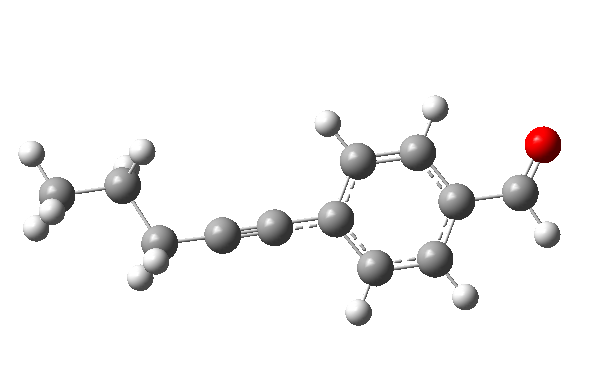 | | |
| --- | --- | --- | --- | --- | --- |
| Center  Number | Atom | Standard orientation: Coordinates (Angstroms) | | | |
|  |  | X | | Y | Z |
| 1 | C | 2.29743686 | | -1.06937567 | -0.02172942 |
| 2 | C | 0.91641504 | | -0.95530234 | -0.03354676 |
| 3 | C | 0.30249626 | | 0.31736681 | -0.0151005 |
| 4 | C | 1.1157523 | | 1.46824144 | 0.01539189 |
| 5 | C | 2.49961437 | | 1.34566424 | 0.0269291 |
| 6 | C | 3.10255471 | | 0.08069762 | 0.00861755 |
| 7 | H | 2.78292167 | | -2.03990743 | -0.03558347 |
| 8 | H | 0.28975862 | | -1.8406149 | -0.05706359 |
| 9 | H | 0.64709639 | | 2.44638519 | 0.02959747 |
| 10 | H | 3.12354109 | | 2.2361264 | 0.05042366 |
| 11 | C | 4.57550543 | | -0.03741017 | 0.0212618 |
| 12 | H | 5.11647257 | | 0.93509373 | 0.04563248 |
| 13 | O | 5.18665589 | | -1.08989576 | 0.00696058 |
| 14 | C | -1.11913146 | | 0.4349975 | -0.02711189 |
| 15 | C | -2.32781718 | | 0.54258589 | -0.03778993 |
| 16 | C | -3.78570232 | | 0.64312066 | -0.04912502 |
| 17 | H | -4.1059069 | | 1.15629711 | -0.96691309 |
| 18 | H | -4.11107592 | | 1.28501027 | 0.7815671 |
| 19 | C | -4.4972433 | | -0.72305572 | 0.04884722 |
| 20 | H | -4.17258775 | | -1.22603594 | 0.96683024 |
| 21 | H | -4.16704482 | | -1.35434279 | -0.78394837 |
| 22 | C | -6.02113855 | | -0.58553617 | 0.03393372 |
| 23 | H | -6.50525953 | | -1.56427022 | 0.10400142 |
| 24 | H | -6.37463086 | | 0.02123631 | 0.87519083 |
| 25 | H | -6.36898475 | | -0.10777622 | -0.88888595 |

**Table S2.** Optimized structure for compound **2** and cartesian Z-matrix.

| **(2)** | | | 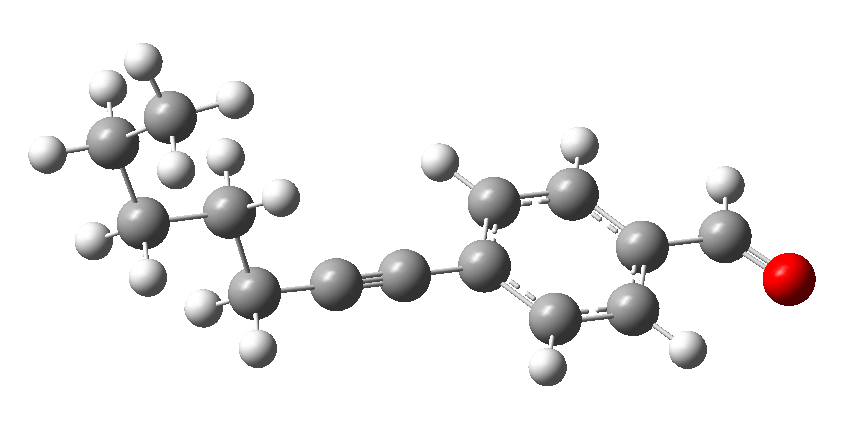 | | |
| --- | --- | --- | --- | --- | --- |
| Center  Number | Atom | Standard orientation: Coordinates (Angstroms) | | | |
|  |  | X | | Y | Z |
| 1 | C | 3.53685306 | | -0.53271746 | 0.89096274 |
| 2 | C | 2.19385307 | | -0.86864977 | 0.82854515 |
| 3 | C | 1.3721523 | | -0.34880782 | -0.19695154 |
| 4 | C | 1.93920964 | | 0.51626981 | -1.15448661 |
| 5 | C | 3.28638464 | | 0.84876842 | -1.08511208 |
| 6 | C | 4.09603269 | | 0.32975575 | -0.06575549 |
| 7 | H | 4.18004992 | | -0.92424906 | 1.67250966 |
| 8 | H | 1.75666688 | | -1.53434866 | 1.56523732 |
| 9 | H | 1.31071715 | | 0.91656125 | -1.94276278 |
| 10 | H | 3.72000583 | | 1.51658252 | -1.82589645 |
| 11 | C | 5.52766838 | | 0.69037053 | -0.00196316 |
| 12 | H | 5.86662567 | | 1.37918603 | -0.80799462 |
| 13 | O | 6.30617739 | | 0.29047784 | 0.84385387 |
| 14 | C | -0.01045694 | | -0.6944414 | -0.26126184 |
| 15 | C | -1.18415284 | | -0.9981757 | -0.31433888 |
| 16 | C | -2.60560658 | | -1.33227196 | -0.37543416 |
| 17 | H | -2.83650546 | | -2.0794631 | 0.39584492 |
| 18 | H | -2.8230389 | | -1.81405989 | -1.338859 |
| 19 | C | -3.52965356 | | -0.10733022 | -0.19649646 |
| 20 | H | -3.29690268 | | 0.63015299 | -0.97469716 |
| 21 | H | -3.29720186 | | 0.36870516 | 0.76216988 |
| 22 | C | -5.01411741 | | -0.48628554 | -0.26583827 |
| 23 | H | -5.20196928 | | -1.00773423 | -1.21436244 |
| 24 | H | -5.24182869 | | -1.21050445 | 0.52961781 |
| 25 | C | -5.97944393 | | 0.70521576 | -0.15611145 |
| 26 | H | -5.75416059 | | 1.42586619 | -0.95399724 |
| 27 | H | -6.99746946 | | 0.34607842 | -0.35229511 |
| 28 | C | -5.95679414 | | 1.41956744 | 1.20005058 |
| 29 | H | -6.18065411 | | 0.72306825 | 2.0164243 |
| 30 | H | -4.98255423 | | 1.87270655 | 1.40938546 |
| 31 | H | -6.70276938 | | 2.22002236 | 1.2339934 |

**Table S3.** Optimized structure for compound **3** and cartesian Z-matrix.

| **(3)** | | 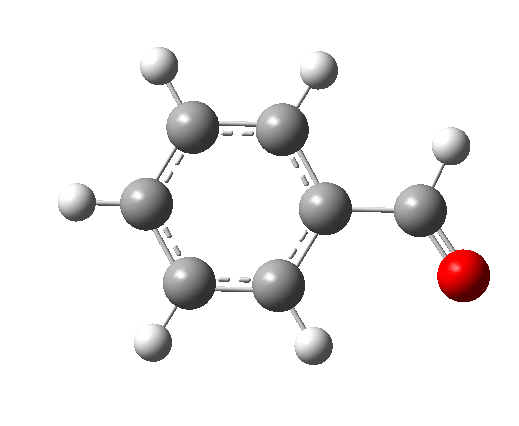 | | |
| --- | --- | --- | --- | --- |
| Center  Number | Atom | Standard orientation: Coordinates (Angstroms) | | |
|  |  | X | Y | Z |
| 1 | C | 0.0458938 | -1.10077762 | 0.00023974 |
| 2 | C | -1.32523837 | -1.33130599 | -0.00007648 |
| 3 | C | -2.21608701 | -0.25138937 | -0.00013388 |
| 4 | C | -1.73602875 | 1.05998024 | 0.00006793 |
| 5 | C | -0.3615473 | 1.29222917 | 0.0000706 |
| 6 | C | 0.53370865 | 0.21507103 | -0.00013414 |
| 7 | H | 0.76086911 | -1.91727008 | 0.00045609 |
| 8 | H | -1.70652047 | -2.34807456 | -0.00022348 |
| 9 | H | -3.28673228 | -0.43454626 | -0.00000794 |
| 10 | H | -2.43053534 | 1.89453047 | 0.00018814 |
| 11 | H | 0.02412752 | 2.30916148 | 0.00008746 |
| 12 | C | 1.99200196 | 0.46957168 | -0.00010624 |
| 13 | H | 2.2727088 | 1.54682599 | 0.00001313 |
| 14 | O | 2.84623309 | -0.39636273 | -0.00000981 |

**Table S4.** Optimized structure for compound **4** and cartesian Z-matrix.

| **(4)** | | | 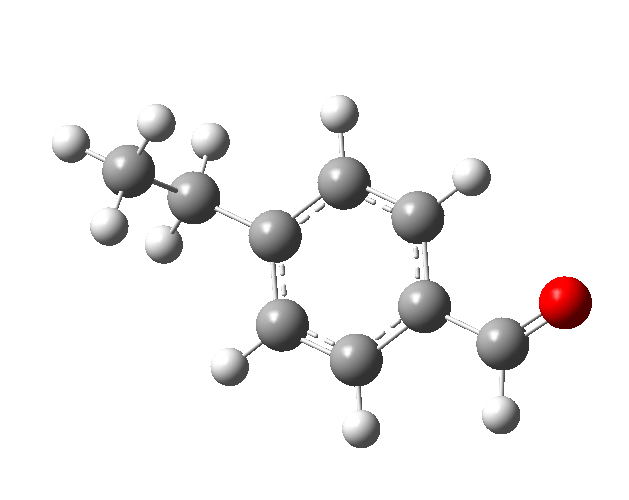 | | |
| --- | --- | --- | --- | --- | --- |
| Center  Number | C  C | -0.8817568 | | | |
|  |  | 0.49297416 | | -1.12760113 | -0.31676504 |
| 1 | C | 1.29101196 | | 0.02955707 | -0.34834265 |
| 2 | C | 0.66770602 | | 1.27676868 | -0.20073044 |
| 3 | C | -0.71041977 | | 1.36741233 | -0.02418763 |
| 4 | C | -1.49602882 | | 0.20865583 | 0.00756634 |
| 5 | H | -1.50547545 | | -1.93241058 | -0.11921899 |
| 6 | H | 0.96382045 | | -2.10020107 | -0.43681965 |
| 7 | H | 1.27023497 | | 2.18104542 | -0.22897442 |
| 8 | H | -1.18492 | | 2.3400334 | 0.08569728 |
| 9 | C | -2.95875422 | | 0.31023258 | 0.19136481 |
| 10 | H | -3.33893718 | | 1.35134192 | 0.29763537 |
| 11 | O | -3.72151954 | | -0.63731182 | 0.23023793 |
| 12 | C | 2.79273505 | | -0.07332949 | -0.49844619 |
| 13 | H | 3.03682722 | | -0.922312 | -1.14767371 |
| 14 | H | 3.17223799 | | 0.8243392 | -1.00000744 |
| 15 | C | 3.51399512 | | -0.24414816 | 0.85123677 |
| 16 | H | 3.17711302 | | -1.14998077 | 1.3650093 |
| 17 | H | 4.5964993 | | -0.31799622 | 0.70667208 |
| 18 | H | 3.31597983 | | 0.60549291 | 1.51223901 |
| 19 | C | -0.8817568 | | -1.04435734 | -0.14110634 |
| 20 | C | 0.49297416 | | -1.12760113 | -0.31676504 |

**Table S5.** Optimized structure for (**5**) and cartesian Z-matrix.

| **(5)** | | 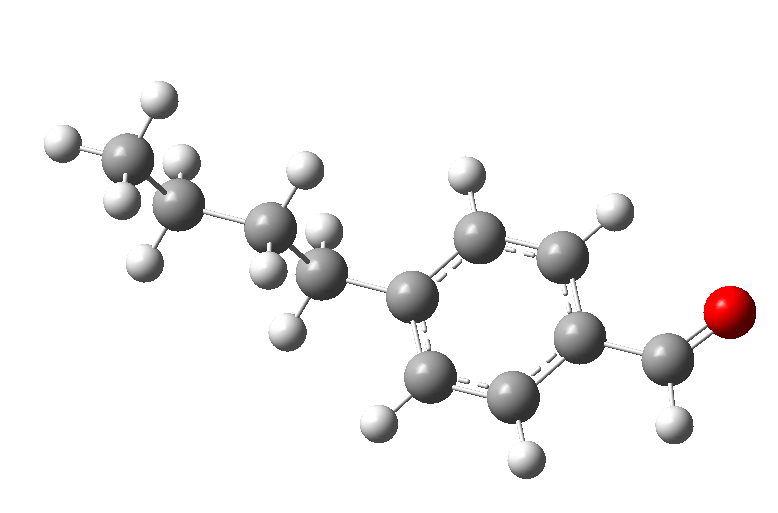 | | |
| --- | --- | --- | --- | --- |
| Center  Number | Atom | Standard orientation: Coordinates (Angstroms) | | |
|  |  | X | Y | Z |
| 1 | C | -1.88471222 | -1.01501118 | -0.29668627 |
| 2 | C | -0.54616532 | -0.98216159 | -0.66395056 |
| 3 | C | 0.18851377 | 0.21598241 | -0.62031178 |
| 4 | C | -0.4617801 | 1.38444037 | -0.19821178 |
| 5 | C | -1.80380761 | 1.35852722 | 0.17149133 |
| 6 | C | -2.52595132 | 0.15956447 | 0.12590075 |
| 7 | H | -2.45965985 | -1.93499563 | -0.33122744 |
| 8 | H | -0.05497062 | -1.89353016 | -0.99580804 |
| 9 | H | 0.0907316 | 2.31992316 | -0.16489479 |
| 10 | H | -2.29990322 | 2.27130146 | 0.49389777 |
| 11 | C | -3.95114632 | 0.13802374 | 0.51551793 |
| 12 | H | -4.35747643 | 1.12507707 | 0.83236494 |
| 13 | O | -4.65906446 | -0.85195136 | 0.50684503 |
| 14 | C | 1.65637209 | 0.23382247 | -0.98124005 |
| 15 | H | 1.84763458 | -0.49763153 | -1.77627075 |
| 16 | H | 1.9229249 | 1.21667904 | -1.38900218 |
| 17 | C | 2.57404166 | -0.07834736 | 0.21893086 |
| 18 | H | 2.3037574 | -1.05910939 | 0.63239342 |
| 19 | H | 2.3813463 | 0.6507743 | 1.01719156 |
| 20 | C | 4.06362967 | -0.06465218 | -0.14370208 |
| 21 | H | 4.24766215 | -0.79301941 | -0.94508831 |
| 22 | H | 4.32569366 | 0.91721849 | -0.56073779 |
| 23 | C | 4.97406786 | -0.37409015 | 1.04832816 |
| 24 | H | 4.7589981 | -1.36450658 | 1.46487046 |
| 25 | H | 6.02968953 | -0.35733828 | 0.75913021 |
| 26 | H | 4.83771448 | 0.35817907 | 1.8520217 |

**Table S6.** Optimized structure for compound **6** and cartesian Z-matrix.

| **(6)** | | 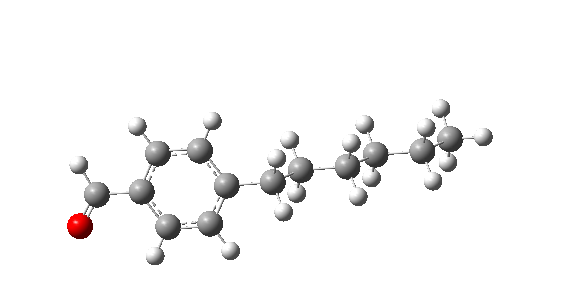 | | |
| --- | --- | --- | --- | --- |
| Center  Number | Atom | Standard orientation: Coordinates (Angstroms) | | |
|  |  | X | Y | Z |
| 1 | C | -2.93575145 | -1.14525975 | 0.72146507 |
| 2 | C | -3.61428556 | -0.00492765 | 0.27385263 |
| 3 | C | -2.97126988 | 0.8773346 | -0.60791717 |
| 4 | C | -1.67389473 | 0.61677962 | -1.02818763 |
| 5 | C | -0.98304434 | -0.52441075 | -0.58364246 |
| 6 | C | -1.6348753 | -1.40052205 | 0.29608925 |
| 7 | H | -3.43378878 | -1.83326943 | 1.40103883 |
| 8 | H | -3.51256877 | 1.75394081 | -0.94951542 |
| 9 | H | -1.18166647 | 1.30030204 | -1.71557234 |
| 10 | H | -1.11652281 | -2.29033552 | 0.64401903 |
| 11 | C | 0.44407879 | -0.77755489 | -1.0130796 |
| 12 | H | 0.63609941 | -1.85736958 | -1.03322651 |
| 13 | H | 0.58864188 | -0.41435313 | -2.03817241 |
| 14 | C | 1.47812318 | -0.09982549 | -0.09004977 |
| 15 | H | 1.33134676 | -0.45974263 | 0.93689477 |
| 16 | H | 1.28166395 | 0.98015108 | -0.0639028 |
| 17 | C | 2.92675945 | -0.35125447 | -0.52418161 |
| 18 | H | 3.11460642 | -1.43406389 | -0.55357209 |
| 19 | H | 3.06478954 | 0.0075704 | -1.55411574 |
| 20 | C | 3.96091389 | 0.31824979 | 0.38904755 |
| 21 | H | 3.82277291 | -0.04059575 | 1.41898432 |
| 22 | H | 3.77266195 | 1.40094436 | 0.4188715 |
| 23 | C | 5.41157213 | 0.06947181 | -0.04221058 |
| 24 | H | 5.59924834 | -1.0124851 | -0.07196995 |
| 25 | H | 5.54928654 | 0.42861148 | -1.07116901 |
| 26 | C | 6.43824119 | 0.74084851 | 0.87471319 |
| 27 | H | 7.46221208 | 0.54493926 | 0.54063936 |
| 28 | H | 6.29839134 | 1.82755605 | 0.89773515 |
| 29 | H | 6.34898042 | 0.3757395 | 1.90417094 |
| 30 | C | -4.99625422 | 0.259498 | 0.72510642 |
| 31 | H | -5.40822186 | -0.50548093 | 1.42143601 |
| 32 | O | -5.66497644 | 1.21967216 | 0.39017432 |

**Table S7.** Optimized structure for compound **7** and cartesian Z-matrix.

| **(7)** | | | 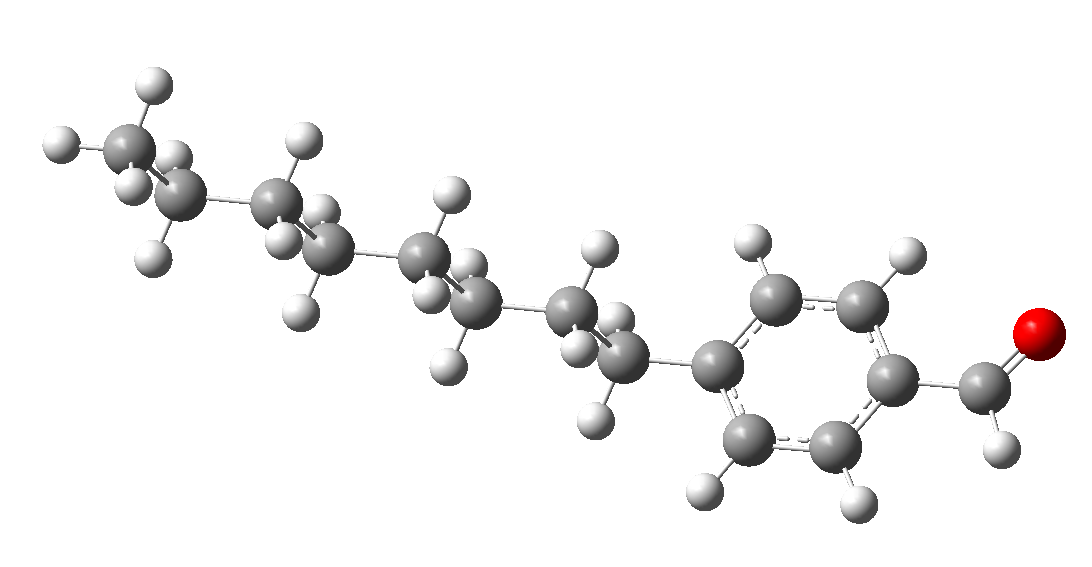 | |
| --- | --- | --- | --- | --- |
| Center  Number | Atom | Standard orientation: Coordinates (Angstroms) | | |
|  |  | X | Y | Z |
| 1 | C | -4.10619742 | -0.63151998 | -0.88062035 |
| 2 | C | -2.84177472 | -0.1460774 | -1.18617946 |
| 3 | C | -2.1812553 | 0.74889415 | -0.32602098 |
| 4 | C | -2.82941023 | 1.14548652 | 0.85255305 |
| 5 | C | -4.09725401 | 0.66252088 | 1.16505282 |
| 6 | C | -4.74572931 | -0.22962362 | 0.30207175 |
| 7 | H | -4.62412447 | -1.3195626 | -1.54128641 |
| 8 | H | -2.35239584 | -0.45605601 | -2.10618514 |
| 9 | H | -2.33456504 | 1.84155918 | 1.52494871 |
| 10 | H | -4.59292904 | 0.97938181 | 2.07999583 |
| 11 | C | -6.0929733 | -0.73616515 | 0.63640118 |
| 12 | H | -6.50506744 | -0.35122249 | 1.59650837 |
| 13 | O | -6.73452526 | -1.51010959 | -0.04968453 |
| 14 | C | -0.78752783 | 1.23912361 | -0.64604113 |
| 15 | H | -0.67523013 | 1.34185032 | -1.73254255 |
| 16 | H | -0.64276989 | 2.23956289 | -0.22010656 |
| 17 | C | 0.3159822 | 0.30122562 | -0.11394595 |
| 18 | H | 0.16731835 | -0.70202511 | -0.53471538 |
| 19 | H | 0.20153525 | 0.19430553 | 0.97287957 |
| 20 | C | 1.73098303 | 0.79351954 | -0.43909501 |
| 21 | H | 1.83638649 | 0.90259336 | -1.52790697 |
| 22 | H | 1.87098869 | 1.79995234 | -0.01949627 |
| 23 | C | 2.83370343 | -0.13345475 | 0.08713466 |
| 24 | H | 2.6923596 | -1.13957567 | -0.33241006 |
| 25 | H | 2.72689287 | -0.24244931 | 1.17573021 |
| 26 | C | 4.25075718 | 0.35585323 | -0.23638288 |
| 27 | H | 4.35696273 | 0.46495952 | -1.32518366 |
| 28 | H | 4.39147918 | 1.36237521 | 0.18293403 |
| 29 | C | 5.3542271 | -0.57008372 | 0.28935203 |
| 30 | H | 5.21423958 | -1.57687853 | -0.12975976 |
| 31 | H | 5.24875777 | -0.67956706 | 1.37830065 |
| 32 | C | 6.77167054 | -0.08168986 | -0.03368573 |
| 33 | H | 6.87709878 | 0.02714375 | -1.12172452 |
| 34 | H | 6.91159199 | 0.92406112 | 0.38548342 |
| 35 | C | 7.86750354 | -1.01198839 | 0.49485001 |
| 36 | H | 7.77546681 | -2.01644797 | 0.06622631 |
| 37 | H | 8.86587294 | -0.63620973 | 0.24847861 |
| 38 | H | 7.8101031 | -1.11299796 | 1.58464367 |

**Table S8.** Optimized structure for compound **8** and cartesian Z-matrix.

| (**8**) | | | 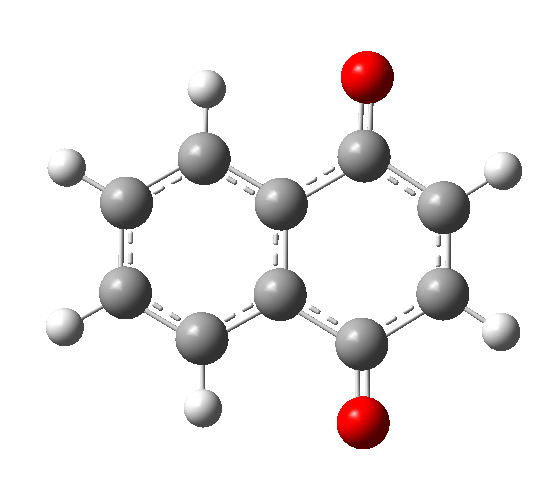 | | |
| --- | --- | --- | --- | --- | --- |
| Center  Number | Atom | Standard orientation: Coordinates (Angstroms) | | | |
|  |  | X | | Y | Z |
| 1 | C | 2.67724785 | | -0.69954019 | 0.0000671 |
| 2 | C | 1.47275873 | | -1.40022803 | 0.0002305 |
| 3 | C | 0.26022587 | | -0.70427462 | 0.00011352 |
| 4 | C | 0.26022073 | | 0.70427022 | -0.00014594 |
| 5 | C | 1.47276121 | | 1.4002192 | -0.00032087 |
| 6 | C | 2.67724671 | | 0.69953181 | -0.00020923 |
| 7 | H | 3.61837256 | | -1.2413066 | 0.00015129 |
| 8 | H | 1.44553273 | | -2.48477736 | 0.00043327 |
| 9 | C | -1.02494608 | | -1.4628228 | 0.00025133 |
| 10 | C | -1.02494069 | | 1.46282688 | -0.00023911 |
| 11 | H | 1.44553887 | | 2.48476848 | -0.0005326 |
| 12 | H | 3.61837334 | | 1.24129477 | -0.00033444 |
| 13 | C | -2.28139122 | | 0.67161317 | -0.00001447 |
| 14 | C | -2.2813924 | | -0.67159954 | 0.0002525 |
| 15 | H | -3.19906849 | | 1.25284508 | -0.00008019 |
| 16 | H | -3.19907007 | | -1.25282983 | 0.00045459 |
| 17 | O | -1.06101482 | | 2.68782742 | -0.0005202 |
| 18 | O | -1.06103808 | | -2.68782382 | 0.00051971 |

**Table S9.** Optimized structure for compound **9** and cartesian Z-matrix.

| **(9)** | | 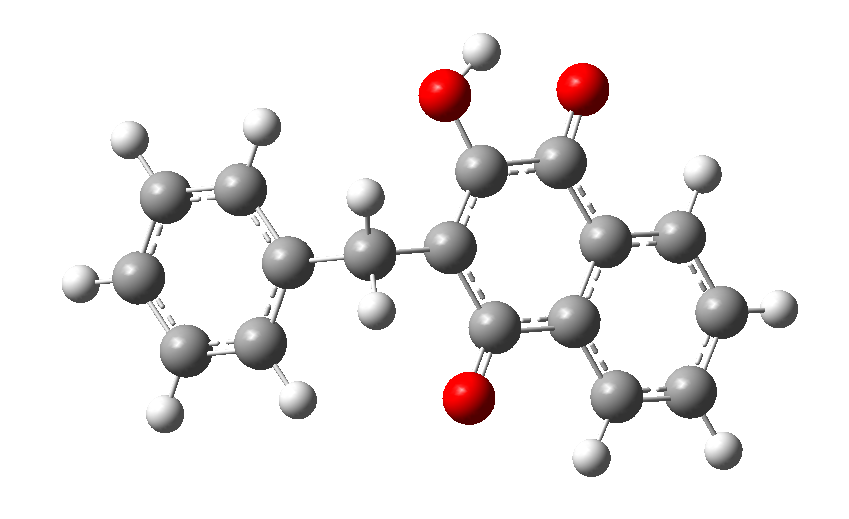 | | |
| --- | --- | --- | --- | --- |
| Center  Number | Atom | Standard orientation: Coordinates (Angstroms) | | |
|  |  | X | Y | Z |
| 1 | C | -4.54525544 | 0.62282648 | -0.95635559 |
| 2 | C | -3.76296414 | -0.52356402 | -0.83085813 |
| 3 | C | -2.49057849 | -0.43373067 | -0.25764378 |
| 4 | C | -1.9963111 | 0.80428016 | 0.19336315 |
| 5 | C | -2.78747614 | 1.94591003 | 0.06373585 |
| 6 | C | -4.05689876 | 1.85442731 | -0.50952797 |
| 7 | H | -5.53346425 | 0.55859285 | -1.40112318 |
| 8 | H | -4.11688693 | -1.49186056 | -1.16836066 |
| 9 | C | -1.64927734 | -1.63380852 | -0.11640704 |
| 10 | C | -0.63197434 | 0.91585984 | 0.81284062 |
| 11 | H | -2.39025907 | 2.89115136 | 0.41696405 |
| 12 | H | -4.66818681 | 2.74641766 | -0.60872743 |
| 13 | C | 0.1969641 | -0.2985729 | 0.94196997 |
| 14 | C | -0.29559938 | -1.48225472 | 0.49389025 |
| 15 | O | -0.22724609 | 2.00721721 | 1.20987905 |
| 16 | O | -1.98863284 | -2.76453826 | -0.46947938 |
| 17 | C | 1.57569312 | -0.17240287 | 1.548452 |
| 18 | H | 1.54792149 | 0.63604504 | 2.28343259 |
| 19 | H | 1.81770146 | -1.10277386 | 2.06965602 |
| 20 | O | 0.38896095 | -2.63419864 | 0.56999726 |
| 21 | H | -0.21150647 | -3.31039412 | 0.1896698 |
| 22 | C | 2.65093912 | 0.12254707 | 0.50920413 |
| 23 | C | 2.91729988 | 1.43990236 | 0.11317231 |
| 24 | C | 3.37906641 | -0.92002708 | -0.07799418 |
| 25 | C | 3.89551393 | 1.70797726 | -0.84486482 |
| 26 | H | 2.34824231 | 2.25105602 | 0.55754721 |
| 27 | C | 4.35730191 | -0.65212778 | -1.03649552 |
| 28 | H | 3.17399194 | -1.94514954 | 0.21755577 |
| 29 | C | 4.61921381 | 0.66323137 | -1.42253519 |
| 30 | H | 4.09412795 | 2.7350624 | -1.13828496 |
| 31 | H | 4.91698846 | -1.47154615 | -1.47895991 |
| 32 | H | 5.38273081 | 0.87271664 | -2.16622108 |

**Table S10.** Optimized structure for compound **10** and cartesian Z-matrix.

| **(10)** | | | 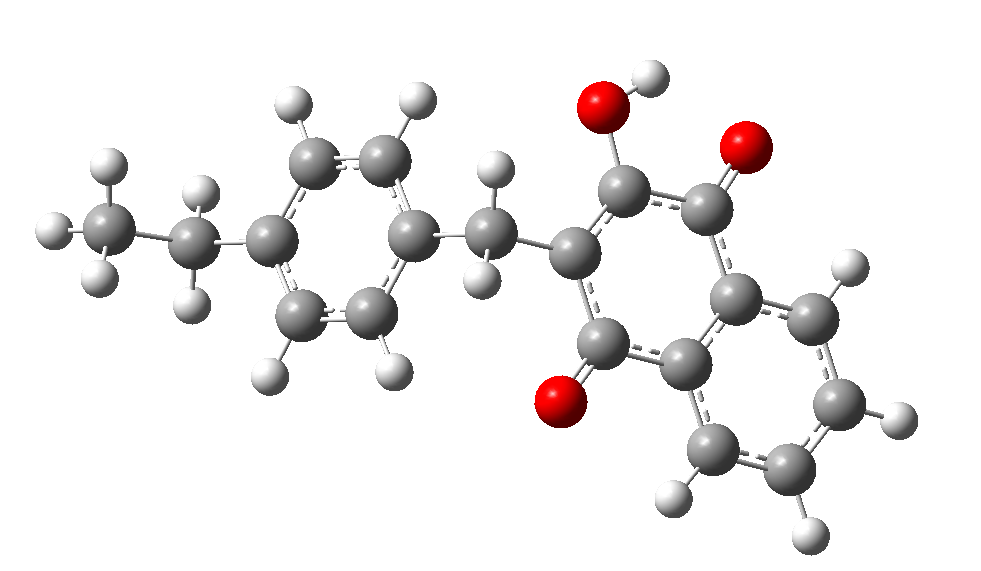 | | |
| --- | --- | --- | --- | --- | --- |
| Center  Number | Atom | Standard orientation: Coordinates (Angstroms) | | | |
|  |  | X | | Y | Z |
| 1 | C | -5.04501009 | | 1.02861314 | -1.26470914 |
| 2 | C | -4.37306699 | | -0.18869404 | -1.17193394 |
| 3 | C | -3.17741737 | | -0.26596466 | -0.45080517 |
| 4 | C | -2.64984094 | | 0.87512047 | 0.18150414 |
| 5 | C | -3.33040343 | | 2.08889052 | 0.08314635 |
| 6 | C | -4.52324813 | | 2.16431523 | -0.63786438 |
| 7 | H | -5.9732632 | | 1.09405229 | -1.82380113 |
| 8 | H | -4.75526332 | | -1.08558924 | -1.64762612 |
| 9 | C | -2.45311339 | | -1.54329102 | -0.34116179 |
| 10 | C | -1.367107 | | 0.80680913 | 0.9611559 |
| 11 | H | -2.9089845 | | 2.95753581 | 0.57701214 |
| 12 | H | -5.04845408 | | 3.11187244 | -0.71183199 |
| 13 | C | -0.65435303 | | -0.48235704 | 1.05531803 |
| 14 | C | -1.17708864 | | -1.5700952 | 0.4322352 |
| 15 | O | -0.9329951 | | 1.81557141 | 1.5147192 |
| 16 | O | -2.83190601 | | -2.60096202 | -0.84758259 |
| 17 | C | 0.64616655 | | -0.53570965 | 1.82376996 |
| 18 | H | 0.58954537 | | 0.1963463 | 2.63344951 |
| 19 | H | 0.75291234 | | -1.52995544 | 2.26644303 |
| 20 | O | -0.59656587 | | -2.77999694 | 0.46473663 |
| 21 | H | -1.19565181 | | -3.36122295 | -0.05068555 |
| 22 | C | 1.85849511 | | -0.23518839 | 0.9508756 |
| 23 | C | 2.27260825 | | 1.08392872 | 0.72764907 |
| 24 | C | 2.57247988 | | -1.27030879 | 0.3354092 |
| 25 | C | 3.37465157 | | 1.35508126 | -0.08197549 |
| 26 | H | 1.71822987 | | 1.89812893 | 1.18506867 |
| 27 | C | 3.67386615 | | -0.993188 | -0.47372601 |
| 28 | H | 2.25744892 | | -2.29915829 | 0.48636355 |
| 29 | C | 4.09704747 | | 0.32336 | -0.69574212 |
| 30 | H | 3.67857964 | | 2.38729657 | -0.24159043 |
| 31 | H | 4.21367906 | | -1.81368687 | -0.94137159 |
| 32 | C | 5.32028461 | | 0.61964005 | -1.53734792 |
| 33 | H | 5.41559145 | | -0.13865432 | -2.3238814 |
| 34 | H | 5.18881119 | | 1.58149301 | -2.04754266 |
| 35 | C | 6.61993732 | | 0.65893417 | -0.71397703 |
| 36 | H | 6.79716011 | | -0.2998536 | -0.21611062 |
| 37 | H | 7.48343484 | | 0.87567612 | -1.35183499 |
| 38 | H | 6.56863248 | | 1.42944411 | 0.06203091 |

**Table S11.** Optimized structure for compound **11** and cartesian Z-matrix.

| **(11)** | | | 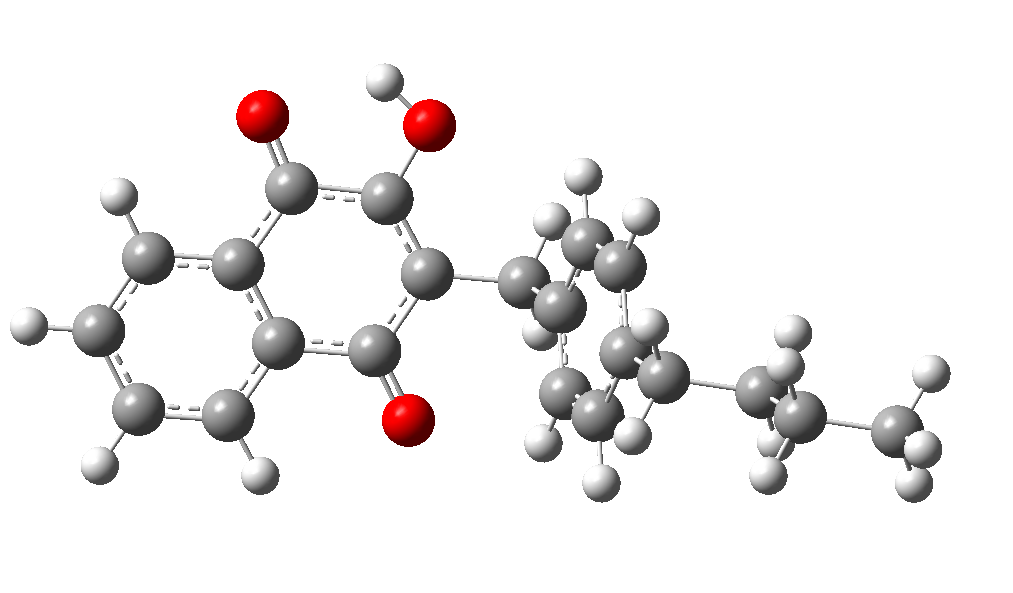 | | |
| --- | --- | --- | --- | --- | --- |
| Center  Number | Atom | Standard orientation: Coordinates (Angstroms) | | | |
|  |  | X | | Y | Z |
| 1 | C | 5.74290488 | | 1.29300259 | 1.38779152 |
| 2 | C | 5.13681584 | | 0.03963983 | 1.32705189 |
| 3 | C | 3.98453369 | | -0.1351295 | 0.55420557 |
| 4 | C | 3.43458092 | | 0.94413215 | -0.16200387 |
| 5 | C | 4.04910317 | | 2.19470675 | -0.09504271 |
| 6 | C | 5.19873138 | | 2.36746532 | 0.67758134 |
| 7 | H | 6.63728428 | | 1.43408581 | 1.98670909 |
| 8 | H | 5.53769894 | | -0.81140624 | 1.86722551 |
| 9 | C | 3.3300736 | | -1.45194622 | 0.4772526 |
| 10 | C | 2.19803899 | | 0.77082107 | -0.99800698 |
| 11 | H | 3.61132744 | | 3.0146471 | -0.65369662 |
| 12 | H | 5.67256129 | | 3.3432965 | 0.72674851 |
| 13 | C | 1.55524574 | | -0.5565403 | -1.05881254 |
| 14 | C | 2.09789881 | | -1.58316986 | -0.35438705 |
| 15 | O | 1.74366562 | | 1.72689996 | -1.62405141 |
| 16 | O | 3.73379561 | | -2.46192077 | 1.05652222 |
| 17 | C | 0.30026922 | | -0.71554453 | -1.88610859 |
| 18 | H | 0.36241878 | | -0.02289114 | -2.72935169 |
| 19 | H | 0.26562163 | | -1.73485972 | -2.28064825 |
| 20 | O | 1.57950473 | | -2.82143645 | -0.35093544 |
| 21 | H | 2.17921361 | | -3.34455411 | 0.22268467 |
| 22 | C | -0.96883519 | | -0.43507082 | -1.09066863 |
| 23 | C | -1.46002569 | | 0.86991757 | -0.95979638 |
| 24 | C | -1.66032484 | | -1.47348153 | -0.4553567 |
| 25 | C | -2.61446387 | | 1.12357459 | -0.22060073 |
| 26 | H | -0.92483859 | | 1.68774352 | -1.43333733 |
| 27 | C | -2.81445126 | | -1.21391376 | 0.28294038 |
| 28 | H | -1.28634019 | | -2.49052363 | -0.53476228 |
| 29 | C | -3.31448245 | | 0.08805082 | 0.41260349 |
| 30 | H | -2.97785517 | | 2.14501916 | -0.13196342 |
| 31 | H | -3.33540686 | | -2.03659265 | 0.76776111 |
| 32 | C | -4.5922345 | | 0.36175828 | 1.17581194 |
| 33 | H | -4.69240165 | | -0.35994841 | 1.99664006 |
| 34 | H | -4.53758221 | | 1.35451107 | 1.64072721 |
| 35 | C | -5.85376614 | | 0.29253206 | 0.29255975 |
| 36 | H | -5.90862129 | | -0.69893316 | -0.17682467 |
| 37 | H | -5.75420244 | | 1.01183238 | -0.53143377 |
| 38 | C | -7.14939959 | | 0.56938939 | 1.0640291 |
| 39 | H | -7.24095365 | | -0.15105991 | 1.88839449 |
| 40 | H | -7.08606527 | | 1.56024084 | 1.5343395 |
| 41 | C | -8.40093119 | | 0.50056695 | 0.18376093 |
| 42 | H | -8.5101075 | | -0.48967038 | -0.27281322 |
| 43 | H | -9.30888193 | | 0.70203122 | 0.76152562 |
| 44 | H | -8.35428617 | | 1.23412483 | -0.62903344 |

**Table S12.** Optimized structure for compound **12** and cartesian Z-matrix.

| **(12)** | | | 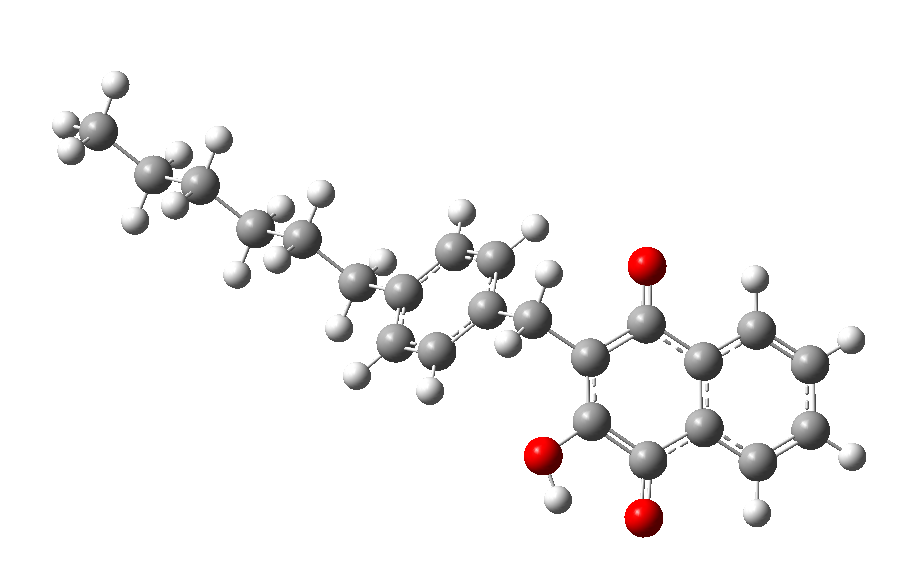 | | |
| --- | --- | --- | --- | --- | --- |
| Center  Number | Atom | Standard orientation: Coordinates (Angstroms) | | | |
|  |  | X | | Y | Z |
| 1 | C | 6.58516683 | | 1.44779429 | -1.42172373 |
| 2 | C | 6.01592637 | | 0.17650545 | -1.37969383 |
| 3 | C | 4.88199623 | | -0.04829898 | -0.59278711 |
| 4 | C | 4.31359922 | | 0.99862467 | 0.1562748 |
| 5 | C | 4.89125605 | | 2.26749189 | 0.10783805 |
| 6 | C | 6.02260543 | | 2.49021355 | -0.67884784 |
| 7 | H | 7.46516579 | | 1.62770354 | -2.03144245 |
| 8 | H | 6.43181047 | | -0.65051885 | -1.94524413 |
| 9 | C | 4.26646595 | | -1.38475579 | -0.53507128 |
| 10 | C | 3.09637916 | | 0.77151616 | 1.00761544 |
| 11 | H | 4.43972611 | | 3.06197333 | 0.69176079 |
| 12 | H | 6.46774653 | | 3.48007852 | -0.71344112 |
| 13 | C | 2.49263543 | | -0.57476521 | 1.04881707 |
| 14 | C | 3.05251794 | | -1.56960619 | 0.31294476 |
| 15 | O | 2.6253731 | | 1.70022927 | 1.66194023 |
| 16 | O | 4.68917946 | | -2.3697338 | -1.14313997 |
| 17 | C | 1.25665867 | | -0.78813179 | 1.89234979 |
| 18 | H | 1.25918002 | | -1.81561261 | 2.26678632 |
| 19 | H | 1.31183825 | | -0.11069164 | 2.74834598 |
| 20 | O | 2.56953188 | | -2.82185621 | 0.2901299 |
| 21 | H | 3.17418793 | | -3.3147425 | -0.30469992 |
| 22 | C | -0.0332528 | | -0.5309205 | 1.12302374 |
| 23 | C | -0.70710751 | | -1.5782354 | 0.48353652 |
| 24 | C | -0.56219899 | | 0.76189835 | 1.02092413 |
| 25 | C | -1.88072876 | | -1.33950439 | -0.23076658 |
| 26 | H | -0.30418309 | | -2.58567977 | 0.54063736 |
| 27 | C | -1.73580745 | | 0.99474007 | 0.30555329 |
| 28 | H | -0.04150091 | | 1.58690107 | 1.49818497 |
| 29 | C | -2.41829929 | | -0.05006889 | -0.33167051 |
| 30 | H | -2.38743125 | | -2.16884331 | -0.71932722 |
| 31 | H | -2.1284614 | | 2.00699536 | 0.23899814 |
| 32 | C | -3.71619701 | | 0.19997118 | -1.06854618 |
| 33 | H | -3.69436852 | | 1.19841034 | -1.52386658 |
| 34 | H | -3.81394176 | | -0.51561971 | -1.89495004 |
| 35 | C | -4.95899771 | | 0.0909755 | -0.16273034 |
| 36 | H | -4.86058631 | | 0.80440352 | 0.6663271 |
| 37 | H | -4.97965048 | | -0.90602346 | 0.29728802 |
| 38 | C | -6.27489015 | | 0.34333845 | -0.90769343 |
| 39 | H | -6.24498238 | | 1.34019267 | -1.37069372 |
| 40 | H | -6.36556521 | | -0.37207186 | -1.73772858 |
| 41 | C | -7.51466228 | | 0.23856044 | -0.01123012 |
| 42 | H | -7.42480629 | | 0.95456364 | 0.81822465 |
| 43 | H | -7.54460524 | | -0.75778773 | 0.45262673 |
| 44 | C | -8.83282868 | | 0.48935216 | -0.7540169 |
| 45 | H | -8.80268034 | | 1.48489038 | -1.21752384 |
| 46 | H | -8.92285818 | | -0.22680236 | -1.58218479 |
| 47 | C | -10.06574377 | | 0.38366629 | 0.14847375 |
| 48 | H | -10.02176703 | | 1.11192858 | 0.96630802 |
| 49 | H | -10.98913849 | | 0.56746384 | -0.41041297 |
| 50 | H | -10.14276113 | | -0.61239297 | 0.59914517 |

**Table S13.** Optimized structure for compound **13** and cartesian Z-matrix.

| **13** | | | 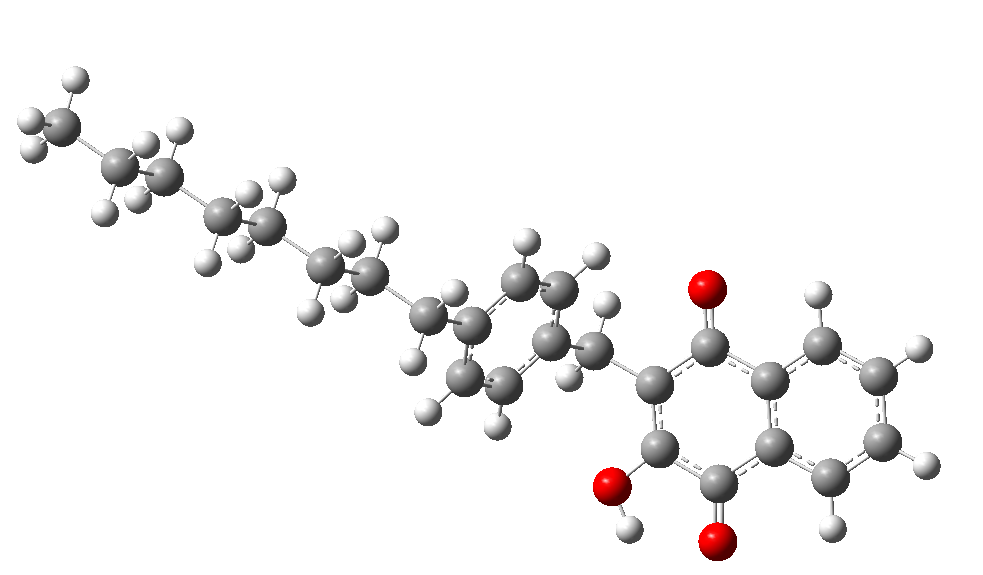 | | |
| --- | --- | --- | --- | --- | --- |
| Center  Number | Atom | Standard orientation: Coordinates (Angstroms) | | | |
|  |  | X | | Y | Z |
| 1 | C | 7.52045015 | | 1.53898606 | 1.42069602 |
| 2 | C | 6.9728523 | | 0.25797622 | 1.38704435 |
| 3 | C | 5.84510118 | | 0.00796752 | 0.59886635 |
| 4 | C | 5.26124248 | | 1.03931262 | -0.15985123 |
| 5 | C | 5.81726973 | | 2.31809615 | -0.11973815 |
| 6 | C | 6.94247911 | | 2.56597816 | 0.66822514 |
| 7 | H | 8.39557692 | | 1.73844167 | 2.03134597 |
| 8 | H | 7.40104877 | | -0.55749441 | 1.96013553 |
| 9 | C | 5.25235238 | | -1.33910363 | 0.54989194 |
| 10 | C | 4.0504534 | | 0.78509427 | -1.01268237 |
| 11 | H | 5.35405782 | | 3.10028995 | -0.71105347 |
| 12 | H | 7.37076036 | | 3.56345835 | 0.69630148 |
| 13 | C | 3.46969127 | | -0.57148839 | -1.04516331 |
| 14 | C | 4.04415977 | | -1.55099261 | -0.30000611 |
| 15 | O | 3.56566381 | | 1.70069933 | -1.67538513 |
| 16 | O | 5.68986745 | | -2.31205512 | 1.16678325 |
| 17 | C | 2.24001111 | | -0.81231665 | -1.89052555 |
| 18 | H | 2.28573766 | | -0.1398448 | -2.75098754 |
| 19 | H | 2.26163254 | | -1.84212164 | -2.25789383 |
| 20 | O | 3.58235105 | | -2.81100509 | -0.2688686 |
| 21 | H | 4.19346206 | | -3.2889974 | 0.33148881 |
| 22 | C | 0.94357986 | | -0.5726489 | -1.12649594 |
| 23 | C | 0.39371809 | | 0.71198119 | -1.03149717 |
| 24 | C | 0.28439415 | | -1.62796207 | -0.48490691 |
| 25 | C | -0.78585707 | | 0.92895473 | -0.32097979 |
| 26 | H | 0.90271088 | | 1.54318949 | -1.51061826 |
| 27 | C | -0.89531733 | | -1.40509793 | 0.2244907 |
| 28 | H | 0.70350231 | | -2.62907958 | -0.53651737 |
| 29 | C | -1.453746 | | -0.12402361 | 0.31828249 |
| 30 | H | -1.194862 | | 1.93506555 | -0.25993299 |
| 31 | H | -1.39038008 | | -2.24042487 | 0.71477851 |
| 32 | C | -2.75797754 | | 0.10820537 | 1.04978667 |
| 33 | H | -2.84747002 | | -0.60608562 | 1.87824453 |
| 34 | H | -2.75333002 | | 1.10836264 | 1.5018239 |
| 35 | C | -3.995728 | | -0.02312116 | 0.14000968 |
| 36 | H | -3.99892778 | | -1.02162074 | -0.31720516 |
| 37 | H | -3.90576404 | | 0.68943268 | -0.69076568 |
| 38 | C | -5.31806683 | | 0.21036065 | 0.879712 |
| 39 | H | -5.40060398 | | -0.504519 | 1.71101693 |
| 40 | H | -5.30544357 | | 1.2085603 | 1.3405397 |
| 41 | C | -6.55270027 | | 0.08420273 | -0.02136871 |
| 42 | H | -6.56383379 | | -0.91317913 | -0.48350891 |
| 43 | H | -6.47018365 | | 0.80023585 | -0.85137895 |
| 44 | C | -7.8771489 | | 0.31497146 | 0.71664798 |
| 45 | H | -7.95989931 | | -0.40212669 | 1.54594766 |
| 46 | H | -7.86514325 | | 1.31188934 | 1.1801451 |
| 47 | C | -9.11189019 | | 0.19104166 | -0.18417763 |
| 48 | H | -9.12453049 | | -0.80552034 | -0.6486126 |
| 49 | H | -9.03047479 | | 0.90870806 | -1.01320907 |
| 50 | C | -10.43677841 | | 0.42058934 | 0.5533845 |
| 51 | H | -10.51858588 | | -0.29709986 | 1.38108087 |
| 52 | H | -10.42425675 | | 1.41633674 | 1.01726755 |
| 53 | C | -11.66452165 | | 0.29558603 | -0.35368673 |
| 54 | H | -11.72409113 | | -0.70148807 | -0.8048013 |
| 55 | H | -12.59286476 | | 0.4646214 | 0.20167304 |
| 54 | H | -11.62903936 | | 1.02460188 | -1.17128987 |

**Table S14.** Optimized structure for ciprofloxacin and cartesian Z-matrix.

| D1 | | | 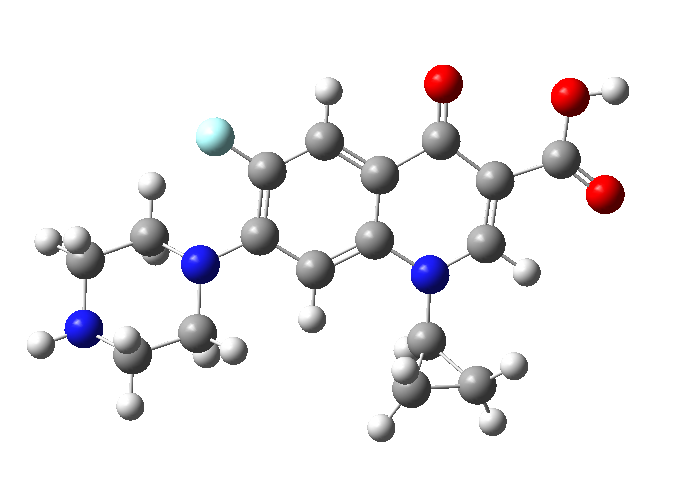 | | |
| --- | --- | --- | --- | --- | --- |
| Center  Number | Atom | Standard orientation: Coordinates (Angstroms) | | | |
|  |  | X | | Y | Z |
| 1 | F | -2.12866256 | | -2.79060195 | -0.45538353 |
| 2 | O | 2.85016304 | | -2.6442111 | -0.30804752 |
| 3 | O | 5.39803043 | | -1.66402277 | 0.02337385 |
| 4 | O | 5.61991345 | | 0.54915793 | 0.32113149 |
| 5 | N | 1.59397909 | | 1.24744874 | 0.2136814 |
| 6 | N | -3.08575375 | | -0.21071086 | -0.06181648 |
| 7 | N | -5.79737066 | | 0.52599424 | 0.36364745 |
| 8 | C | 1.18669758 | | 2.6179344 | 0.45221196 |
| 9 | C | 1.96056348 | | 3.74508564 | -0.17632413 |
| 10 | C | 0.57390476 | | 3.42297279 | -0.66823321 |
| 11 | C | 0.63016995 | | 0.23106459 | 0.06327043 |
| 12 | C | 2.91005761 | | 0.92099816 | 0.25172464 |
| 13 | C | 1.07180188 | | -1.09389112 | -0.11006826 |
| 14 | C | -0.74368206 | | 0.52468833 | 0.11039822 |
| 15 | C | -1.7113872 | | -0.47669126 | -0.04365781 |
| 16 | C | -3.94345971 | | -0.97045709 | 0.86913084 |
| 17 | C | -3.51308786 | | 1.18223799 | -0.19182182 |
| 18 | C | 3.41685116 | | -0.3428405 | 0.08781072 |
| 19 | C | 2.50876217 | | -1.47622107 | -0.1309043 |
| 20 | C | -5.40286082 | | -0.88090417 | 0.43218411 |
| 21 | C | -4.98173569 | | 1.23937637 | -0.61572061 |
| 22 | C | 0.10623497 | | -2.10082781 | -0.26829547 |
| 23 | C | -1.23081573 | | -1.79538317 | -0.25051984 |
| 24 | C | 4.89517974 | | -0.42219252 | 0.15596169 |
| 25 | H | 0.79338419 | | 2.78268602 | 1.45367892 |
| 26 | H | 2.79015115 | | 3.49673711 | -0.82953714 |
| 27 | H | 2.11457958 | | 4.63547249 | 0.42405375 |
| 28 | H | -0.24404171 | | 4.09313254 | -0.42274754 |
| 29 | H | 0.49168333 | | 2.93810821 | -1.63535362 |
| 30 | H | 3.5989381 | | 1.73941471 | 0.42003902 |
| 31 | H | -1.06186949 | | 1.54111489 | 0.28643103 |
| 32 | H | -3.84746496 | | -0.55979028 | 1.88769928 |
| 33 | H | -3.62434631 | | -2.0105051 | 0.88269222 |
| 34 | H | -2.89954099 | | 1.66814031 | -0.95587362 |
| 35 | H | -3.39252698 | | 1.73695403 | 0.75533517 |
| 36 | H | -5.51203612 | | -1.40791516 | -0.53327926 |
| 37 | H | -6.02886285 | | -1.39308802 | 1.17053397 |
| 38 | H | -5.30056779 | | 2.28662965 | -0.65502194 |
| 39 | H | -5.06351239 | | 0.81995316 | -1.63556988 |
| 40 | H | 0.44317886 | | -3.11881579 | -0.42784645 |
| 41 | H | -6.78078731 | | 0.60245395 | 0.11956661 |
| 42 | H | 6.36160077 | | -1.55147988 | 0.09051918 |

**Table S15.** Optimized structure for ofloxacin and cartesian Z-matrix.

| **D2** | | | 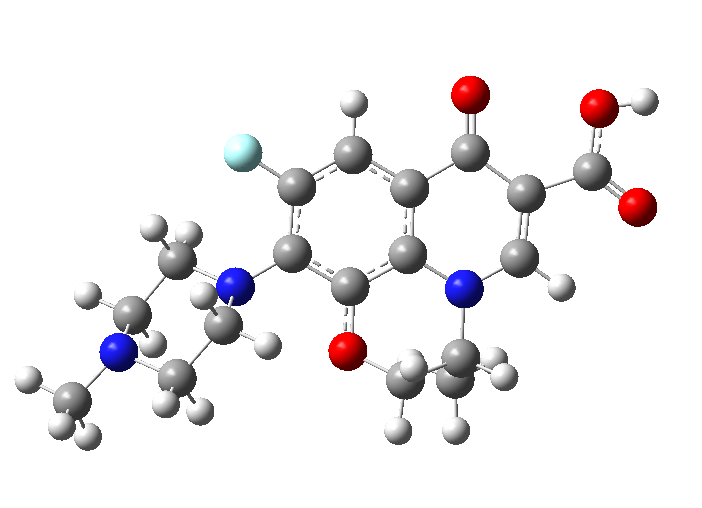 | | |
| --- | --- | --- | --- | --- | --- |
| Center  Number | Atom | Standard orientation: Coordinates (Angstroms) | | | |
|  |  | X | | Y | Z |
| 1 | F | -1.48430139 | | -3.05170974 | 0.000279 |
| 2 | O | -0.88447851 | | 1.66494474 | -0.1667287 |
| 3 | O | 3.48632352 | | -2.49640834 | 0.04822345 |
| 4 | O | 5.94329568 | | -1.2673825 | -0.03648046 |
| 5 | O | 5.97050015 | | 0.97649526 | -0.08588429 |
| 6 | N | -2.63406882 | | -0.45294004 | -0.05280398 |
| 7 | N | 1.90438386 | | 1.2981572 | -0.09222021 |
| 8 | N | -5.45098711 | | -0.02806267 | -0.1286424 |
| 9 | C | -3.54063442 | | -1.37547179 | 0.63321745 |
| 10 | C | -3.26002128 | | 0.17292616 | -1.22222879 |
| 11 | C | 1.33317005 | | 2.65248443 | 0.02391011 |
| 12 | C | -1.25426842 | | -0.68891213 | -0.07164103 |
| 13 | C | 1.03771945 | | 0.19567627 | -0.08954165 |
| 14 | C | -0.35525461 | | 0.39651622 | -0.12012586 |
| 15 | C | -4.80530593 | | -0.61967169 | 1.04136916 |
| 16 | C | -4.54004548 | | 0.89296346 | -0.80414511 |
| 17 | C | -0.00100645 | | 2.63994982 | -0.72149358 |
| 18 | C | 1.57440719 | | -1.10339893 | -0.02191519 |
| 19 | C | -0.66517526 | | -1.9731538 | -0.00589143 |
| 20 | C | 3.24065858 | | 1.10546282 | -0.07375997 |
| 21 | C | 0.69511328 | | -2.18974142 | 0.02393561 |
| 22 | C | 1.18978284 | | 3.0851798 | 1.48720005 |
| 23 | C | 3.04408177 | | -1.35114709 | -0.00387808 |
| 24 | C | -6.71440213 | | 0.60816602 | 0.21012039 |
| 25 | C | 3.85333913 | | -0.12392506 | -0.05068172 |
| 26 | C | 5.33362443 | | -0.06767654 | -0.05928196 |
| 27 | H | -3.82115218 | | -2.22619709 | -0.0041176 |
| 28 | H | -3.04489341 | | -1.76818906 | 1.52354526 |
| 29 | H | -2.56591436 | | 0.88970344 | -1.66111802 |
| 30 | H | -3.50282832 | | -0.58869436 | -1.9820618 |
| 31 | H | 2.01338017 | | 3.33270501 | -0.50047361 |
| 32 | H | -5.50473675 | | -1.32274238 | 1.50858628 |
| 33 | H | -4.54325292 | | 0.14650342 | 1.79778855 |
| 34 | H | -4.26969701 | | 1.75229278 | -0.15913121 |
| 35 | H | -5.0392594 | | 1.28876685 | -1.69650901 |
| 36 | H | -0.50921307 | | 3.60069636 | -0.62046359 |
| 37 | H | 0.16383953 | | 2.42984578 | -1.78666204 |
| 38 | H | 3.85643959 | | 1.99926199 | -0.08029584 |
| 39 | H | 1.10150703 | | -3.19355148 | 0.05893897 |
| 40 | H | 2.15287359 | | 3.03281665 | 2.00153454 |
| 41 | H | 0.82880125 | | 4.1172343 | 1.53942386 |
| 42 | H | 0.47754896 | | 2.44513414 | 2.01356066 |
| 43 | H | -6.60462166 | | 1.4528874 | 0.91754562 |
| 44 | H | -7.38800317 | | -0.12605495 | 0.66361252 |
| 45 | H | -7.19161957 | | 0.9892922 | -0.69861503 |
| 46 | H | 6.89439531 | | -1.06495736 | -0.04598364 |

**Table S16.** Optimized structure for vancomycin and cartesian Z-matrix.

| D3 | | | 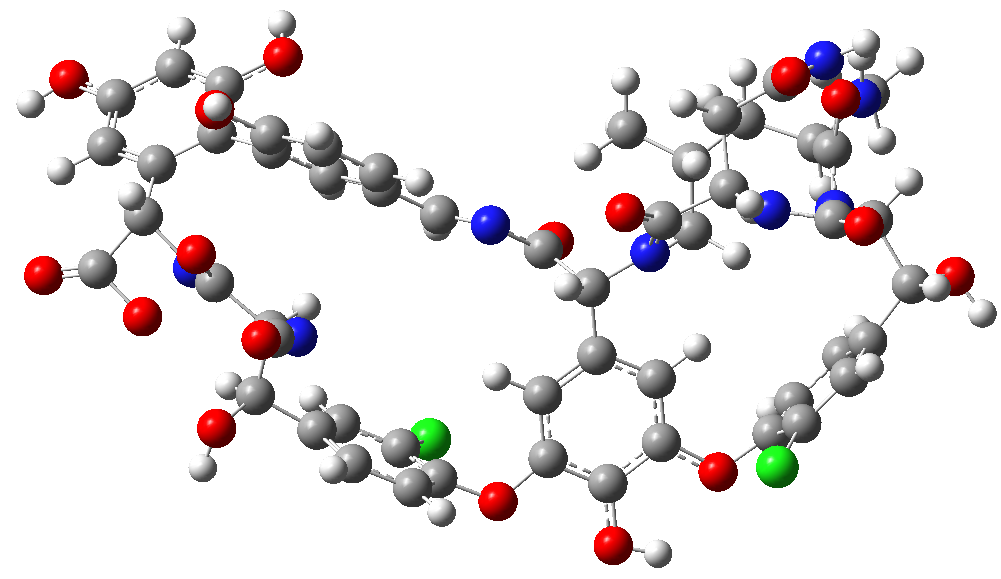 | | |
| --- | --- | --- | --- | --- | --- |
| Center  Number | Atom | Standard orientation: Coordinates (Angstroms) | | | |
|  |  | X | | Y | Z |
| 1 | N | 7.25218115 | | 4.41912203 | -2.31698503 |
| 2 | C | 7.52044026 | | 4.88656296 | -3.65738809 |
| 3 | C | 6.28382976 | | 3.37789245 | -2.09958274 |
| 4 | C | 6.51223737 | | 2.8625394 | -0.66476353 |
| 5 | O | 6.63310674 | | 3.7105947 | 0.2428841 |
| 6 | C | 4.81478216 | | 3.89667504 | -2.26069806 |
| 7 | C | 3.65876372 | | 2.9070603 | -2.03874426 |
| 8 | C | 3.7409806 | | 1.66033792 | -2.92943596 |
| 9 | C | 2.32488809 | | 3.64312061 | -2.24925194 |
| 10 | N | 6.55391473 | | 1.52450258 | -0.54478345 |
| 11 | C | 7.28946661 | | 1.01798537 | 0.55179285 |
| 12 | C | 6.37279768 | | 0.67429536 | 1.81770641 |
| 13 | O | 6.92642213 | | 0.26424533 | 2.82609284 |
| 14 | C | 8.1023633 | | -0.26563598 | 0.19085662 |
| 15 | O | 9.082675 | | 0.16930344 | -0.74373769 |
| 16 | C | 7.22309757 | | -1.37305518 | -0.35006139 |
| 17 | C | 6.7709734 | | -2.38454083 | 0.50354793 |
| 18 | C | 6.77472918 | | -1.33895088 | -1.67642006 |
| 19 | C | 5.80537822 | | -3.28998972 | 0.06688692 |
| 20 | Cl | 5.21388903 | | -4.52685541 | 1.15305433 |
| 21 | C | 5.79686069 | | -2.2292194 | -2.10681389 |
| 22 | C | 5.27417114 | | -3.16947387 | -1.22120082 |
| 23 | O | 4.16648189 | | -3.91232396 | -1.60846514 |
| 24 | N | 5.05994056 | | 0.90107538 | 1.65265001 |
| 25 | C | 4.10256054 | | 1.0484801 | 2.74447338 |
| 26 | C | 2.73607071 | | 0.44165932 | 2.38087497 |
| 27 | O | 1.88669511 | | 0.35213173 | 3.28269968 |
| 28 | C | 3.93667175 | | 2.53241699 | 3.15394348 |
| 29 | C | 5.14302288 | | 3.05445429 | 3.93845728 |
| 30 | O | 5.23901204 | | 2.86162439 | 5.14399101 |
| 31 | N | 6.07691373 | | 3.73658726 | 3.21793812 |
| 32 | N | 2.56187532 | | 0.08791917 | 1.08423101 |
| 33 | C | 1.33661414 | | -0.61266034 | 0.81742343 |
| 34 | C | 0.39982702 | | 0.40471239 | 0.04394642 |
| 35 | O | 0.70527999 | | 0.87534978 | -1.03126244 |
| 36 | C | 1.52135328 | | -1.84965414 | -0.0371665 |
| 37 | C | 0.40657995 | | -2.64326133 | -0.35894922 |
| 38 | C | 0.56625512 | | -3.81933017 | -1.08204932 |
| 39 | C | 1.84826418 | | -4.22743752 | -1.50012814 |
| 40 | O | 2.00398168 | | -5.39095507 | -2.16636891 |
| 41 | C | 2.94781498 | | -3.41053246 | -1.17523993 |
| 42 | C | 2.79342411 | | -2.22826087 | -0.4658571 |
| 43 | N | 0.75679308 | | 0.71680481 | 0.77715388 |
| 44 | C | 1.78230865 | | 1.1084976 | 0.09379434 |
| 45 | C | 5.46772313 | | -2.49793442 | 3.36436721 |
| 46 | O | 5.83221788 | | -2.83282046 | 4.42953432 |
| 47 | C | 3.02865166 | | 1.51333228 | 0.72027122 |
| 48 | C | 4.09063764 | | 1.97566331 | -0.07539882 |
| 49 | C | 5.3146738 | | 2.37195326 | 0.46914315 |
| 50 | C | 5.455464 | | 2.2994738 | 1.87111575 |
| 51 | O | 6.64524247 | | 2.68245508 | 2.4014561 |
| 52 | C | 4.40413729 | | 1.83532147 | 2.68264946 |
| 53 | C | 3.20241649 | | 1.45061371 | 2.11874448 |
| 54 | N | 4.91297208 | | -2.18969101 | 2.32675971 |
| 55 | C | 5.38925313 | | -1.70288719 | 1.06906849 |
| 56 | O | 0.46159858 | | -4.67920445 | -1.41242243 |
| 57 | C | 2.35608817 | | -4.80054016 | 0.04508651 |
| 58 | C | 2.46067724 | | -3.41417928 | -1.93013112 |
| 59 | C | 1.74588903 | | -4.28862981 | -1.10076191 |
| 60 | C | 4.32761755 | | -3.4675753 | -0.38567521 |
| 61 | C | 3.64047056 | | -4.39739634 | 0.40210608 |
| 62 | C | 3.74223884 | | -3.0051512 | -1.56844317 |
| 63 | C | 5.64734431 | | -2.86189136 | 0.05933664 |
| 64 | Cl | 1.74656894 | | -2.8176337 | -3.41023511 |
| 65 | O | 7.03790665 | | -0.430154 | 2.3074398 |
| 66 | C | 6.57621575 | | -0.75057087 | 1.23917172 |
| 67 | O | 6.5103028 | | -3.76418575 | 0.72733801 |
| 68 | N | 6.95185931 | | -0.09964162 | -0.0061688 |
| 69 | O | 4.98005413 | | 4.73798513 | -0.89075354 |
| 70 | C | 6.19280772 | | 4.1378391 | -1.06529276 |
| 71 | C | 6.40581855 | | 2.91338167 | -0.39438764 |
| 72 | C | 7.17577648 | | 4.72070866 | -1.86343602 |
| 73 | C | 8.40656338 | | 4.08385701 | -2.01079107 |
| 74 | O | 9.33369933 | | 4.69349262 | -2.79901406 |
| 75 | C | 8.64570578 | | 2.86450346 | -1.37313971 |
| 76 | C | 7.65274383 | | 2.28680724 | -0.57662531 |
| 77 | C | 7.98849663 | | 0.97411602 | 0.10358224 |
| 78 | C | 8.8997388 | | 0.01618115 | -0.65205692 |
| 79 | O | 7.95506348 | | -0.98956883 | -0.69305476 |
| 80 | O | 9.99072956 | | -0.01058737 | -1.13272584 |
| 81 | H | 4.74008319 | | 4.30590905 | -3.27769443 |
| 82 | H | 4.70809771 | | 4.73921687 | -1.56619172 |
| 83 | H | 3.68218052 | | 2.56920476 | -0.99351044 |
| 84 | H | 2.87690613 | | 1.01588503 | -2.74440958 |
| 85 | H | 4.64175764 | | 1.07566088 | -2.72086454 |
| 86 | H | 3.74285848 | | 1.93490023 | -3.99240829 |
| 87 | H | 1.48446666 | | 2.98202906 | -2.02656954 |
| 88 | H | 2.22797087 | | 3.98321645 | -3.28817195 |
| 89 | H | 2.24872286 | | 4.5249857 | -1.60292561 |
| 90 | H | 6.47037178 | | 2.56780218 | -2.81167661 |
| 91 | H | 7.20353638 | | 5.13482979 | -1.60068679 |
| 92 | H | 3.07517941 | | 2.59339467 | 3.82185643 |
| 93 | H | 3.7362547 | | 3.13397692 | 2.25977379 |
| 94 | H | 6.92048714 | | 3.98470455 | 3.71462418 |
| 95 | H | 6.11215577 | | 3.74161945 | 2.20300612 |
| 96 | H | 4.48183547 | | 0.49106476 | 3.6049328 |
| 97 | H | 4.75600211 | | 1.15218692 | 0.71723779 |
| 98 | H | 8.35444429 | | 5.59328202 | -3.63540625 |
| 99 | H | 7.80993773 | | 4.04237779 | -4.29276117 |
| 100 | H | 6.66386538 | | 5.38886773 | -4.14030161 |
| 101 | H | 8.00284184 | | 1.74656362 | 0.95920845 |
| 102 | H | 8.56587495 | | -0.58774279 | 1.13270555 |
| 103 | H | 9.67082731 | | -0.57858957 | -0.91509965 |
| 104 | H | 7.12799865 | | -2.44146831 | 1.52657445 |
| 105 | H | 7.15415102 | | -0.57130568 | -2.33924092 |
| 106 | H | 5.37654769 | | -2.16883364 | -3.10515885 |
| 107 | H | 0.79225343 | | -0.86165156 | 1.73810176 |
| 108 | H | 0.58106458 | | -2.35092347 | -0.02222585 |
| 109 | H | 2.95441632 | | -5.50981648 | -2.32528341 |
| 110 | H | 3.63693506 | | -1.60638114 | -0.2024616 |
| 111 | H | 1.74425929 | | 1.14028795 | -1.00281719 |
| 112 | H | 3.96181453 | | 2.03267925 | -1.15221214 |
| 113 | H | 6.65493462 | | 2.48632442 | 3.34865388 |
| 114 | H | 4.54548285 | | 1.78242583 | 3.75939783 |
| 115 | H | 2.38463415 | | 1.0907112 | 2.73344193 |
| 116 | H | 4.60835476 | | -1.07362802 | 0.62495237 |
| 117 | H | 1.79244887 | | -5.49499301 | 0.65901298 |
| 118 | H | 4.10278491 | | -4.77633809 | 1.30587691 |
| 119 | H | 4.27294385 | | -2.30867904 | -2.21051875 |
| 120 | H | 6.13720003 | | -2.42251246 | -0.81788933 |
| 121 | H | 6.7585126 | | -4.46167224 | 0.10637454 |
| 122 | H | 4.9670513 | | 5.57338977 | -1.37620383 |
| 123 | H | 7.00265593 | | 5.66597103 | -2.36940399 |
| 124 | H | 0.13568037 | | 4.15441144 | -2.82427369 |

**Table S17.** Variance in Principal Components of complexes of compounds **9**, **11**, **13**, and ofloxacin with 3E0M protein using PCA, at 300 K temperatures.

| Temperature (K) | Ligand | Principle Components | | | Cosine Content |
| --- | --- | --- | --- | --- | --- |
|  |  | PC1 (%) | PC2 (%) | PC3 (%) |  |
| **300** | 9 | 27.01 | 24.99 | 9.45 | 0.60 |
|  | 11 | 69.96 | 10.56 | 4.51 | 0.43 |
|  | 13 | 45.09 | 16.82 | 6.79 | 0.70 |
|  | Ofloxacin | 55.17 | 10.55 | 7.23 | 0.50 |

**
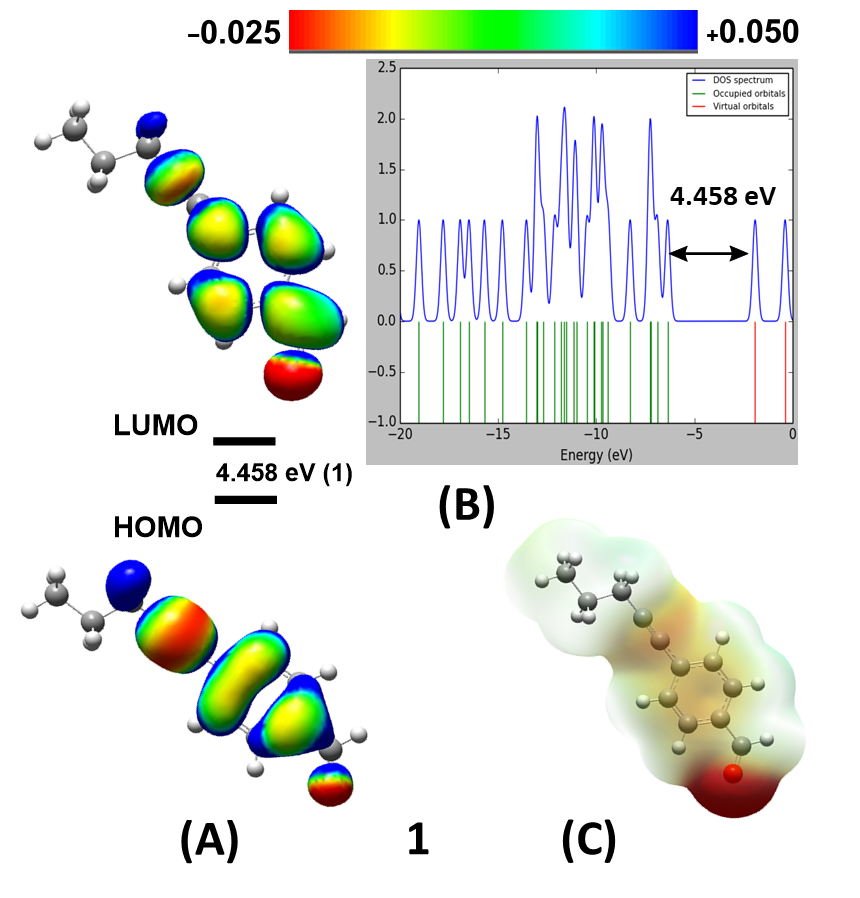
**

**Fig. S1.** (A) Molecular orbitals of isodensity surfaces (0.02 electrons Bohr−3 surface) (red = electron-rich, blue = electron-deficient) of HOMO and LUMO; (B) Maps of electrostatic potential (0.02 electrons Bohr−3 surface) (red = electron-rich, blue = electron-deficient); (C) DOS plot and HOMO-LUMO energy gap for the compound, **1**.

**
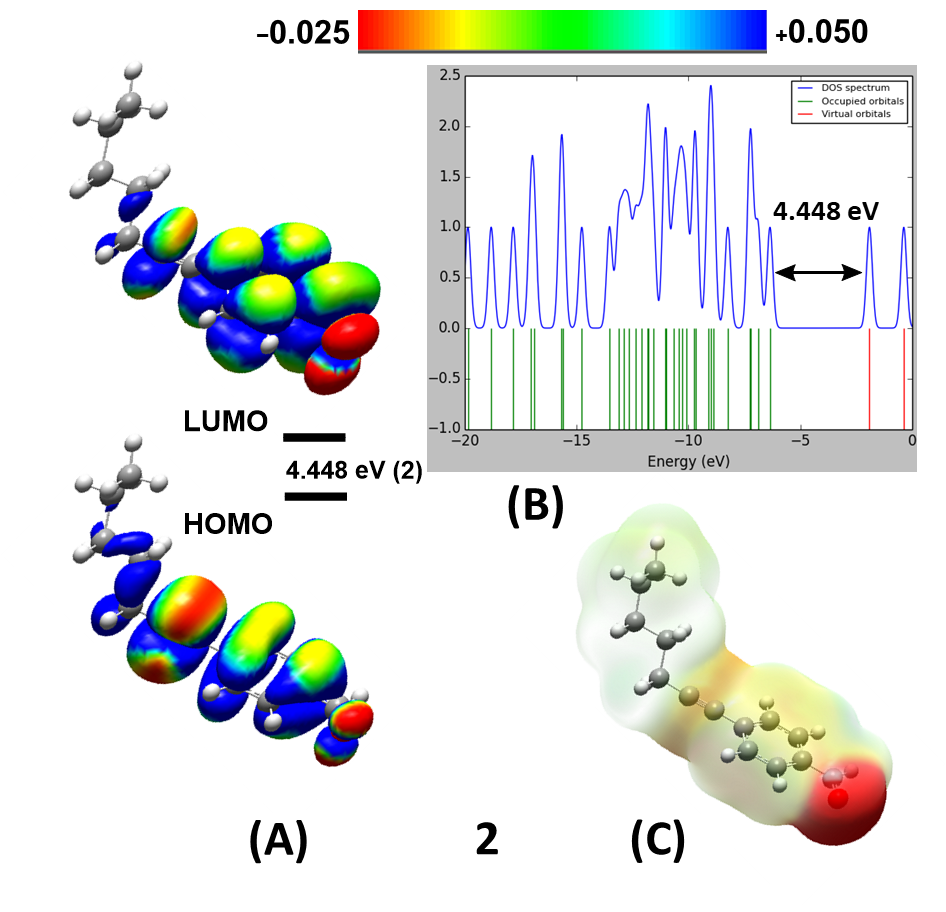
**

**Fig. S2.** (A) Molecular orbitals of isodensity surfaces (0.02 electrons Bohr−3 surface) (red = electron-rich, blue = electron-deficient) of HOMO and LUMO; (B) Maps of electrostatic potential (0.02 electrons Bohr−3 surface) (red = electron-rich, blue = electron-deficient); (C) DOS plot and HOMO-LUMO energy gap for the compound, **2**.


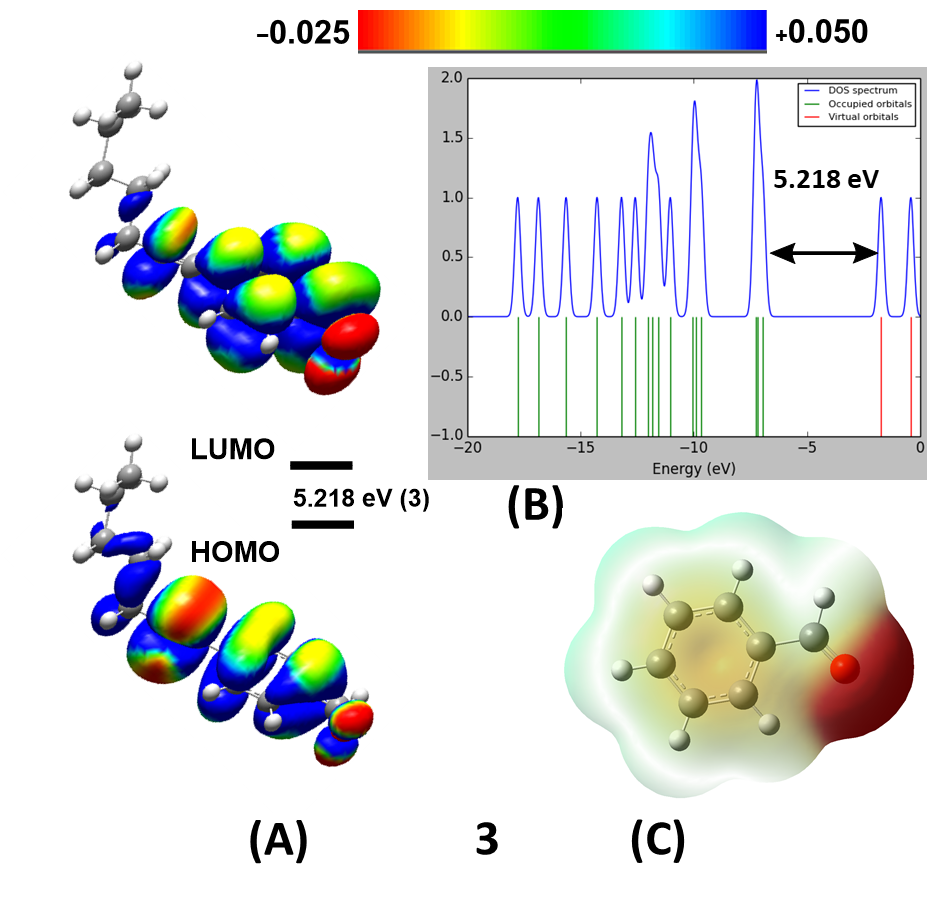


**Fig. S3.** (A) Molecular orbitals of isodensity surfaces (0.02 electrons Bohr−3 surface) (red = electron-rich, blue = electron-deficient) of HOMO and LUMO; (B) Maps of electrostatic potential (0.02 electrons Bohr−3 surface) (red = electron-rich, blue = electron-deficient); (C) DOS plot and HOMO-LUMO energy gap for the compound, **3.**


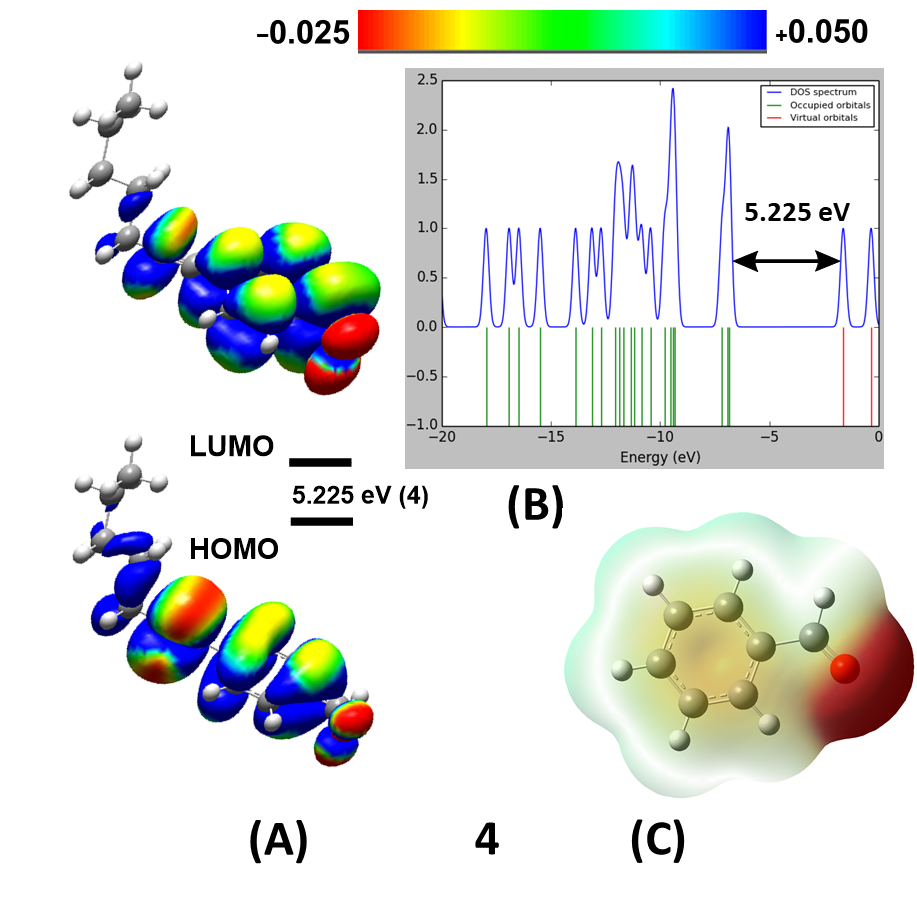


**Fig. S4.** (A) Molecular orbitals of isodensity surfaces (0.02 electrons Bohr−3 surface) (red = electron-rich, blue = electron-deficient) of HOMO and LUMO; (B) Maps of electrostatic potential (0.02 electrons Bohr−3 surface) (red = electron-rich, blue = electron-deficient); (C) DOS plot and HOMO-LUMO energy gap for the compound, **4.**

**
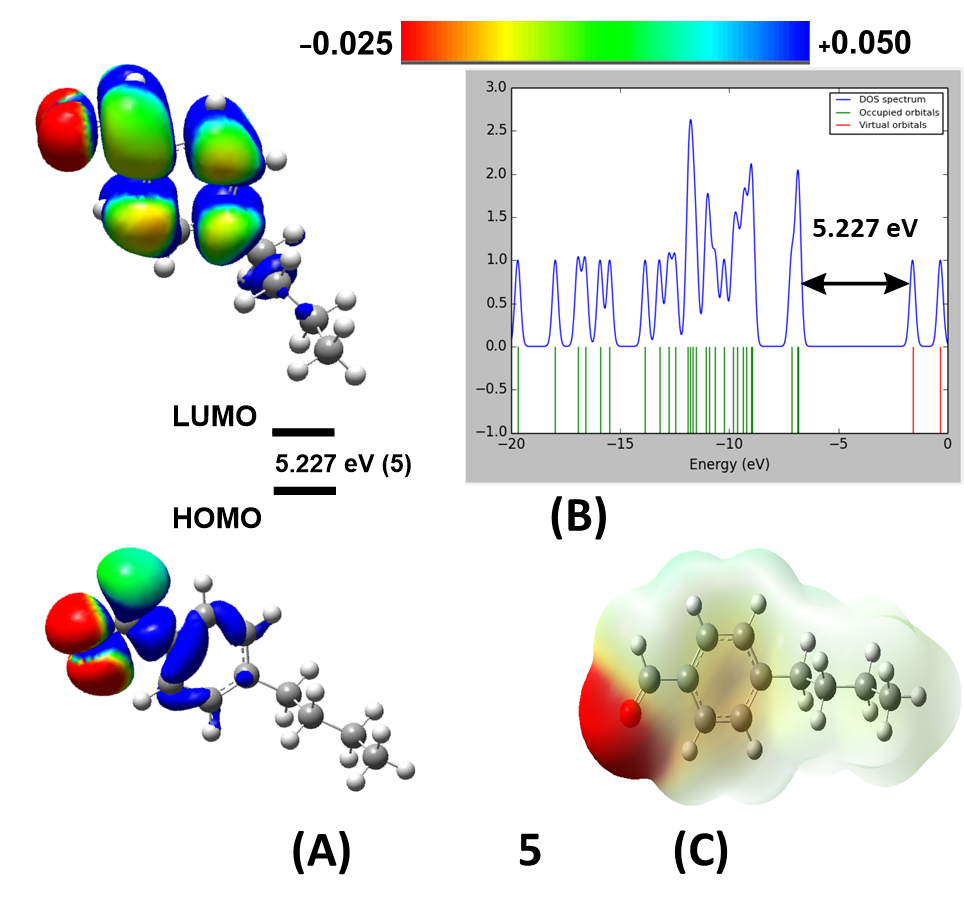
**

**Fig. S5.** (A) Molecular orbitals of isodensity surfaces (0.02 electrons Bohr−3 surface) (red = electron-rich, blue = electron-deficient) of HOMO and LUMO; (B) Maps of electrostatic potential (0.02 electrons Bohr−3 surface) (red = electron-rich, blue = electron-deficient); (C) DOS plot and HOMO-LUMO energy gap for the compound, **5.**

**
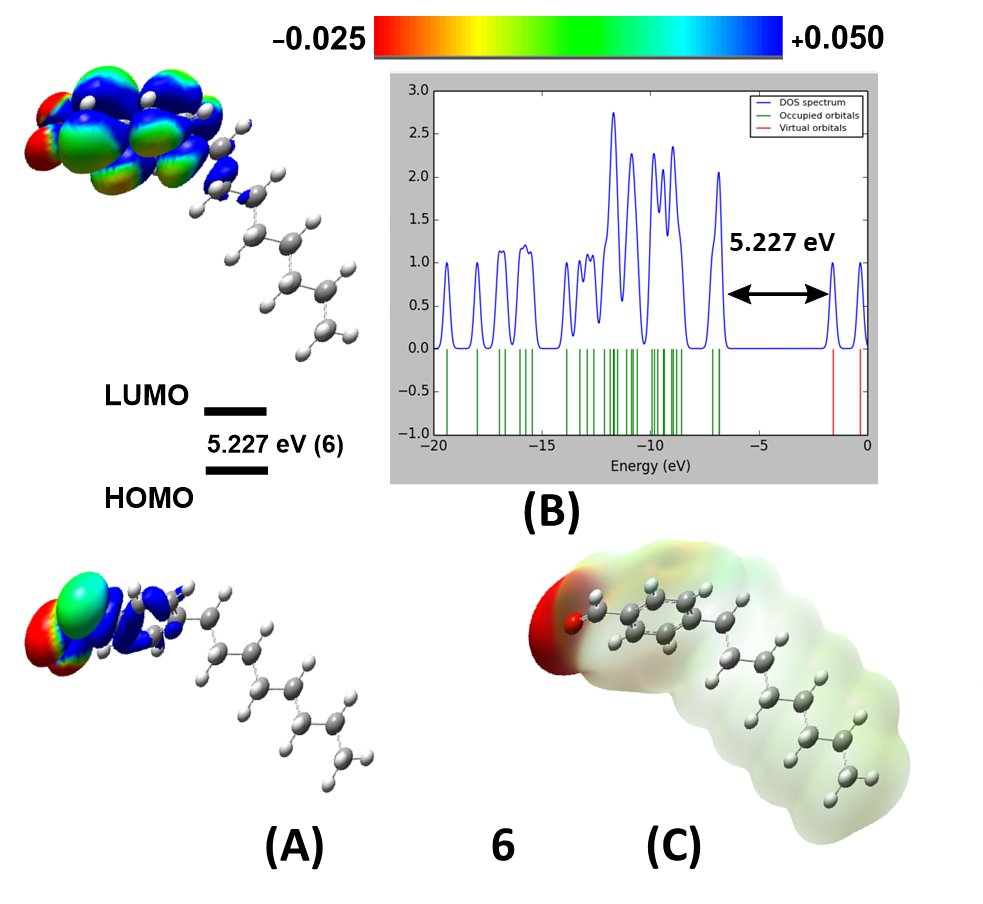
**

**Fig. S6.** (A) Molecular orbitals of isodensity surfaces (0.02 electrons Bohr−3 surface) (red = electron-rich, blue = electron-deficient) of HOMO and LUMO; (B) Maps of electrostatic potential (0.02 electrons Bohr−3 surface) (red = electron-rich, blue = electron-deficient); (C) DOS plot and HOMO-LUMO energy gap for the compound, **6.**

**
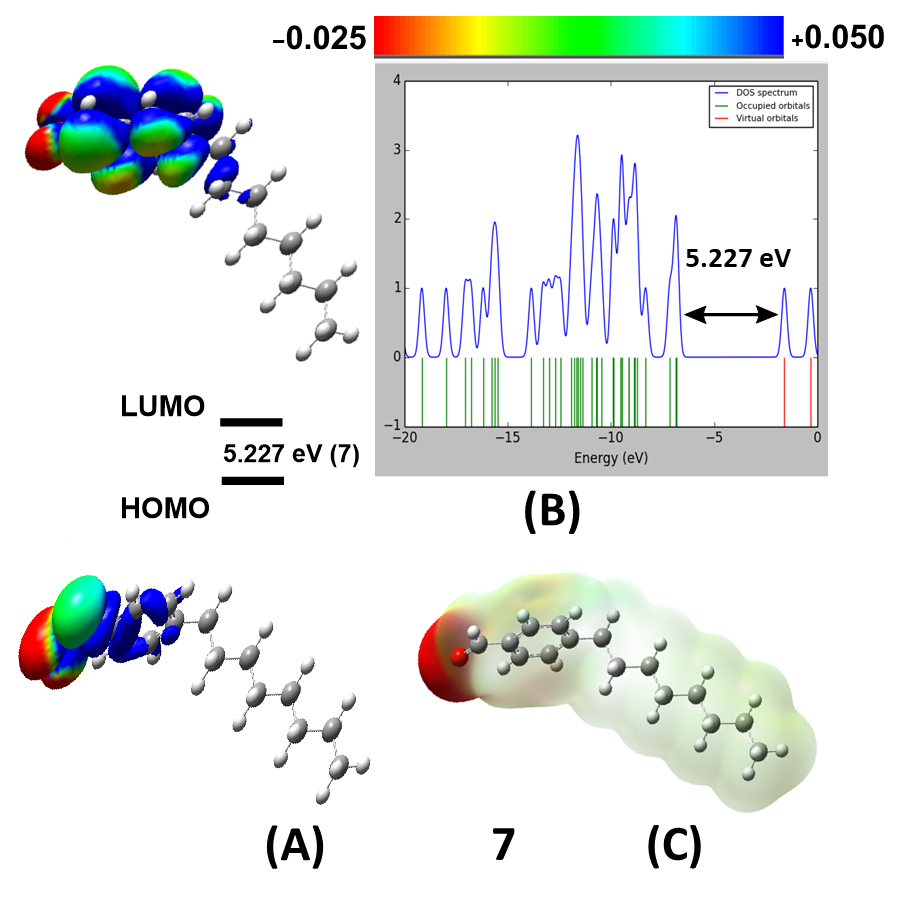
**

**Fig. S7.** (A) Molecular orbitals of isodensity surfaces (0.02 electrons Bohr−3 surface) (red = electron-rich, blue = electron-deficient) of HOMO and LUMO; (B) Maps of electrostatic potential (0.02 electrons Bohr−3 surface) (red = electron-rich, blue = electron-deficient); (C) DOS plot and HOMO-LUMO energy gap for the compound, **7.**

**
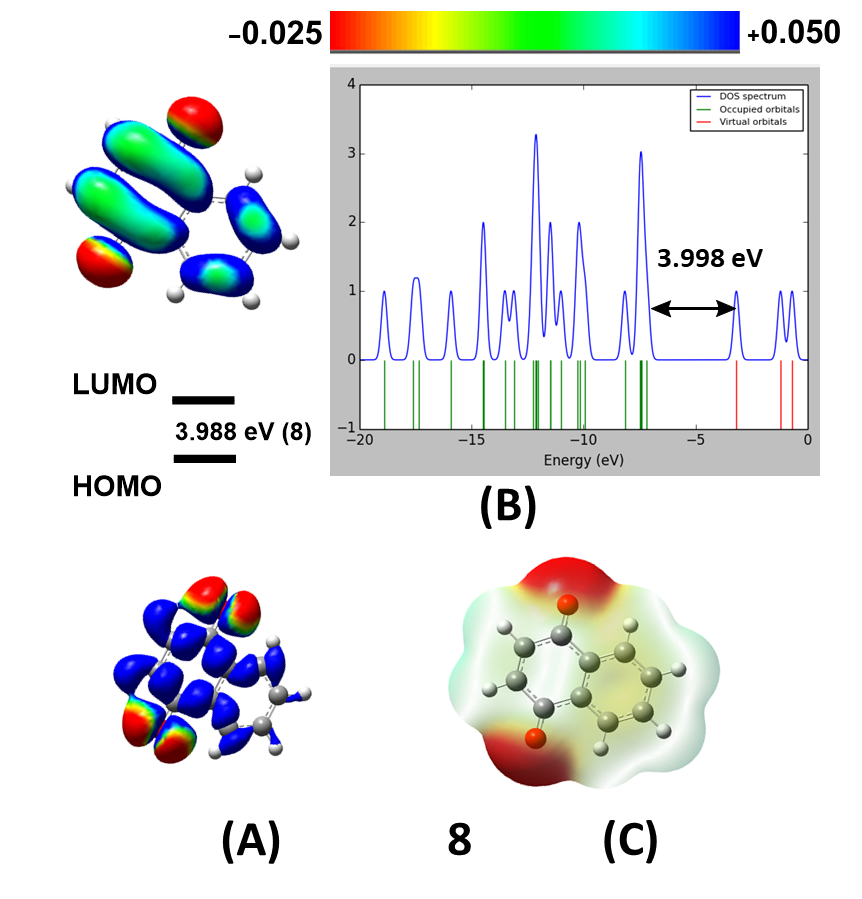
**

**Fig. S8.** (A) Molecular orbitals of isodensity surfaces (0.02 electrons Bohr−3 surface) (red = electron-rich, blue = electron-deficient) of HOMO and LUMO; (B) Maps of electrostatic potential (0.02 electrons Bohr−3 surface) (red = electron-rich, blue = electron-deficient); (C) DOS plot and HOMO-LUMO energy gap for the compound, **8.**

**
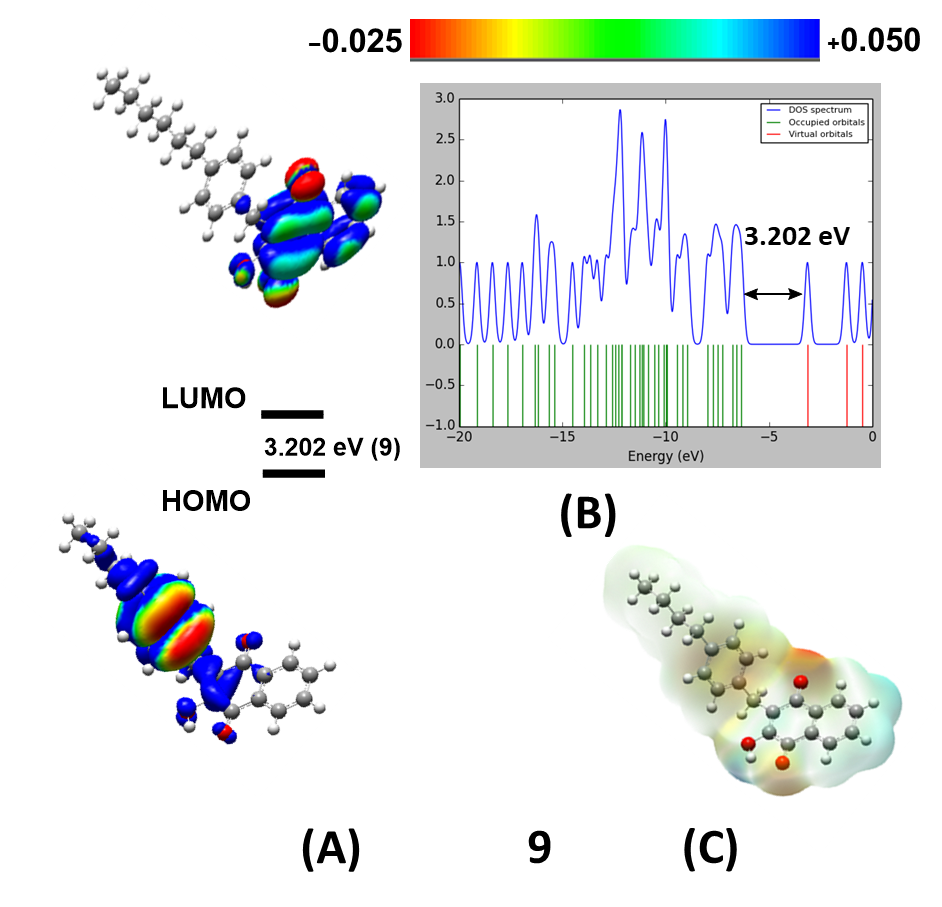
**

**Fig. S9.** (A) Molecular orbitals of isodensity surfaces (0.02 electrons Bohr−3 surface) (red = electron-rich, blue = electron-deficient) of HOMO and LUMO; (B) Maps of electrostatic potential (0.02 electrons Bohr−3 surface) (red = electron-rich, blue = electron-deficient); (C) DOS plot and HOMO-LUMO energy gap for the compound, **9.**

**
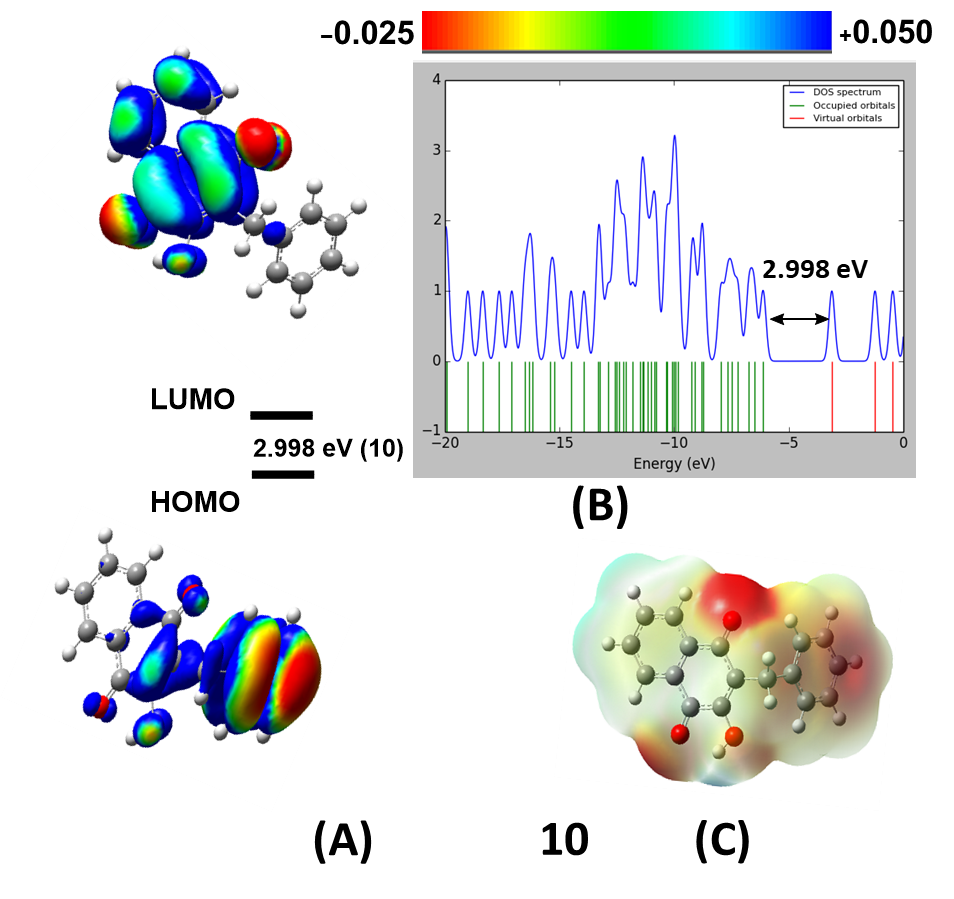
**

**Fig. S10.** (A) Molecular orbitals of isodensity surfaces (0.02 electrons Bohr−3 surface) (red = electron-rich, blue = electron-deficient) of HOMO and LUMO; (B) Maps of electrostatic potential (0.02 electrons Bohr−3 surface) (red = electron-rich, blue = electron-deficient); (C) DOS plot and HOMO-LUMO energy gap for the compound, **10.**

**
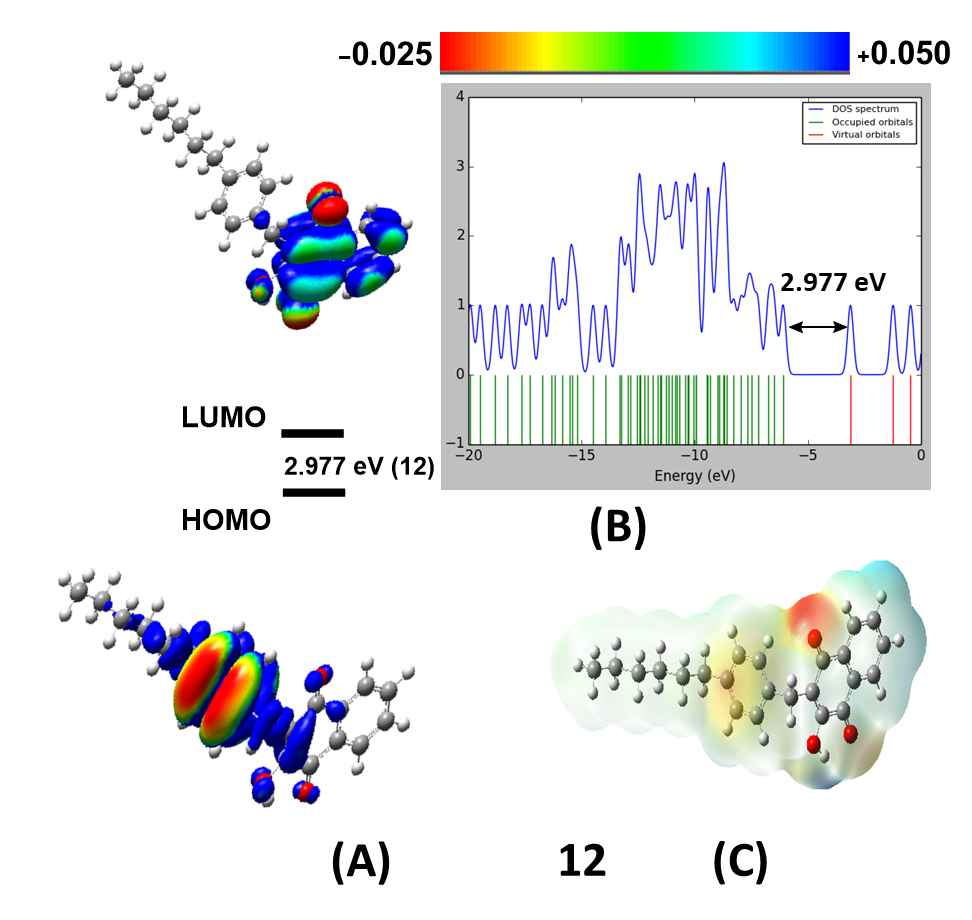
**

**Fig. S11.** (A) Molecular orbitals of isodensity surfaces (0.02 electrons Bohr−3 surface) (red = electron-rich, blue = electron-deficient) of HOMO and LUMO; (B) Maps of electrostatic potential (0.02 electrons Bohr−3 surface) (red = electron-rich, blue = electron-deficient); (C) DOS plot and HOMO-LUMO energy gap for the compound, **12.**

**
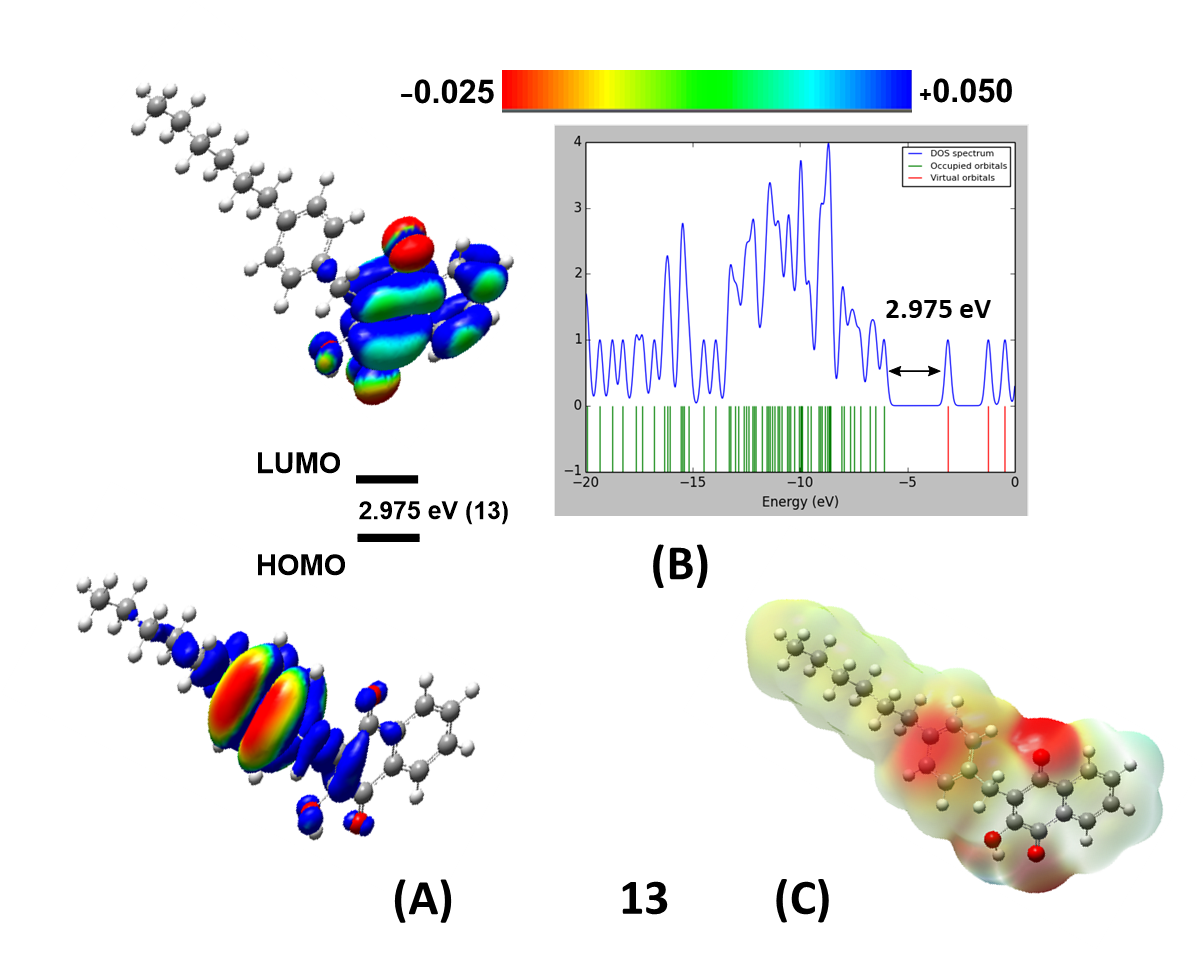
**

**Fig. S12.** (A) Molecular orbitals of isodensity surfaces (0.02 electrons Bohr−3 surface) (red = electron-rich, blue = electron-deficient) of HOMO and LUMO; (B) Maps of electrostatic potential (0.02 electrons Bohr−3 surface) (red = electron-rich, blue = electron-deficient); (C) DOS plot and HOMO-LUMO energy gap for the compound, **13.**

**
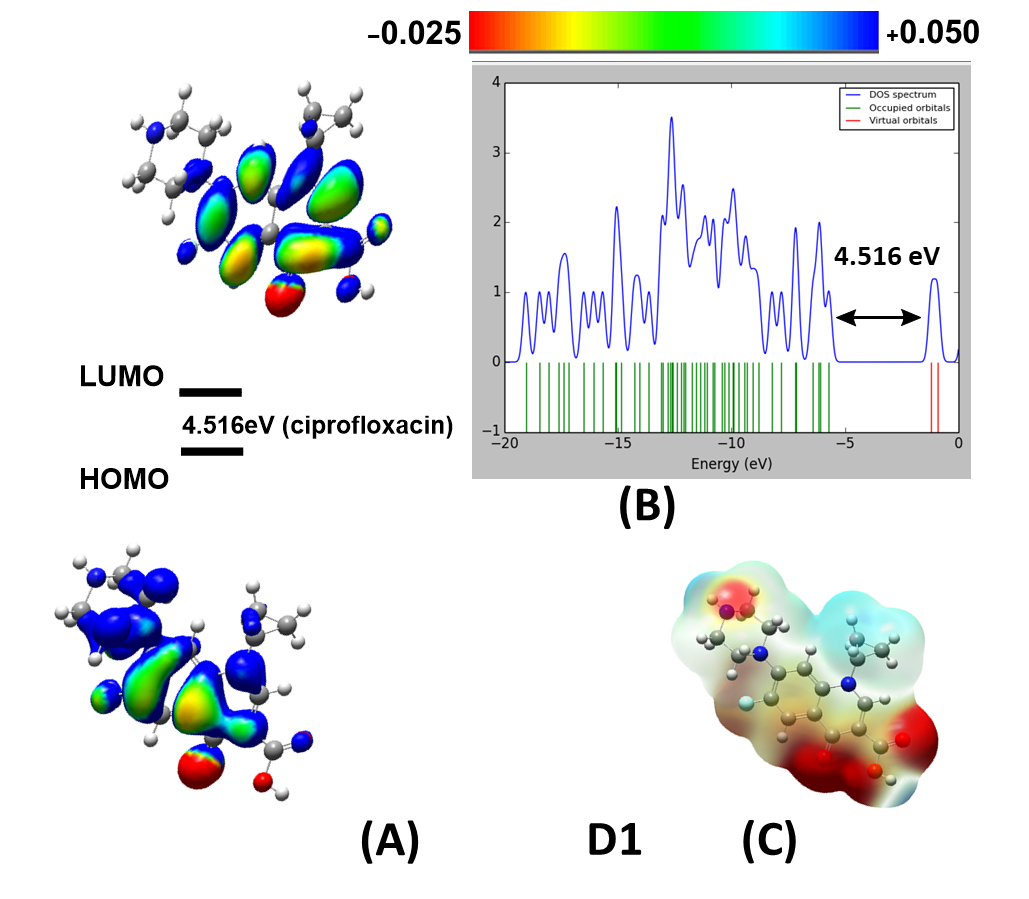
**

**Fig. S13.** (A) Molecular orbitals of isodensity surfaces (0.02 electrons Bohr−3 surface) (red = electron-rich, blue = electron-deficient) of HOMO and LUMO; (B) Maps of electrostatic potential (0.02 electrons Bohr−3 surface) (red = electron-rich, blue = electron-deficient); (C) DOS plot and HOMO-LUMO energy gap for the compound, **ciprofloxacin (D1).**

**
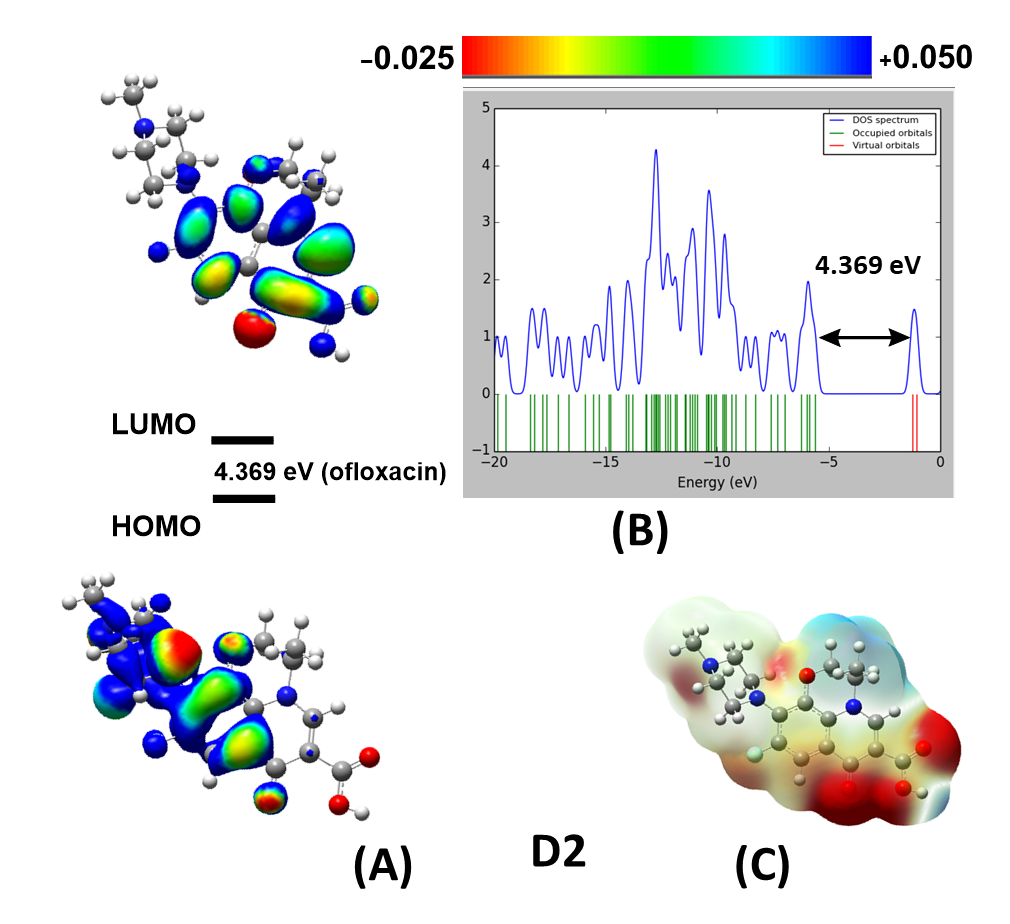
**

**Fig. S14.** (A) Molecular orbitals of isodensity surfaces (0.02 electrons Bohr−3 surface) (red = electron-rich, blue = electron-deficient) of HOMO and LUMO; (B) Maps of electrostatic potential (0.02 electrons Bohr−3 surface) (red = electron-rich, blue = electron-deficient); (C) DOS plot and HOMO-LUMO energy gap for the compound, **ofloxacin (D2).**

**
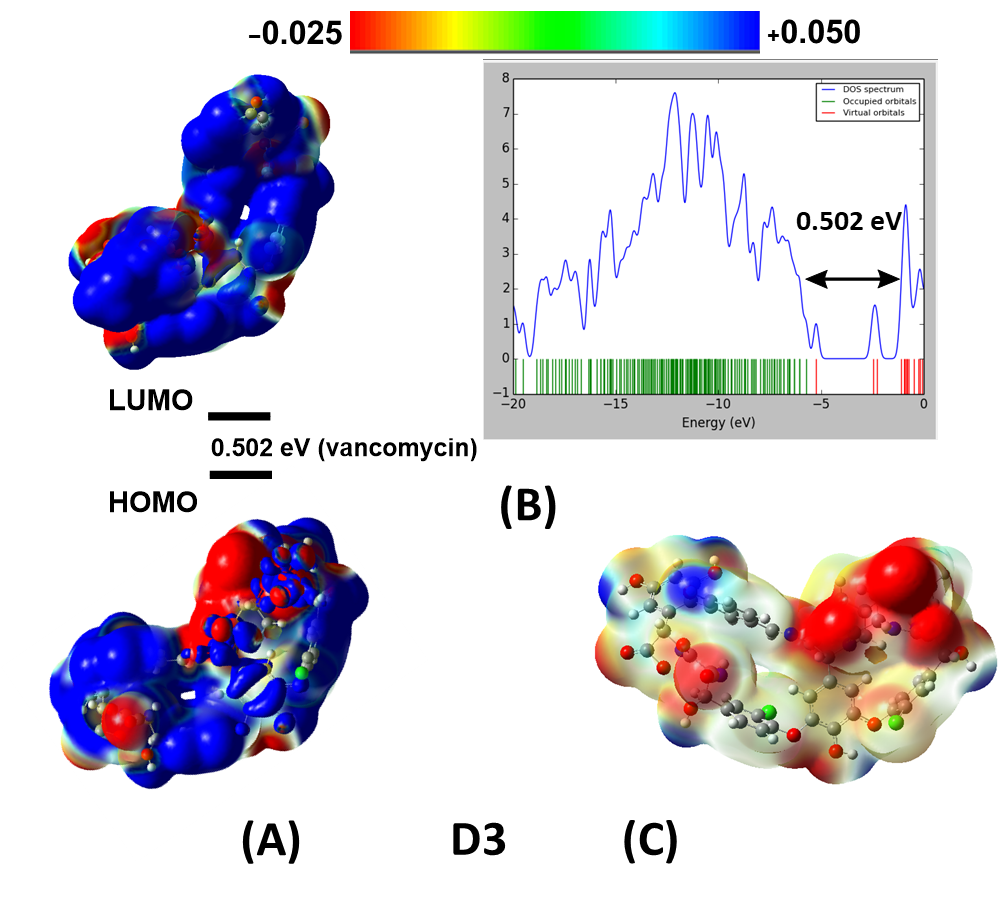
**

**Fig. S15.** (A) Molecular orbitals of isodensity surfaces (0.02 electrons Bohr−3 surface) (red = electron-rich, blue = electron-deficient) of HOMO and LUMO; (B) Maps of electrostatic potential (0.02 electrons Bohr−3 surface) (red = electron-rich, blue = electron-deficient); (C) DOS plot and HOMO-LUMO energy gap for the compound, **vancomycin (D3).**

**
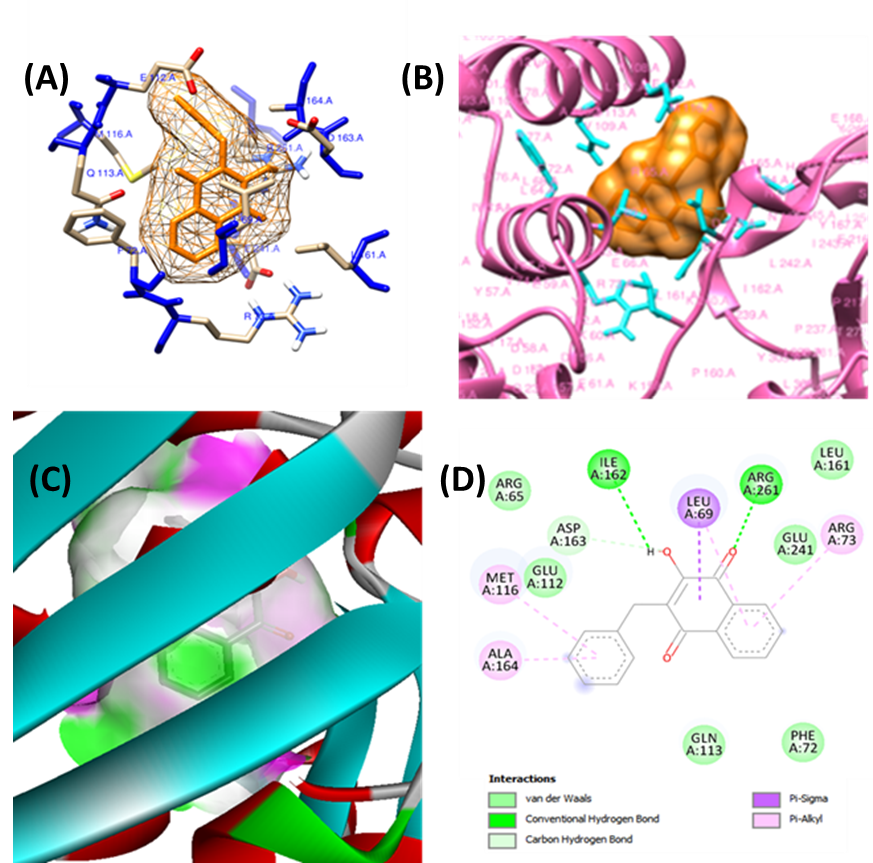
**

**Fig. S16.** Molecular docking results: (A) ligand binding in protein pocket; (B) hydrogen bonding; (C) hydrogen bonding in solid state; (D) ligand-protein interaction in 2D diagram, for compound **9** in 3E0M.


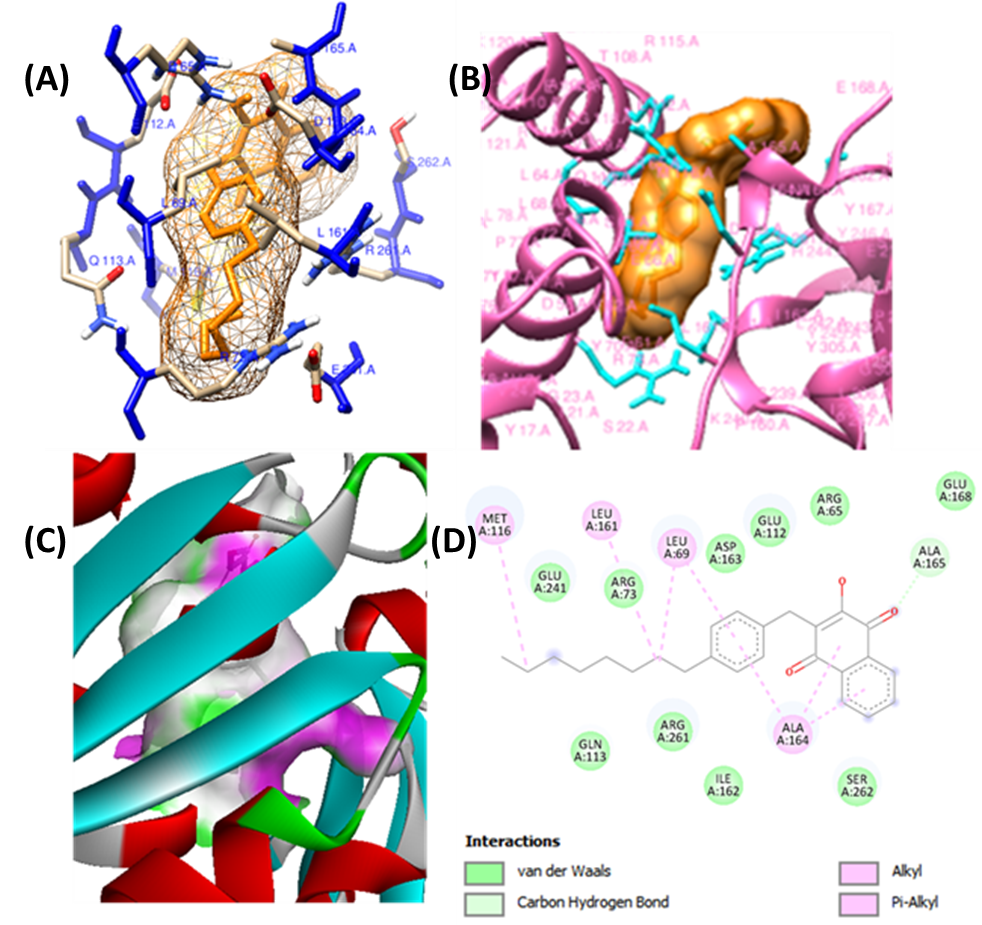


**Fig. S17.** Molecular docking results: (A) ligand binding in protein pocket; (B) hydrogen bonding; (C) hydrogen bonding in solid state; (D) ligand-protein interaction in 2D diagram, for compound **13** in 3E0M.


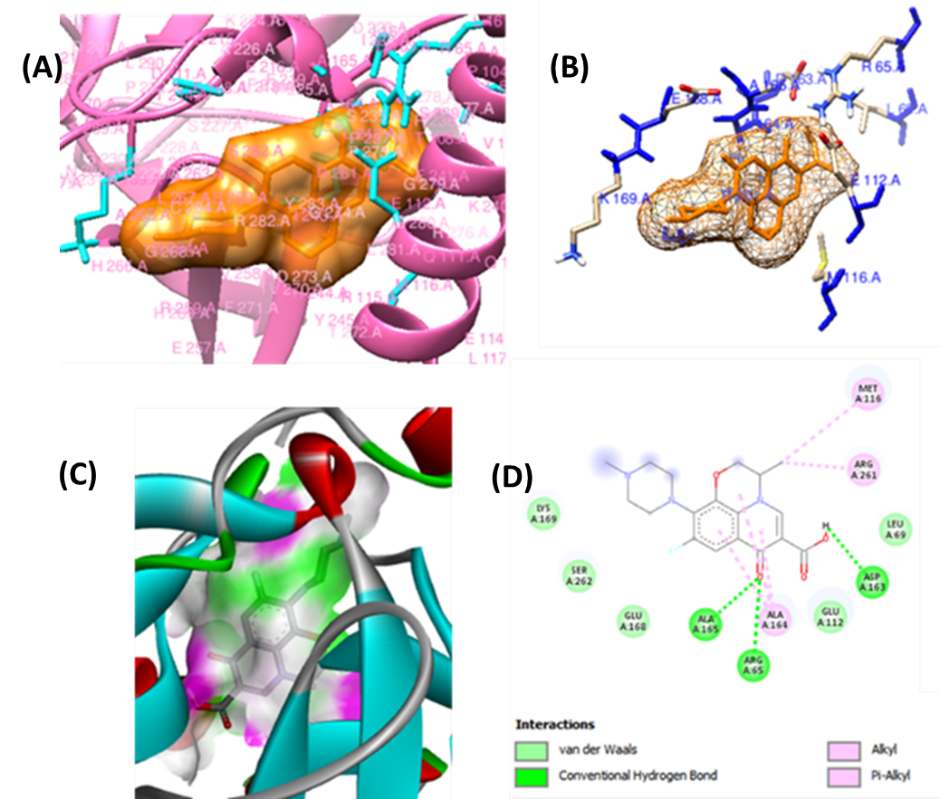


**Fig. S18.** Molecular docking results: (A) ligand binding in protein pocket; (B) hydrogen bonding; (C) hydrogen bonding in solid state; (D) ligand-protein interaction in 2D diagram, for compound **ofloxacin** in 3E0M.


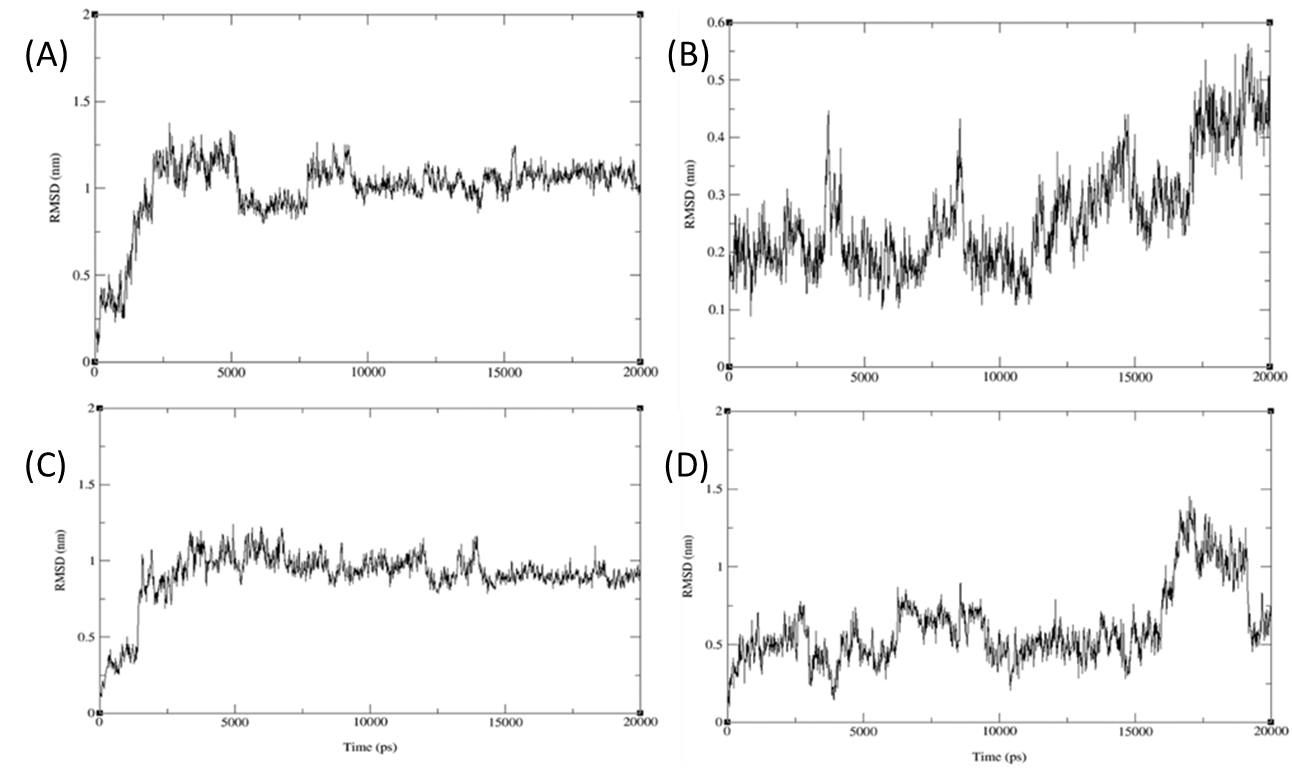


**Fig. S19.** RMSD evaluation of ligand-protein complexes with 3E0M for compounds **9**, **11**, **13**, and Ofloxacin at 300 K during 20 ns MD simulations.

**
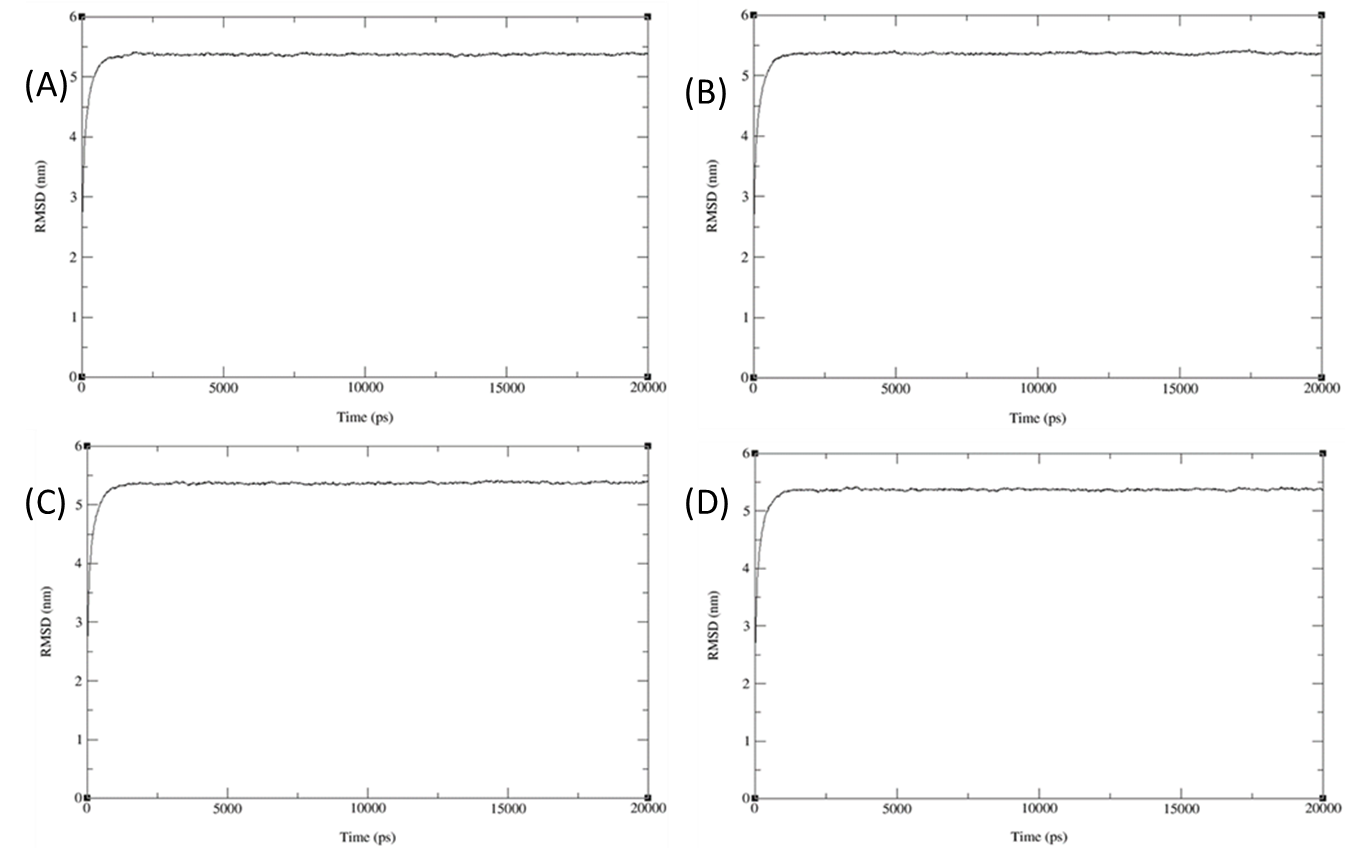
**

**Fig. S20.** RMSD evaluation of protein 3E0M in complex with compounds 9, 11, 13, and Ofloxacin at 300 K during 20 ns MD simulations.

**
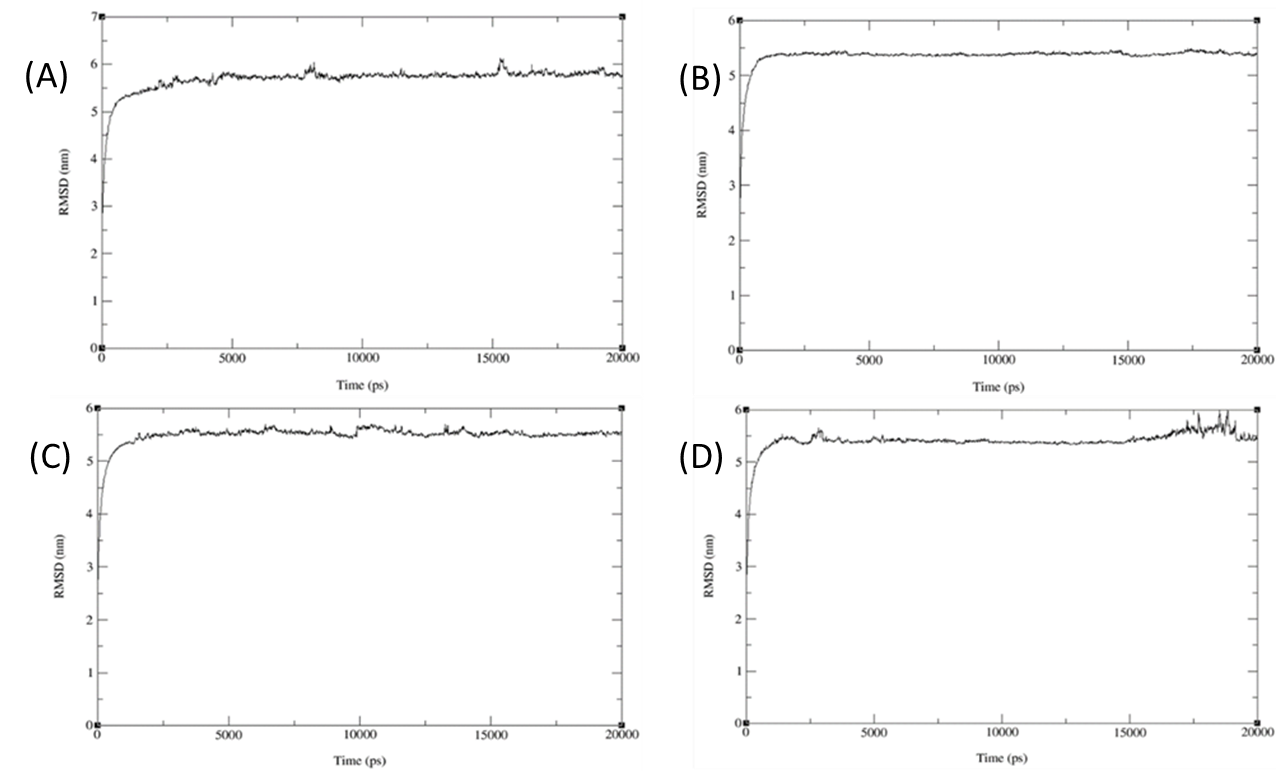
**

**Fig. S21.** RMSD evaluation of complexes (A) compound **9** with 3E0M , (B) compound **11** with 3E0M, (C) compound **13** with 3E0M, (D) Olioxacin with 3E0M; at 300K, during the 20 ns MD simulation.

**
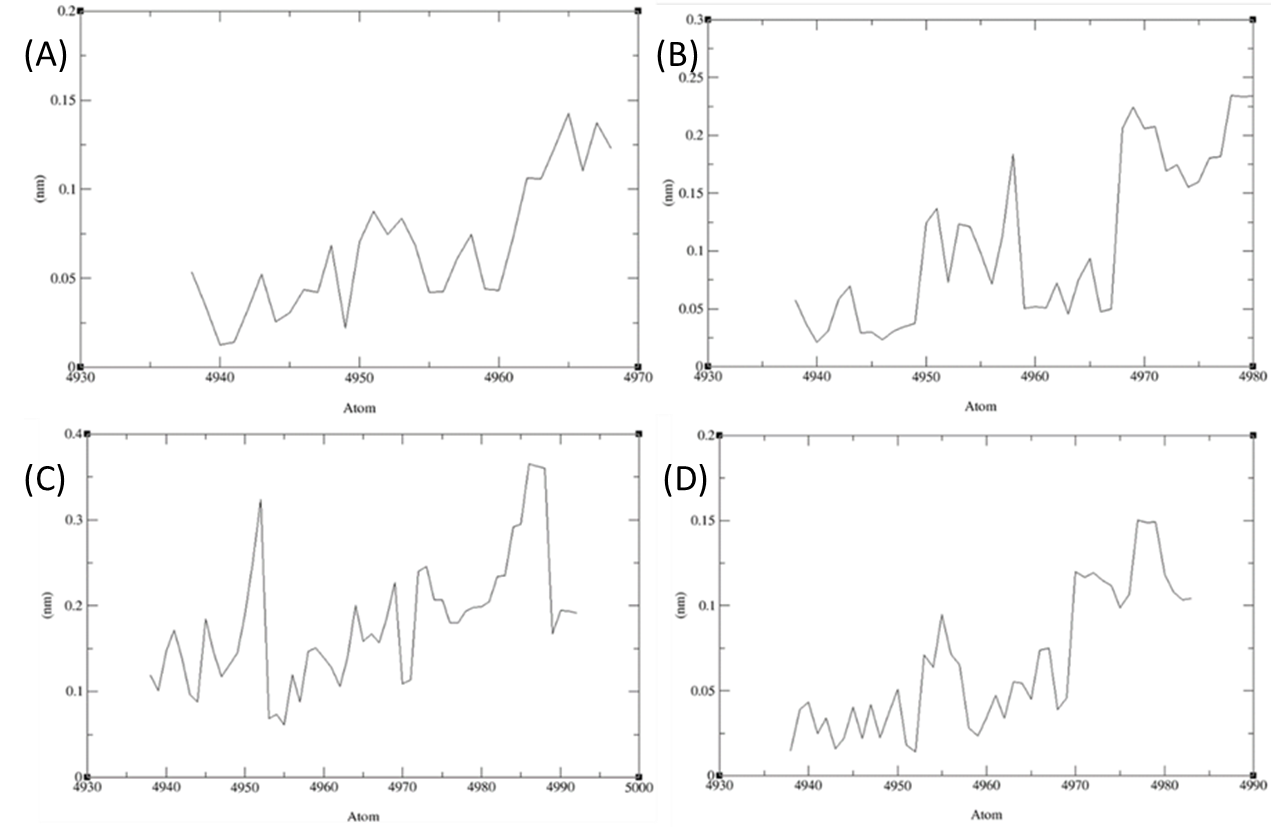
**

**Fig. S22.** RMSF evaluation of ligand-protein complexes for compounds **9**, **11**, **13**, and ofloxacin at 300 K during 20 ns MD simulations.


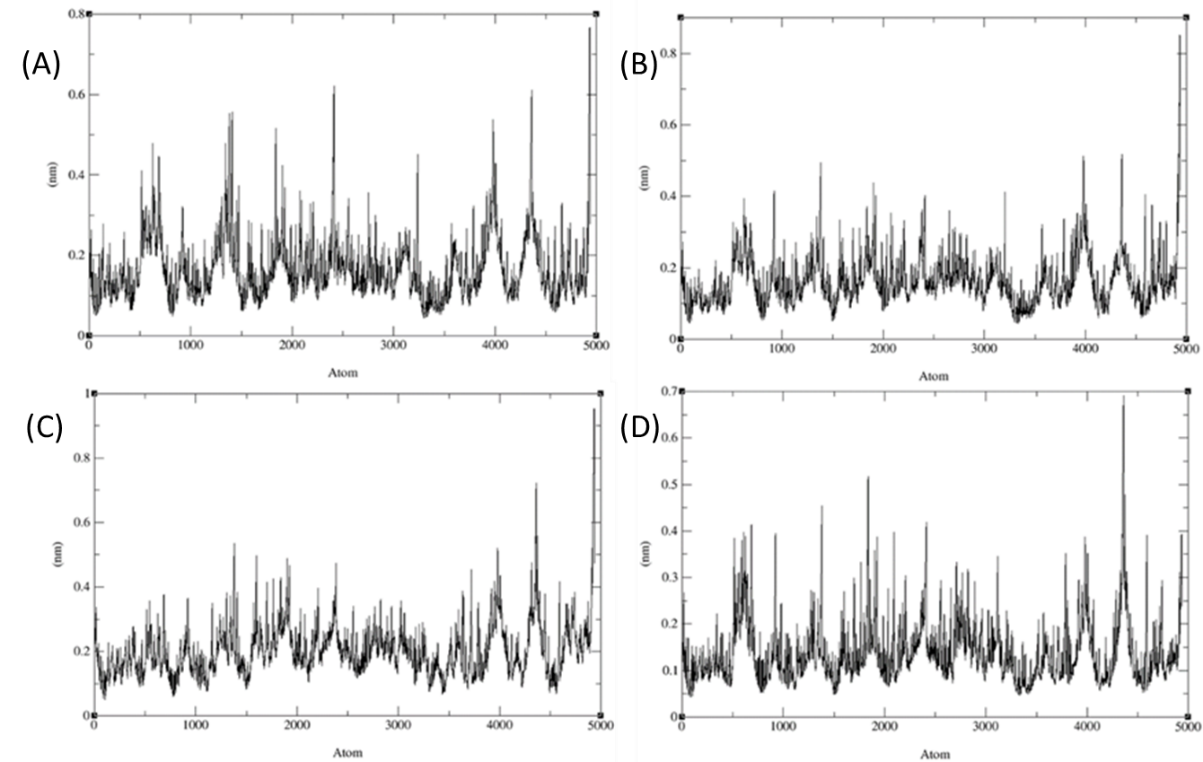


**Fig. S23.** RMSF evaluation of protein 3E0M in complex with compounds **9**, **11**, **13**, and Olioxacin at 300 K during 20 ns MD simulations

**
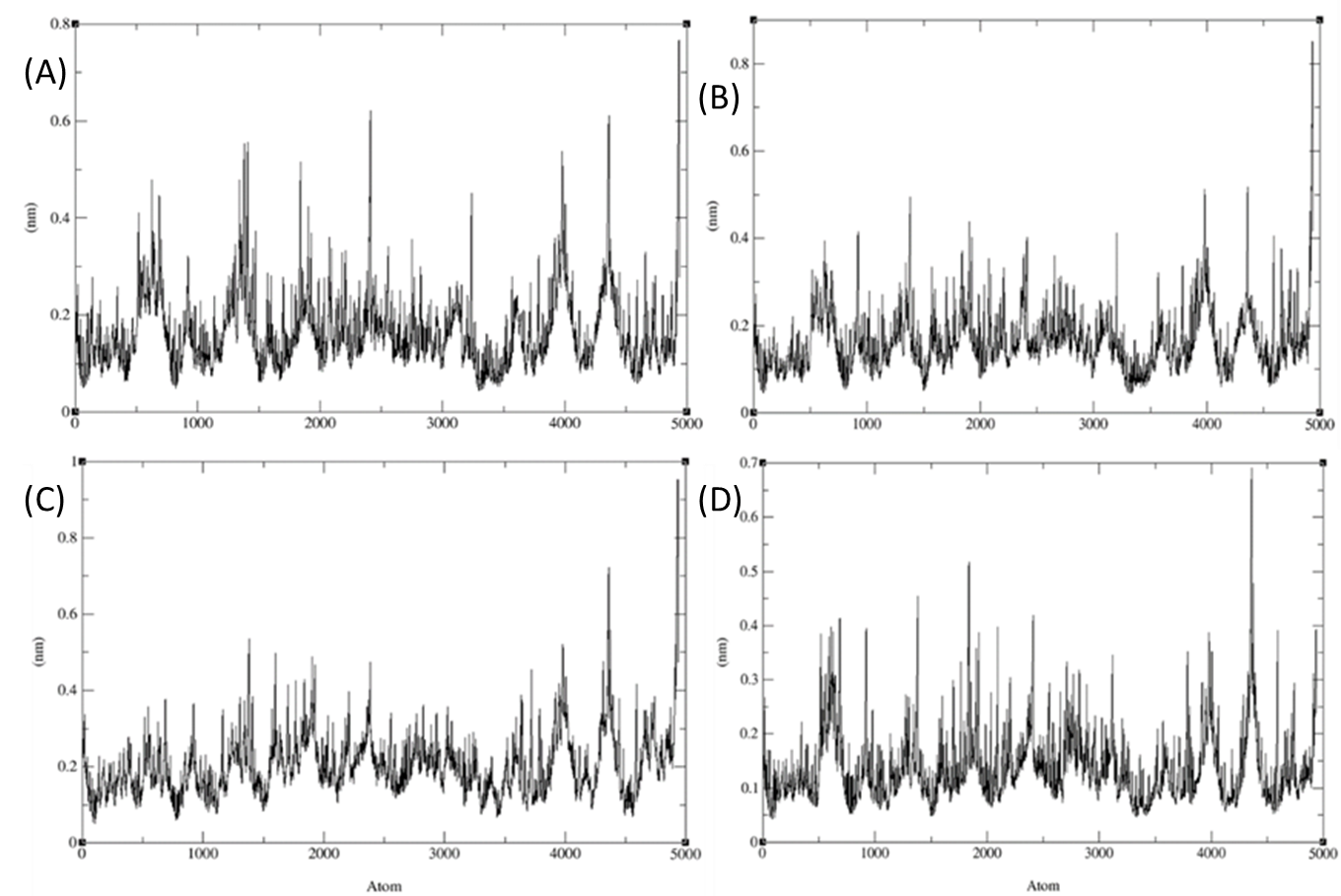
**

**Fig. S24.** RMSF evaluation of complexes (A) compound **9** with 3E0M, (B) compound **11** with 3E0M, (C) compound **13** with 3E0M, (D) Ofloxacin with 3E0M; at 300K, during the 20 ns MD simulation.


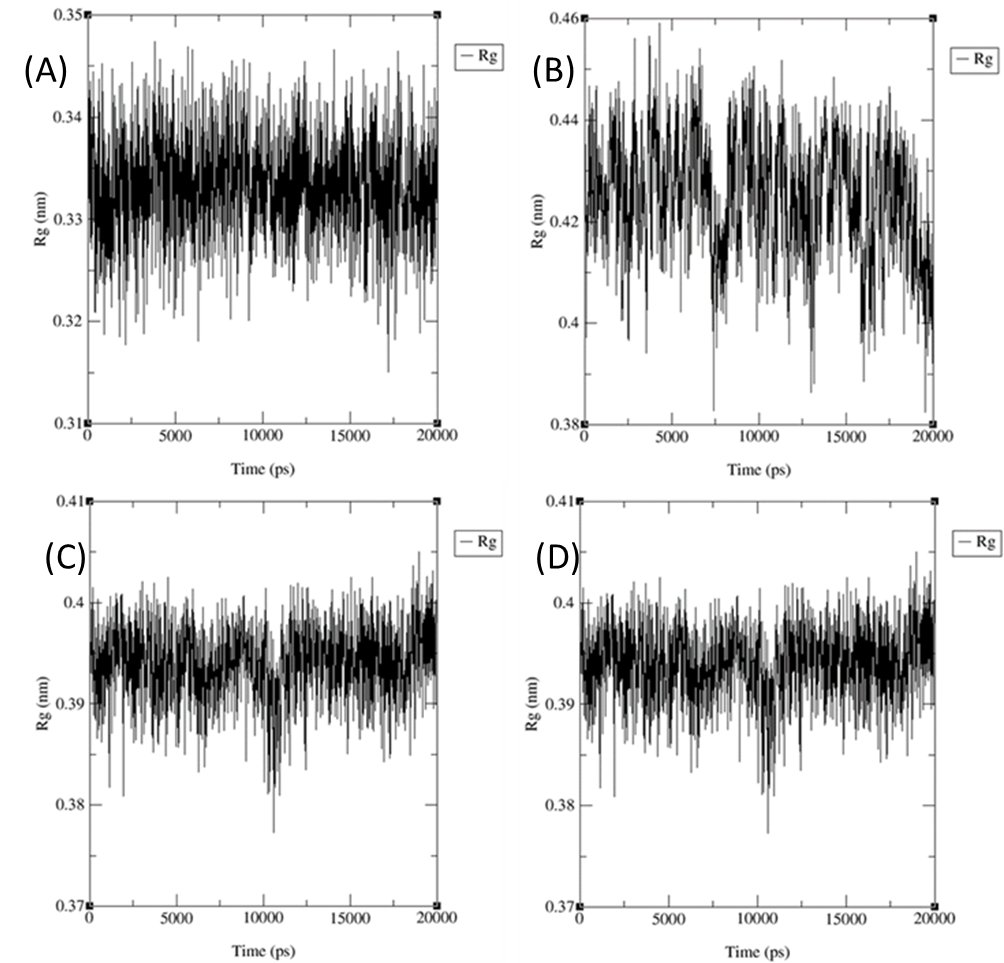


**Fig. S25.** Rg evaluation of ligand-protein complexes for compounds **9**, **11**, **13**, and Olioxacin at 300 K during 20 ns MD simulations.


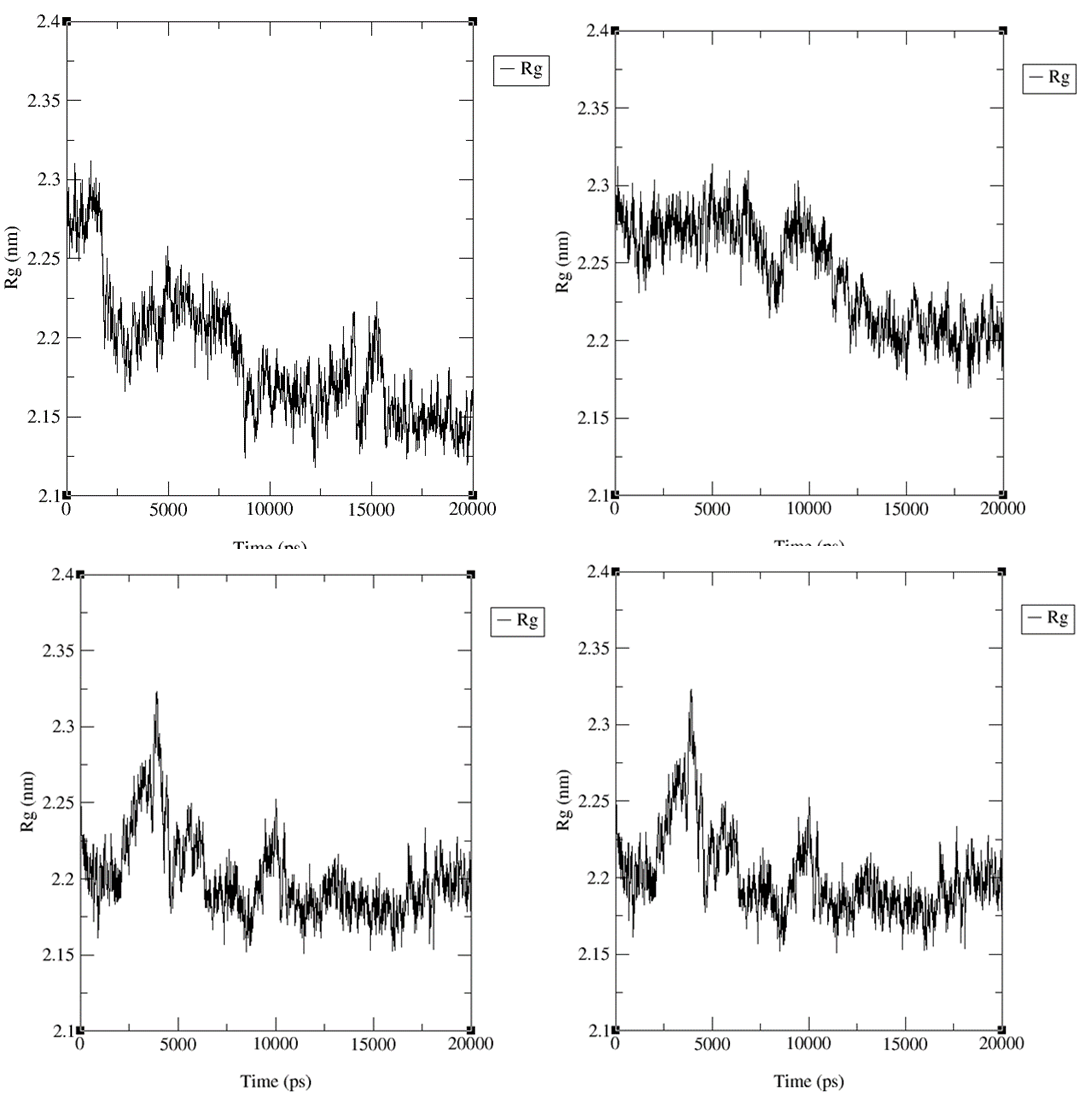


**Fig. S26.** Rg evaluation of protein 3E0M in complex with compounds **9**, **11**, **13**, and Ofloxacin at 300 K during 20 ns MD simulations


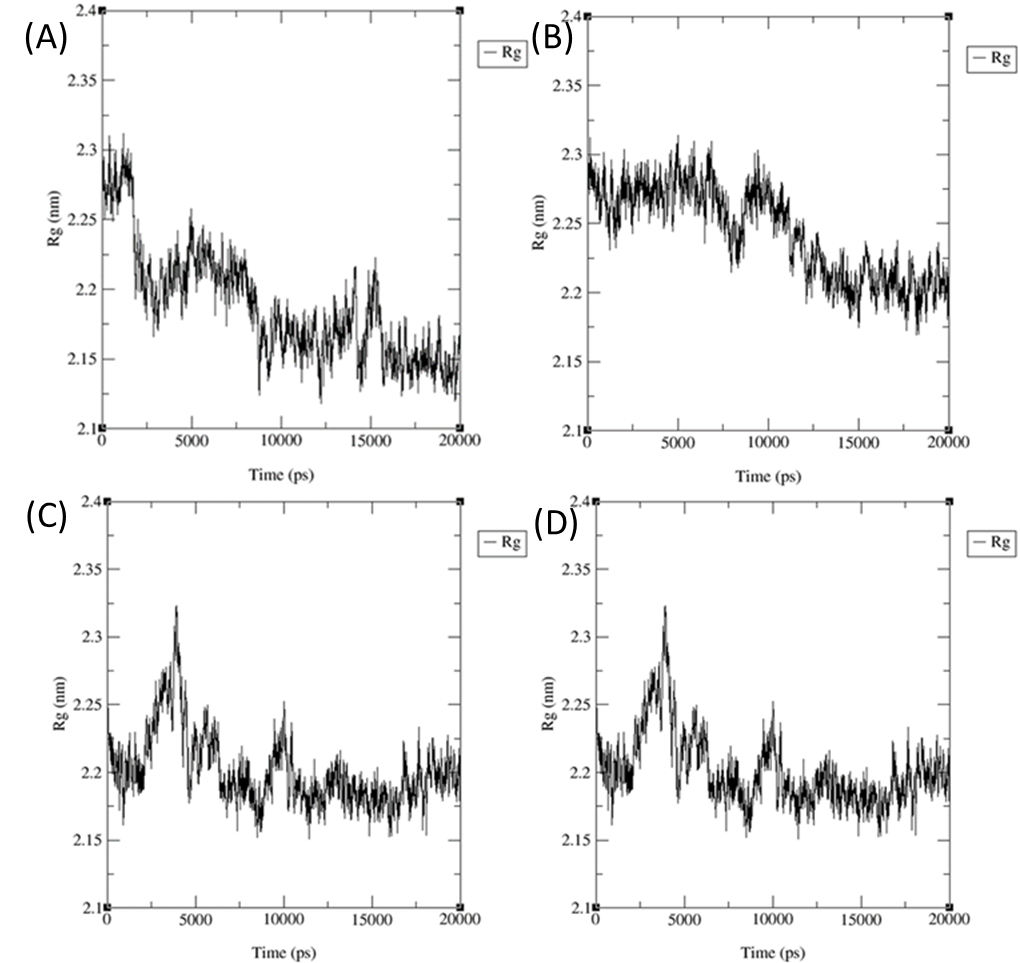


**Fig. S27.** Rg evaluation of complexes (A) compound **9** with 3E0M, (B) compound **11** with 3E0M, (C) compound **13** with 3E0M, (D) Ofloxacin with 3E0M; at 300K, during the 20 ns MD simulation.

**
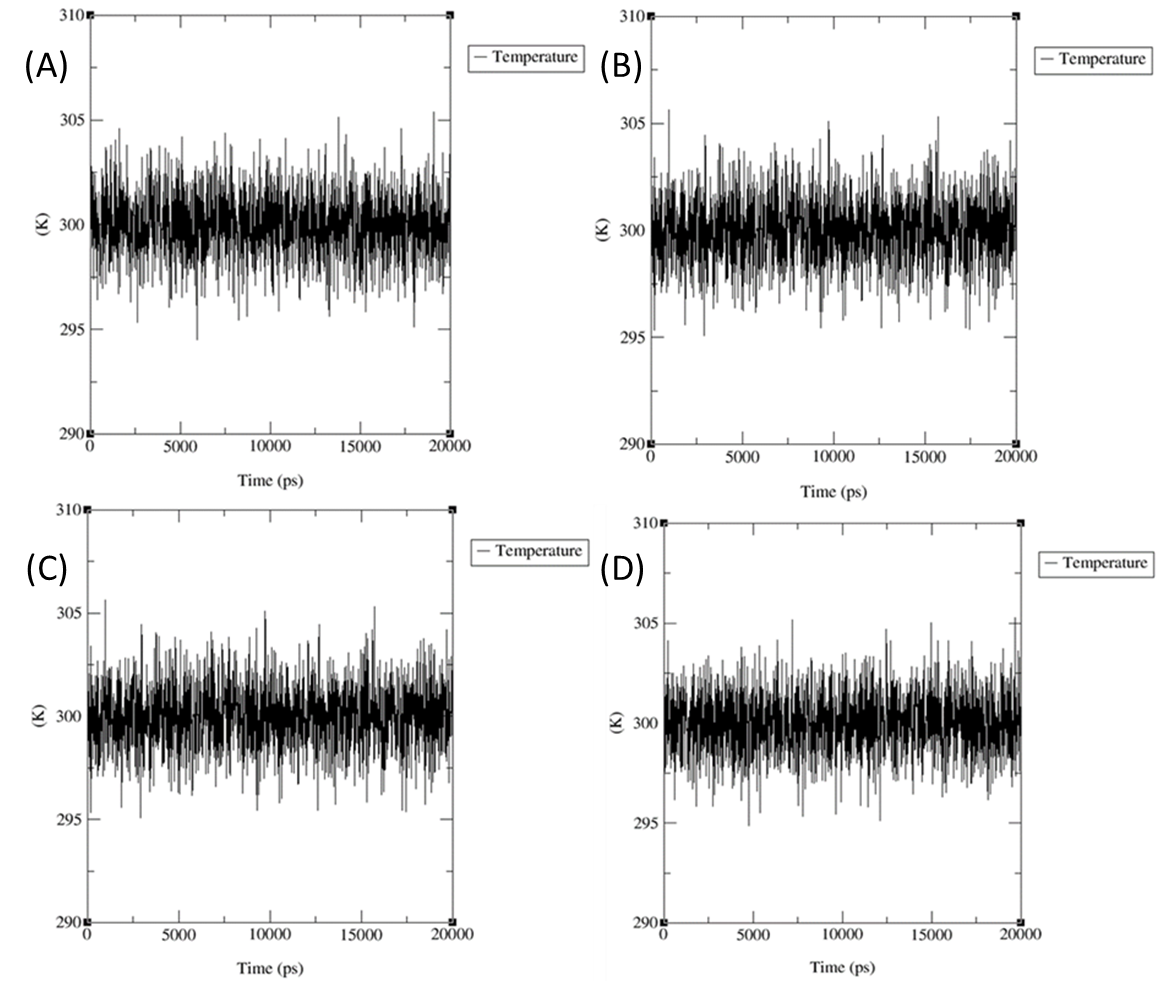
**

**Fig. S28.** Temperature 300K evaluation of complexes (A) compound **9** with 3E0M, (B) compound **11** with 3E0M, (C) compound **13** with 3E0M, (D) Ofloxacin with 3E0M; at 300K, during the 20 ns MD simulation.


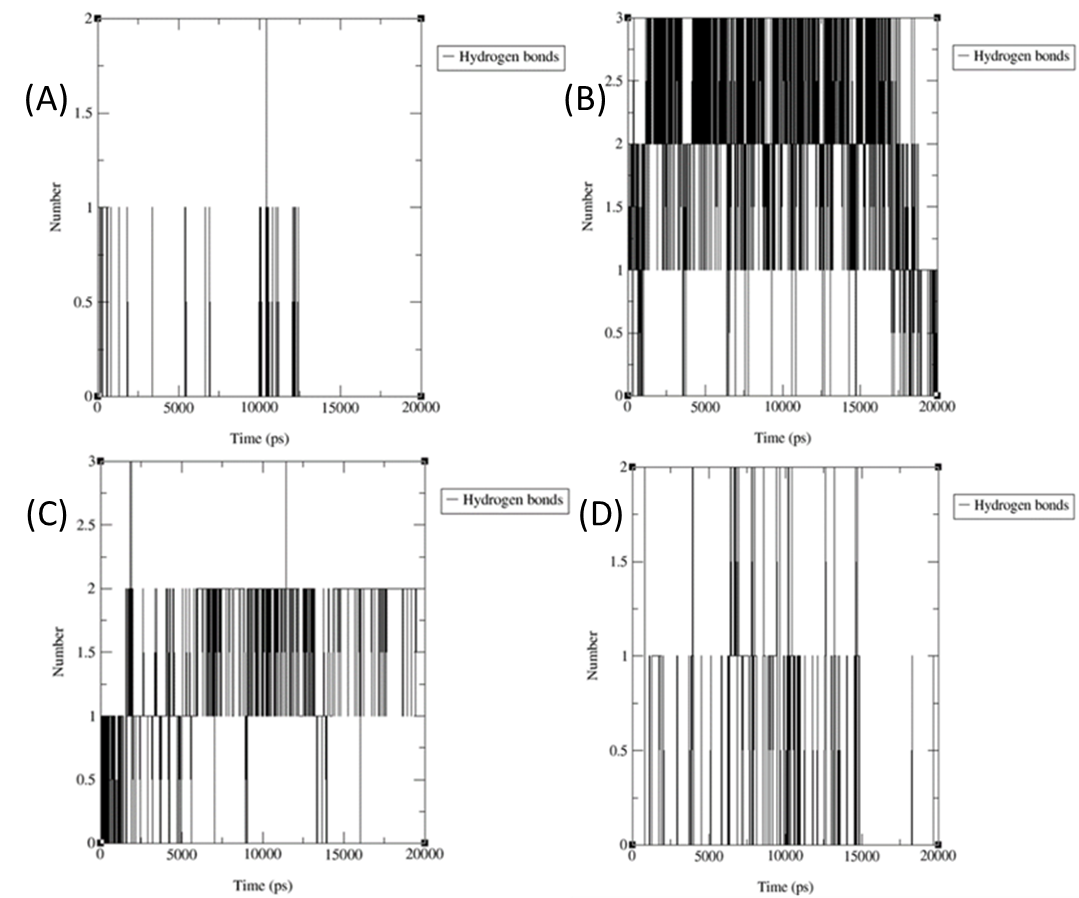


**Fig. S29.** Hydrogen bond evaluation of complexes (A) compound **9** with 3E0M, (B) compound **11** with 3E0M, (C) compound **13** with 3E0M, (D) Ofloxacin with 3E0M; at 300K, during the 20 ns MD simulation.


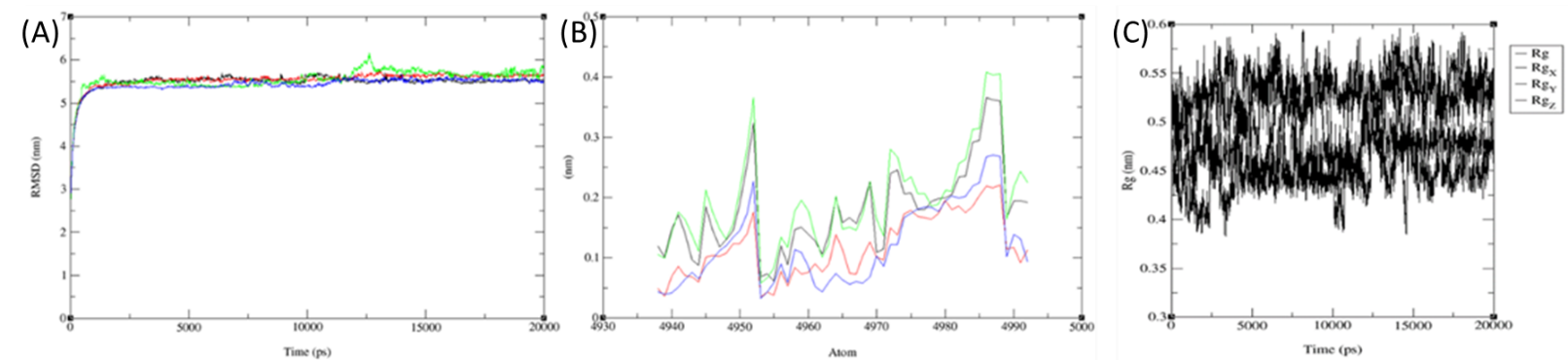


**Fig. S30**. Ligand 11 (A) Marge RMSD, (B) Marge RMSF, (C) Marge Gyration; at 300K, 305K, 310K, and 320K.


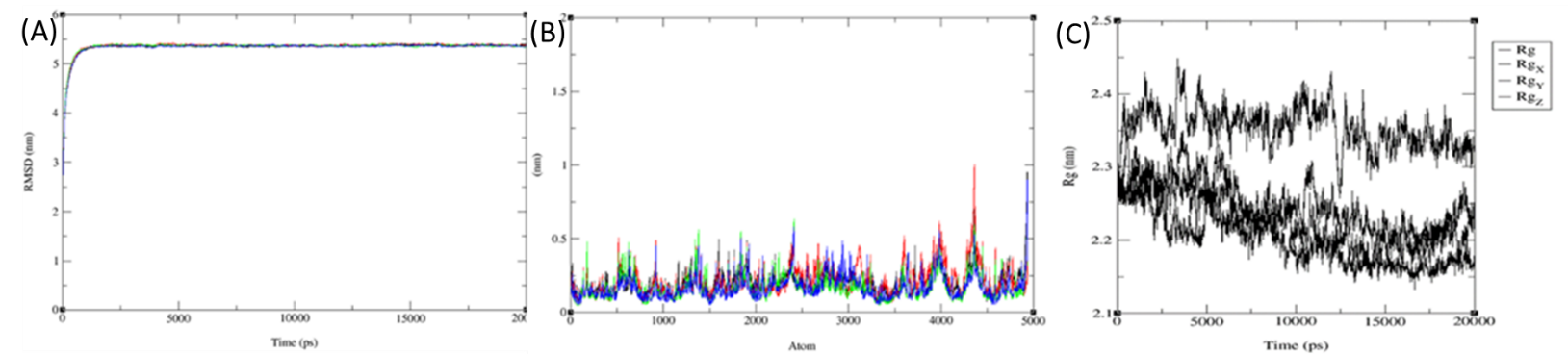


**Fig. S31.** Protein 3E0M (A) Marge RMSD, (B) Marge RMSF, (C) Marge Gyration; at 300K, 305K, 310K, and 320K.


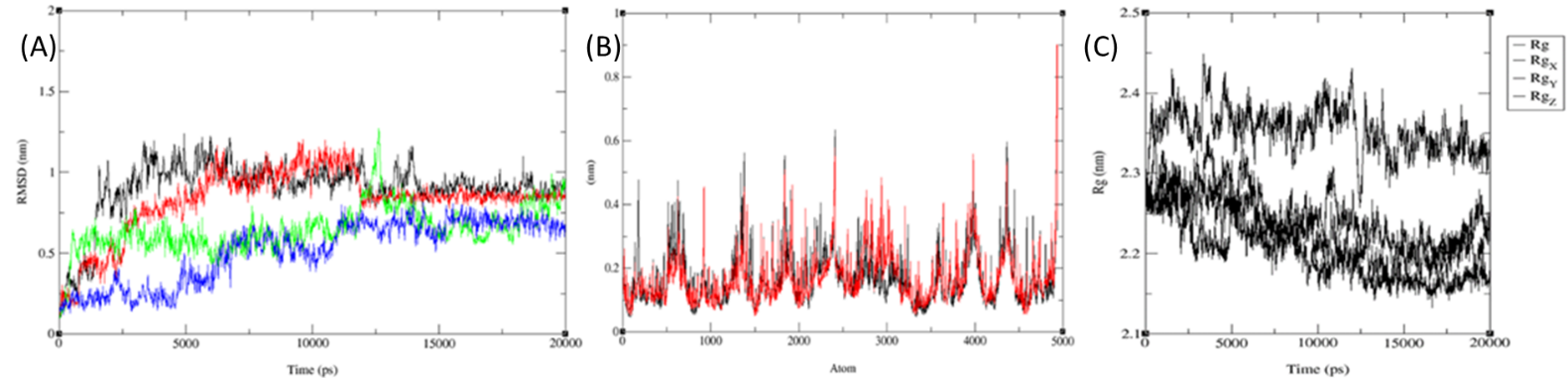


**Fig. S32.** The compound 11 and protein 3E0M complex (A) Marge RMSD, (B) Marge RMSF, (C) Marge Gyration; at 300K, 305K, 310K, and 320K.


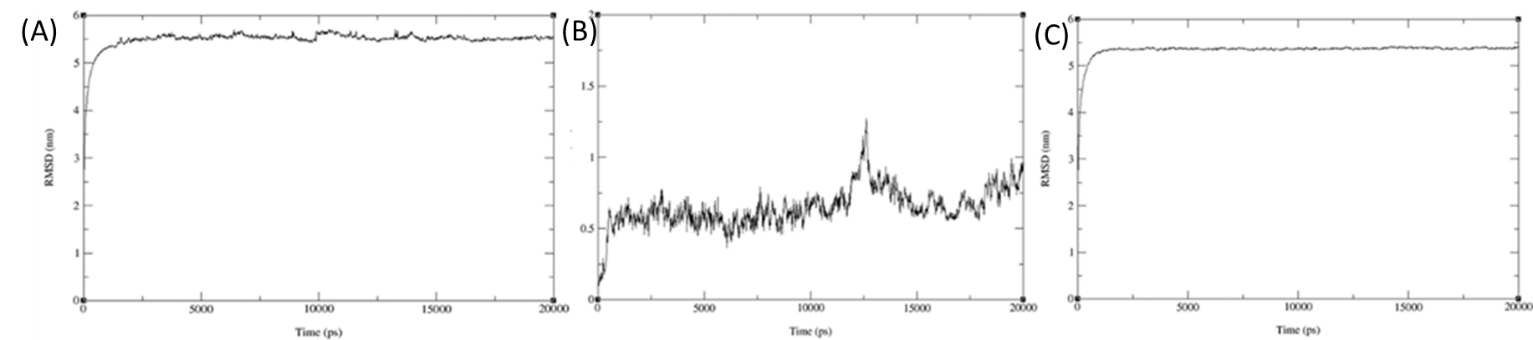


**Fig. S33**. The RMSD evaluation of compound 11 and protein 3E0M complex (A) Ligand, (B) Protein AND Ligand complex, (C) Protein; at 300K, , during the 20 ns MD simulation.


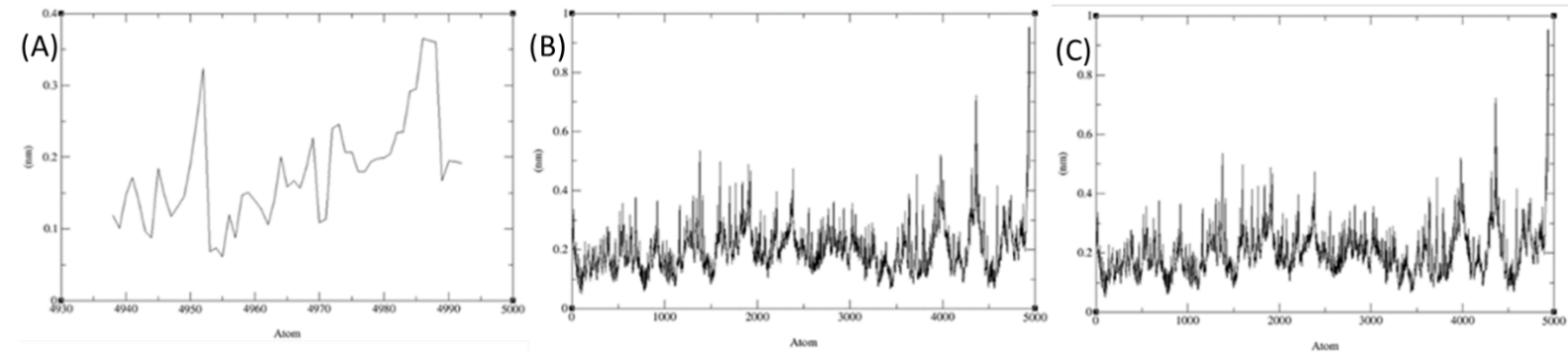


**Fig S34.** The RMSF evaluation of compound 11 and protein 3E0M complex (A) Ligand, (B) Protein, (C) Protein and Ligand complex; at 300K, , during the 5 ns MD simulation.


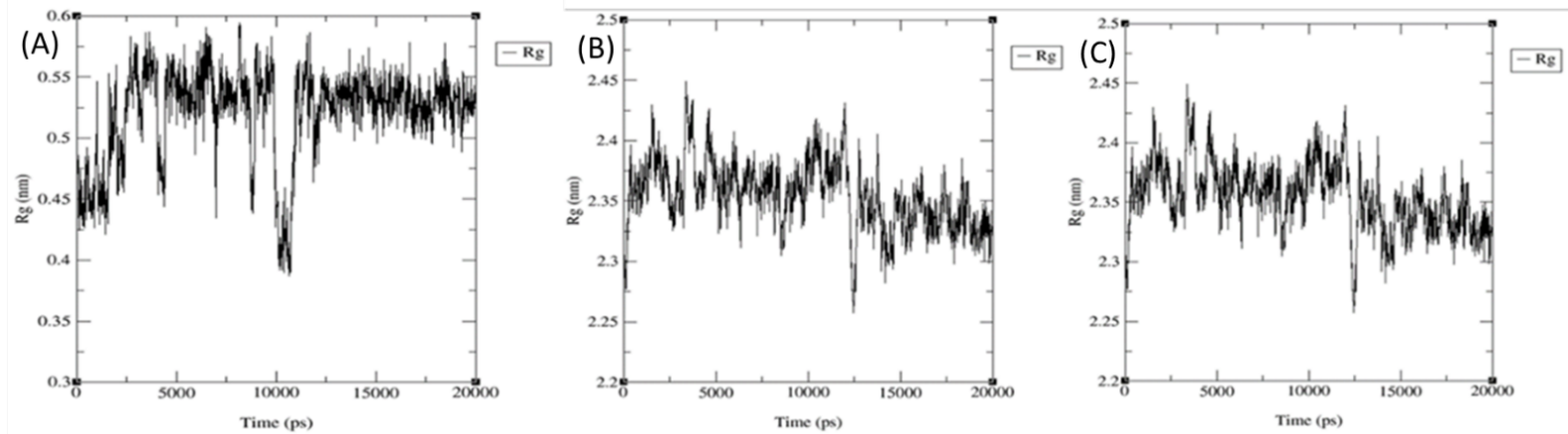


**Fig. S35**. The Gyration evaluation of compound 11 and protein 3E0M complex (A) Ligand, (B) Protein, (C) Protein and Ligand complex; at 300K, , during the 20 ns MD simulation.


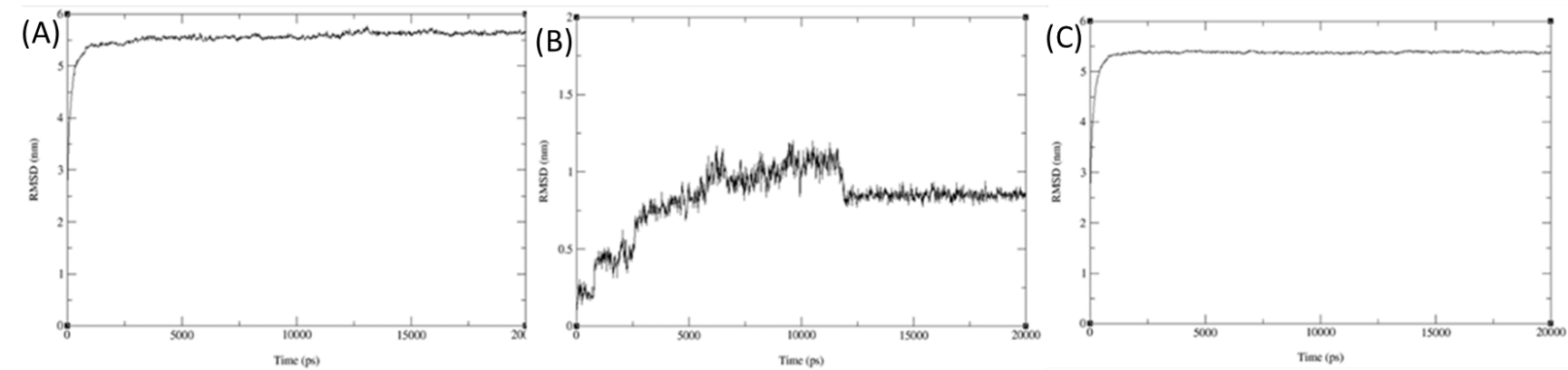


**Fig. S36.** The RMSD evaluation of compound 11 and protein 3E0M complex (A) Ligand, (B) Protein, (C) Protein and Ligand complex; at 305K, , during the 20 ns MD simulation.


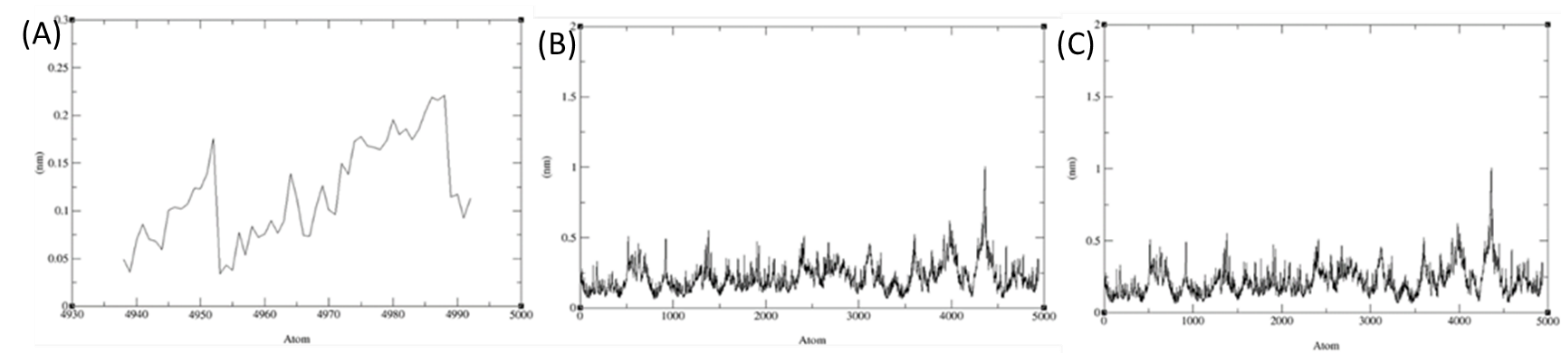


**Fig. S37.** The RMSF evaluation of compound 11 and protein 3E0M complex (A) Ligand, (B) Protein, (C) Protein and Ligand complex; at 305K, , during the 5 ns MD simulation.


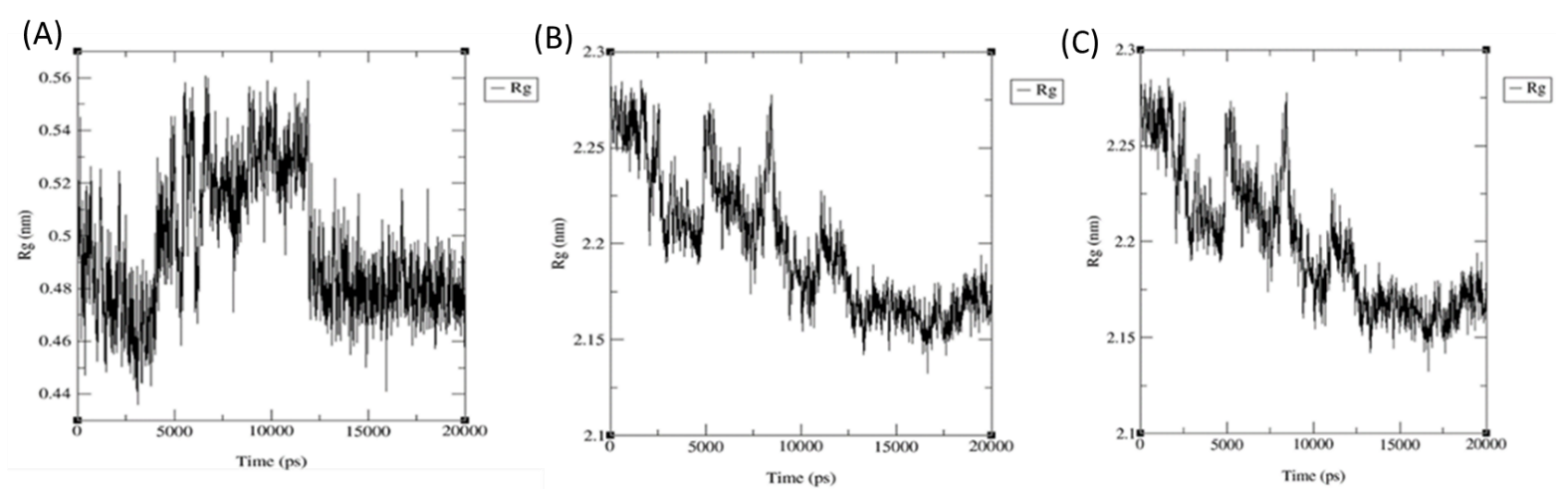


**Fig. S38.** The Gyration evaluation of compound 11 and protein 3E0M complex (A) Ligand, (B) Protein, (C) Protein and Ligand complex; at 305K, , during the 20 ns MD simulation.


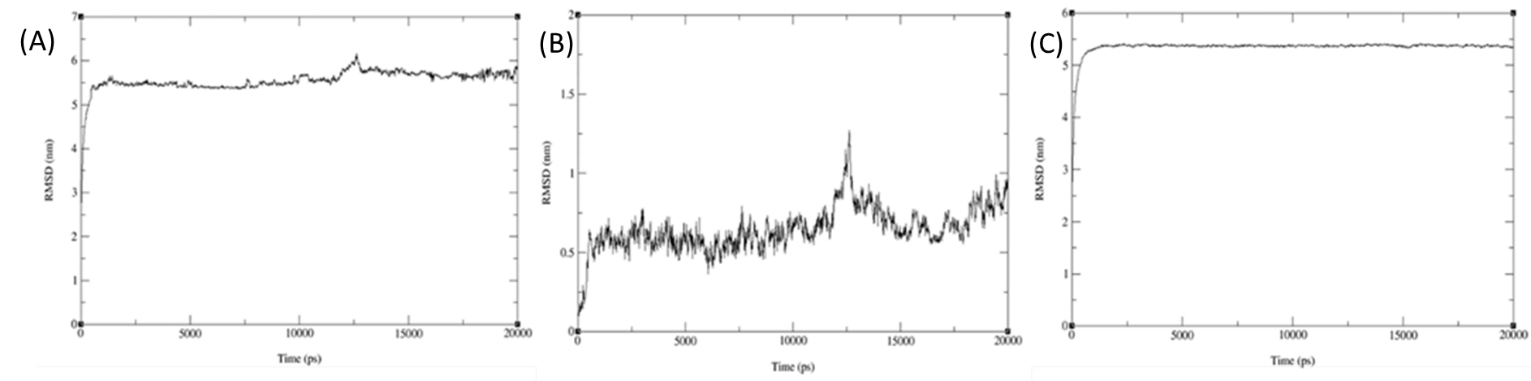


**Fig. S39.** The RMSD evaluation of compound 11 and protein 3E0M complex (A) Ligand, (B) Protein, (C) Protein and Ligand complex; at 310K, , during the 20 ns MD simulation.


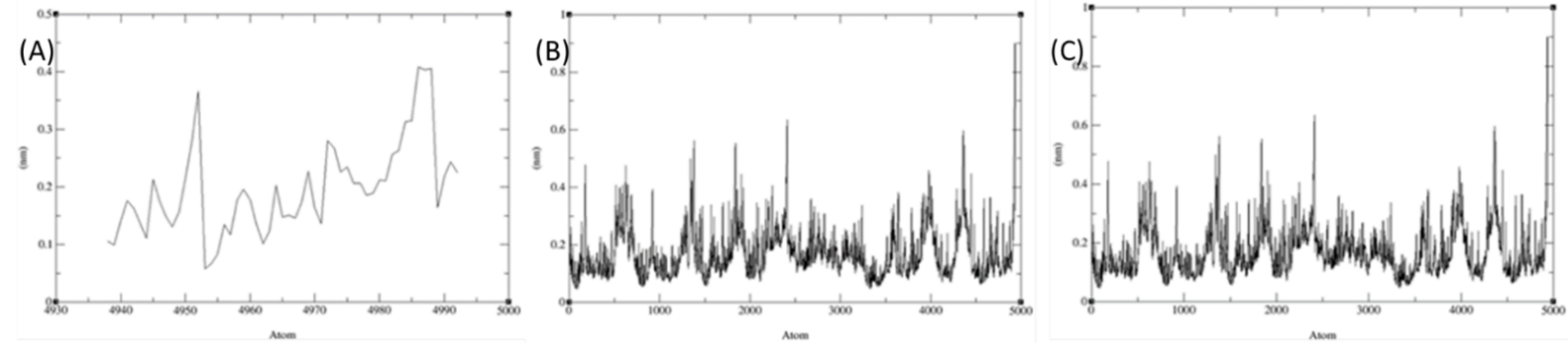


**Fig. S40.** The RMSF evaluation of compound 11 and protein 3E0M complex (A) Ligand, (B) Protein, (C) Protein and Ligand complex; at 310K, , during the 5 ns MD simulation.


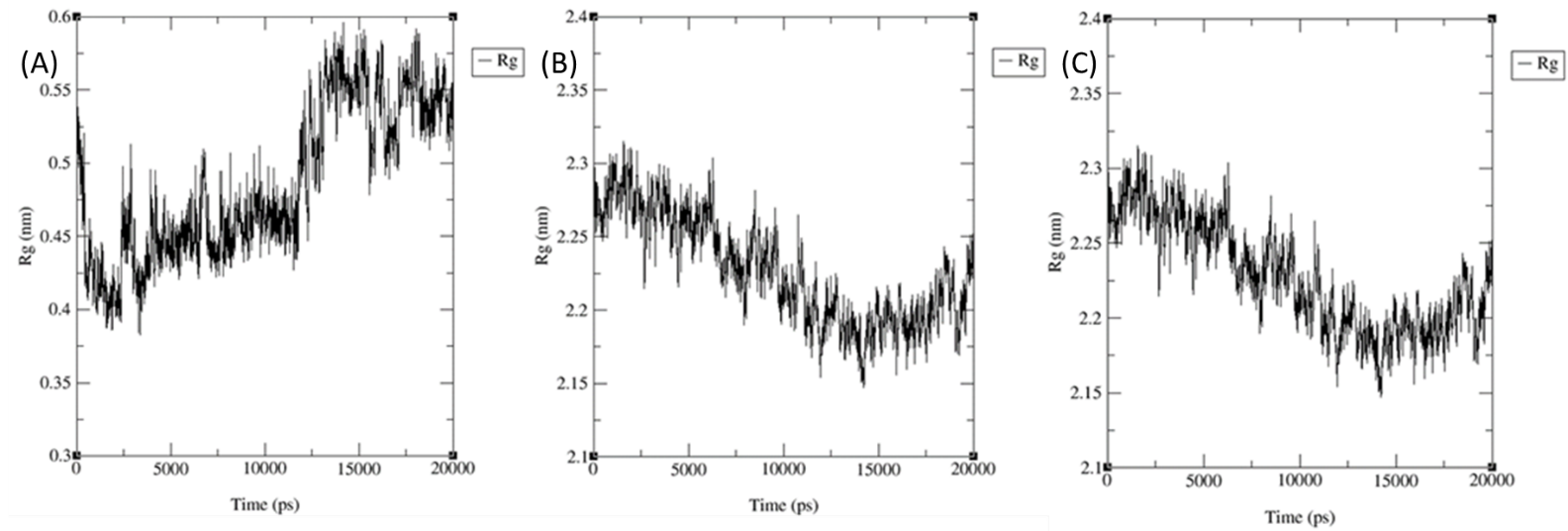


**Fig. S41.** The Gyration evaluation of compound 11 and protein 3E0M complex (A) Ligand, (B) Protein, (C) Protein and Ligand complex; at 310K, , during the 20 ns MD simulation.


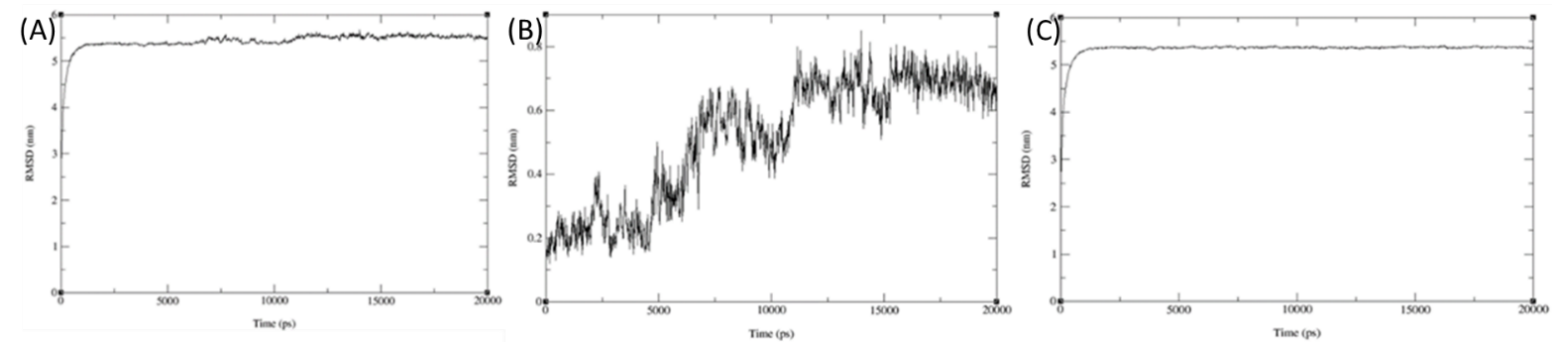


**Fig. S42.** The RMSD evaluation of compound **11** and protein 3E0M complex (A) Ligand, (B) Protein, (C) Protein and Ligand complex; at 320K, , during the 20 ns MD simulation.


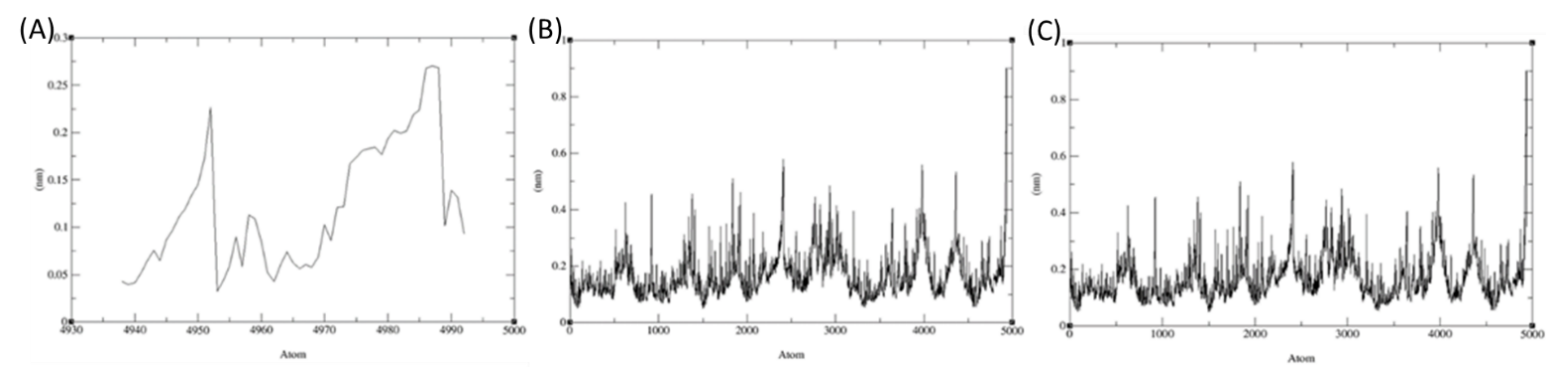


**Fig. S43.** The RMSF evaluation of compound 11 and protein 3E0M complex (A) Ligand, (B) Protein, (C) Protein and Ligand complex; at 320K, , during the 5 ns MD simulation.


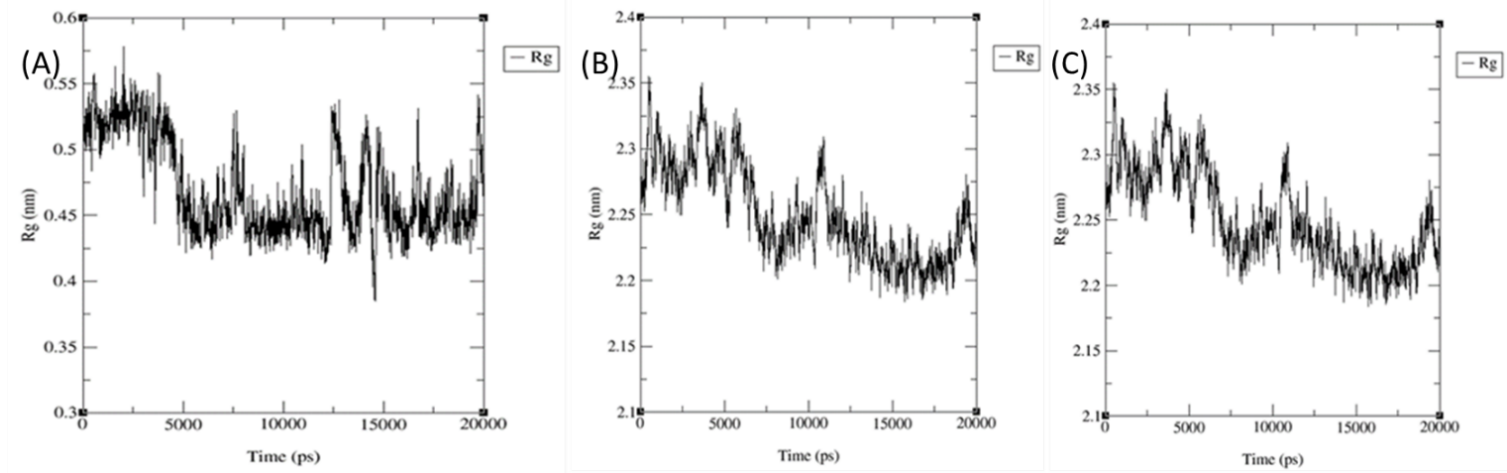


**Fig. S44.** The Gyration evaluation of compound 11 and protein 3E0M complex (A) Ligand, (B) Protein, (C) Protein and Ligand complex; at 320K, , during the 20 ns MD simulation.


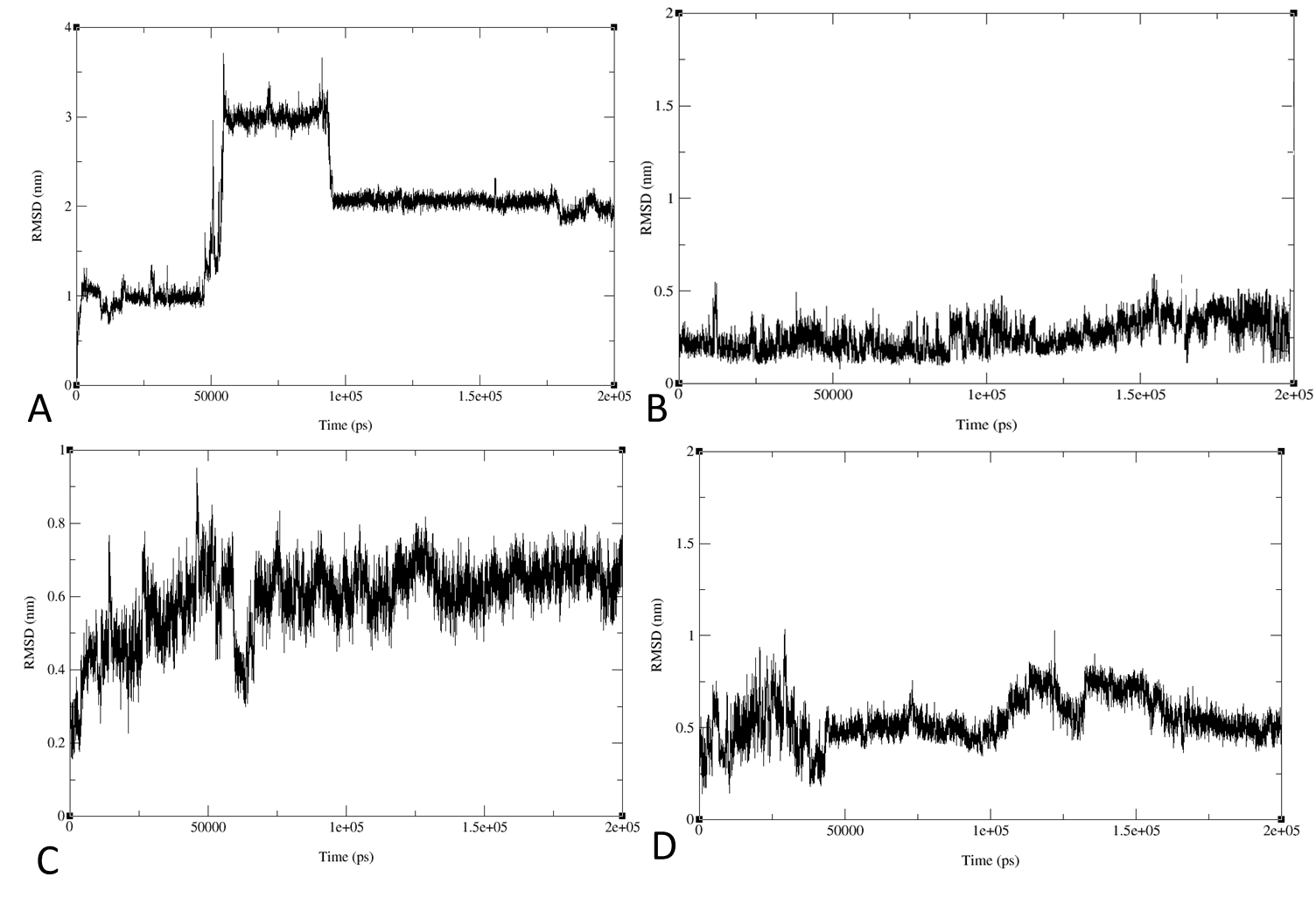


**Fig. S45.** RMSD evaluation of ligand-protein complexes for compounds **9**, **11**, **13**, and ofloxacin at 300 K during 200 ns MD simulations.


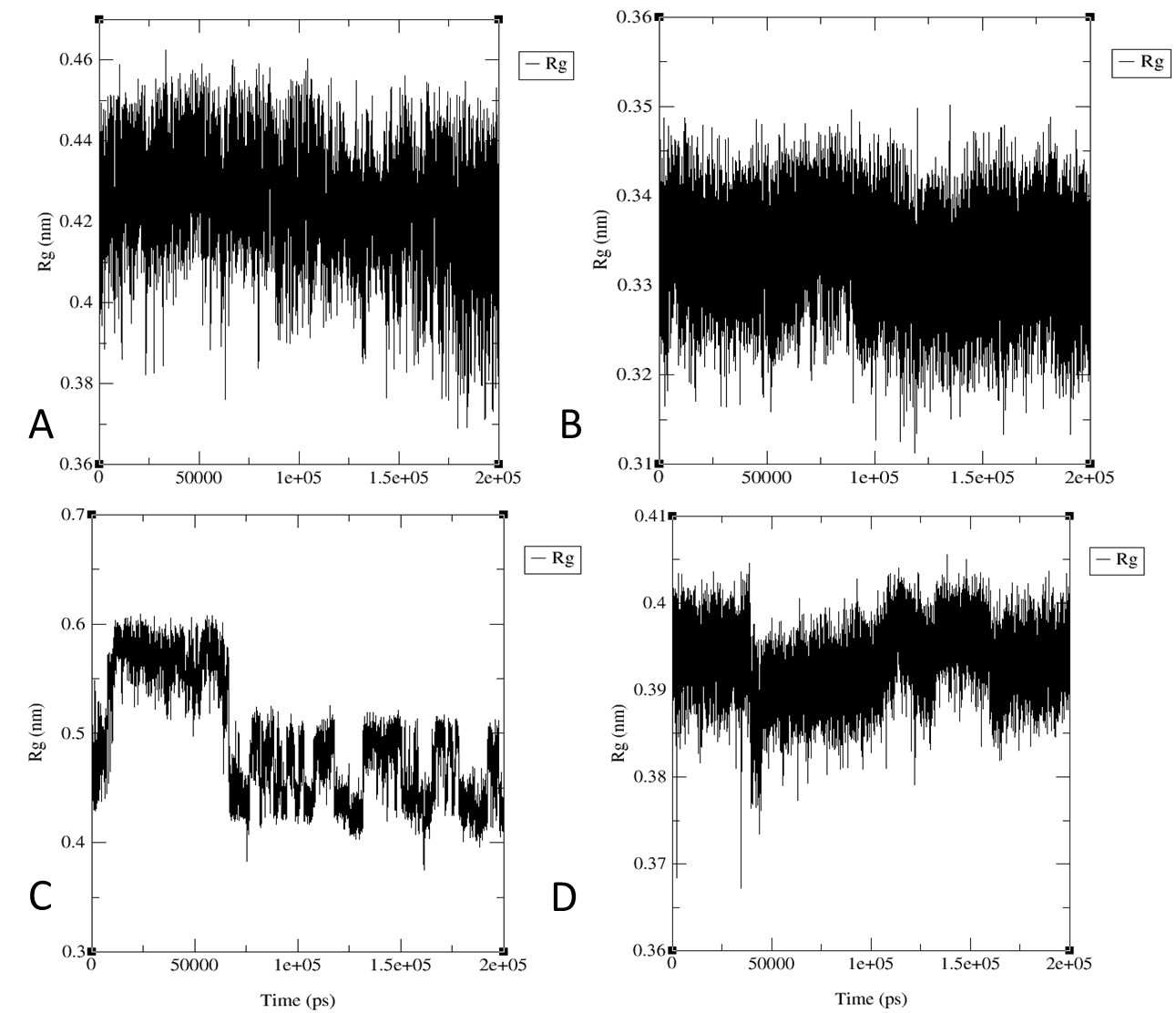


**Fig. S46.** RMSF evaluation of ligand-protein complexes for compounds **9**, **11**, **13**, and ofloxacin at 300 K during 200 ns MD simulations.


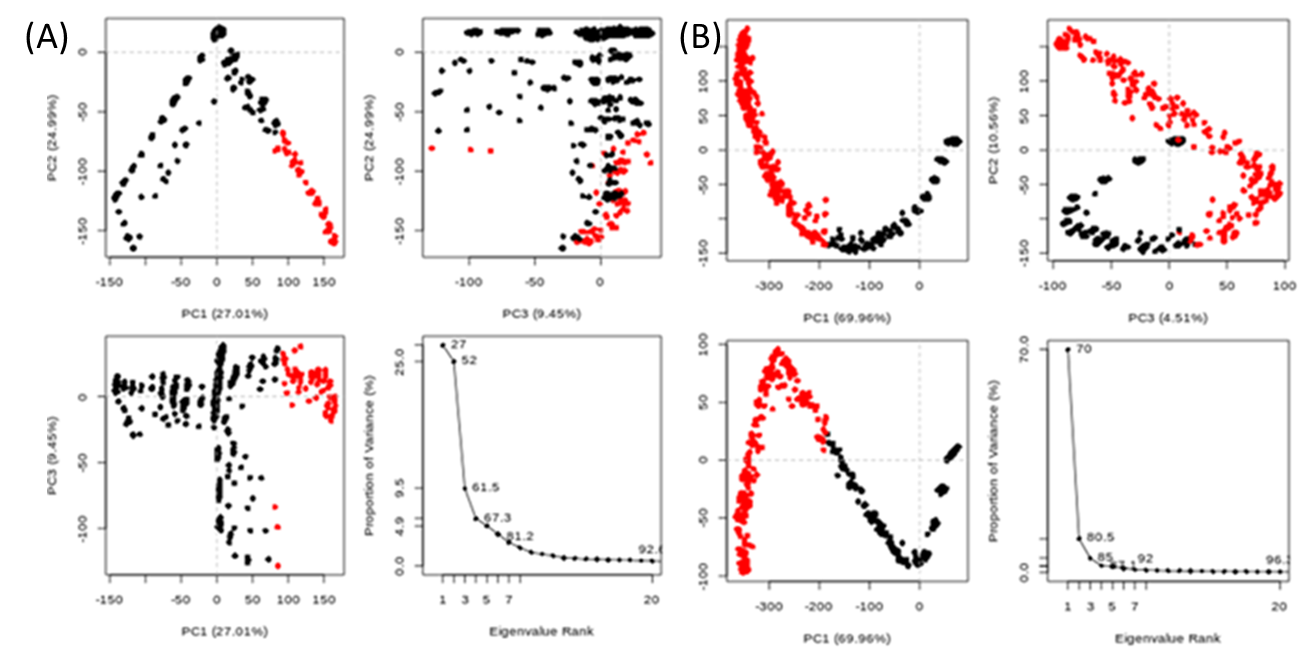


**Fig. S45.** PCA of the MD trajectory of the complexes (A) (Ligand 9+3E0M), (B) (Ligand 11+3E0M), at 300K. Black dots indicate the energetically unstable conformational state, and red dots indicate the stable conformational state.


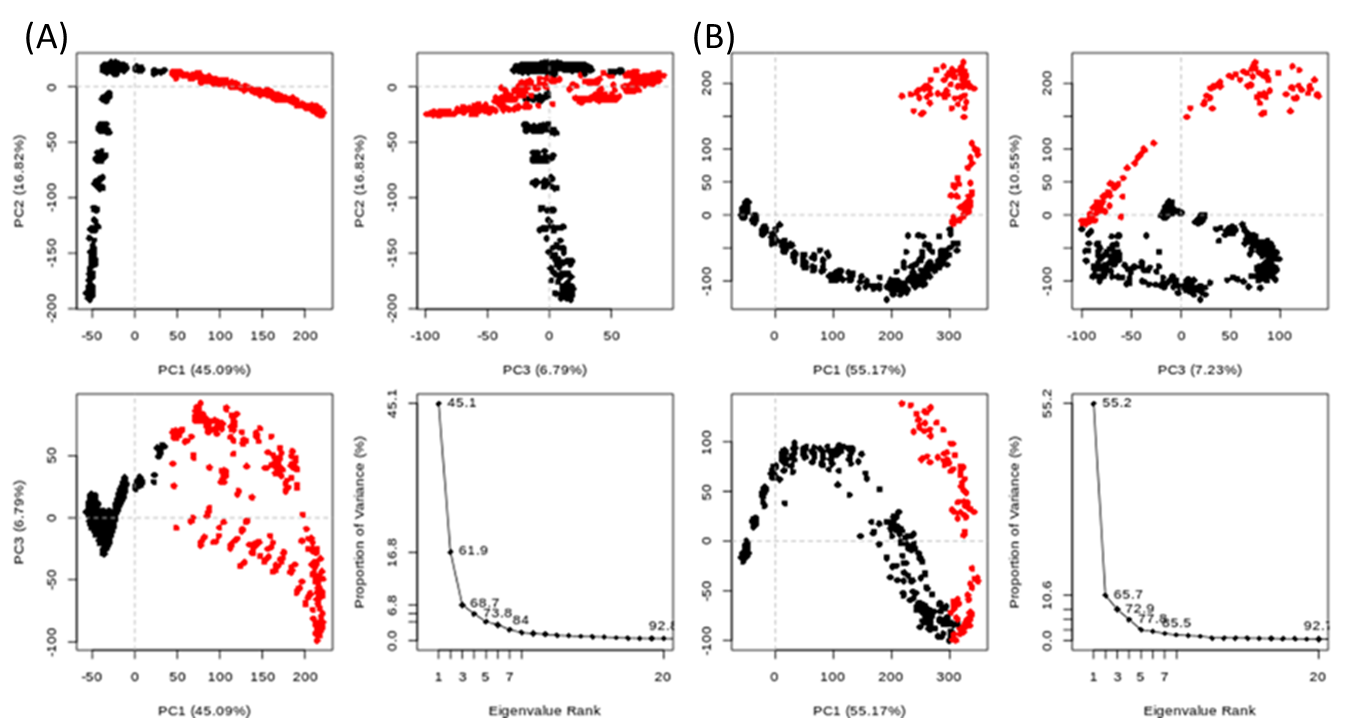


**Fig. S46.** PCA of the MD trajectory of the complexes (A) (Ligand 13+3E0M), and (B) (Ofloxacin+3E0M), at 300K. Black dots indicate the energetically unstable conformational state, and red dots indicate the stable conformational state.


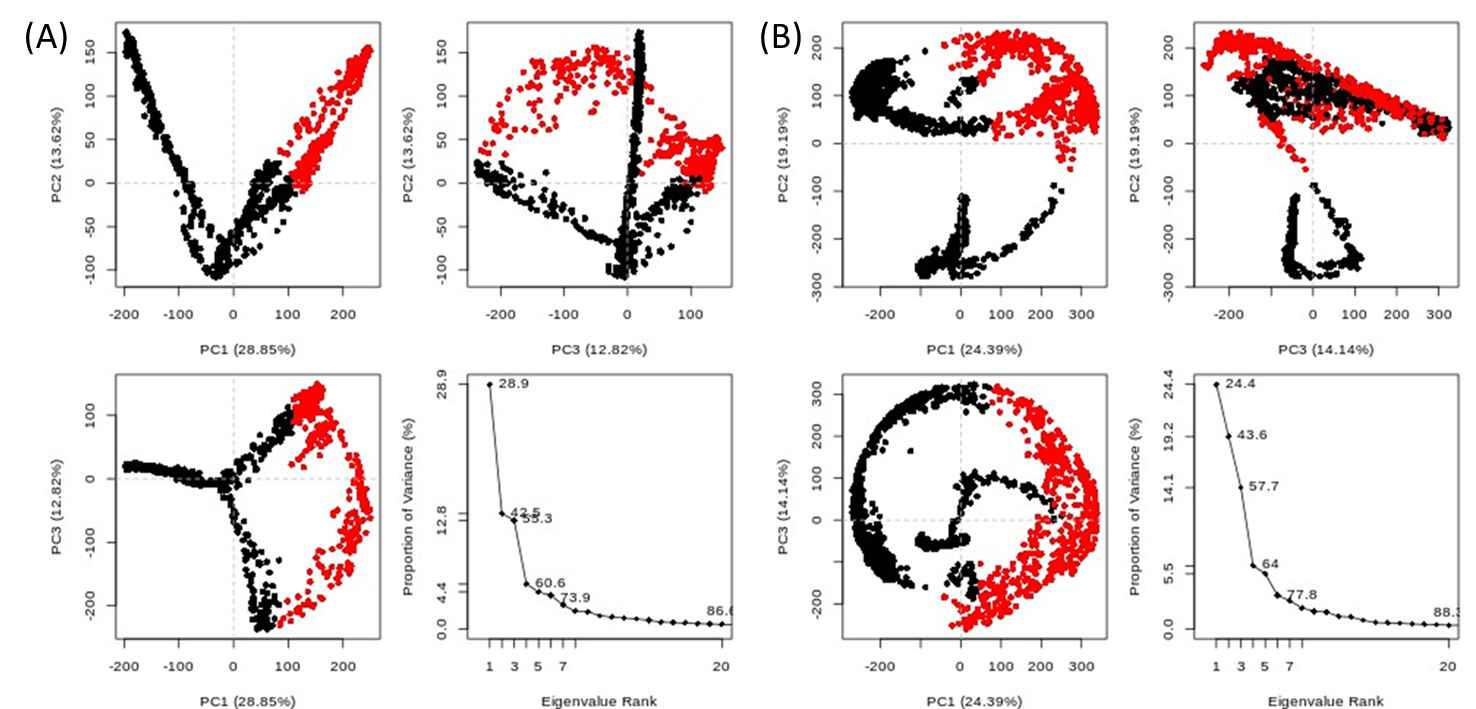


**Fig. S47.** PCA of the MD trajectory of the Ligand 11 with 3E0M complex (A) at 305K and (B) 310k. Black dots indicate the energetically unstable conformational state, and red dots indicate the stable conformational state.


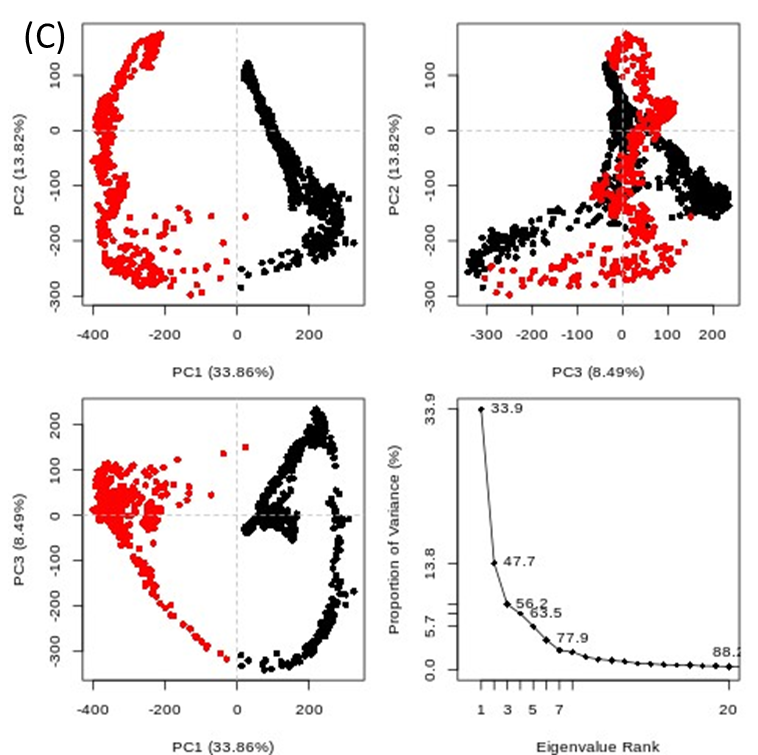


**Fig. S48.** PCA of the MD trajectory of the Ligand 11 with 3E0M complex PCA Cluster Plots (C) at 320K. Black dots indicate the energetically unstable conformational state, and red dots indicate the stable conformational state.
